# Photocatalytic reverse polarity Povarov reaction†
†Electronic supplementary information (ESI) available. CCDC 1831373. For ESI and crystallographic data in CIF or other electronic format see DOI: 10.1039/c8sc01704b


**DOI:** 10.1039/c8sc01704b

**Published:** 2018-07-06

**Authors:** Jamie A. Leitch, Angel L. Fuentes de Arriba, Joanne Tan, Oskar Hoff, Carlos M. Martínez, Darren J. Dixon

**Affiliations:** a Department of Chemistry , Chemical Research Laboratory , University of Oxford , 12 Mansfield Road , Oxford , UK . Email: darren.dixon@chem.ox.ac.uk; b Davenport Research Laboratories , Department of Chemistry , University of Toronto , 80 St. George Street , Toronto , ON , Canada; c Janssen Research and Development , C/Rio Jarama, 75A , Toledo , Spain

## Abstract

A visible light mediated iridium photocatalysed reverse polarity Povarov reaction of aryl imines and electron deficient alkenes is described. Operating *via* a putative nucleophilic α-amino radical, generated by a proton coupled electron transfer process, addition to a range of conjugated electron deficient alkene substrates affords substituted tetrahydroquinoline products in high yields and with typically good to excellent diastereoselectivity in favor of the *trans* diastereoisomer. Sub-stoichiometric quantities of Hantzsch ester were found to be key to initiate the overall redox-neutral, free radical cyclization cascade. This new reaction complements existing two electron Lewis acid mediated variants and expands the capabilities of imine umpolung chemistry to synthetically relevant cyclisation methodology.

## Abstract 2

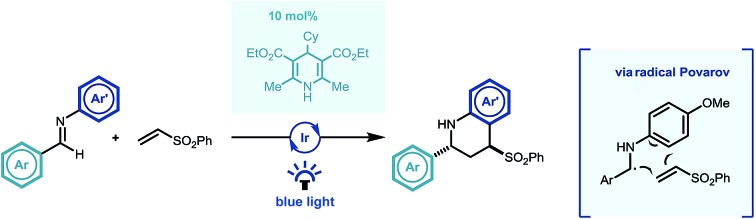
A reverse polarity photocatalysed Povarov reaction of imines and electron deficient alkenes is described.

## Introduction

Photoredox catalysis, through its ability to generate reactive radical intermediates under mild yet highly tunable reaction conditions and experimental set-ups,^1^ offers great potential for new reaction discovery.^2^ Pioneering developments have significantly expanded the synthetic toolbox^3^ and have been applied in the construction of building blocks^4^ and natural products,^5^ and also in the derivatization of biologically relevant molecules^6^ and macromolecular peptide structures.^7^

Photocatalytic approaches to the synthesis and functionalisation of amines and their derivatives – with direct impact on medicinal chemistry programmes^8^ – have been particularly prevalent.^9^ Recent efforts have focussed on the photocatalytic generation and trapping of α-amino radicals derived from a range of starting materials.^10^ Noteworthy examples include single electron transfer (SET) decarboxylation of amino acid derivatives,^11^ direct hydrogen atom transfer (HAT) of aliphatic amines,^12^ or *via* the SET reduction of imine derivatives.^13^ The resulting α-amino radicals have been shown to subsequently engage in radical–radical coupling reactions,^14^ partake in transition metal catalysed cross coupling processes or react with a range of electrophilic species.^15^

**Scheme 1:**
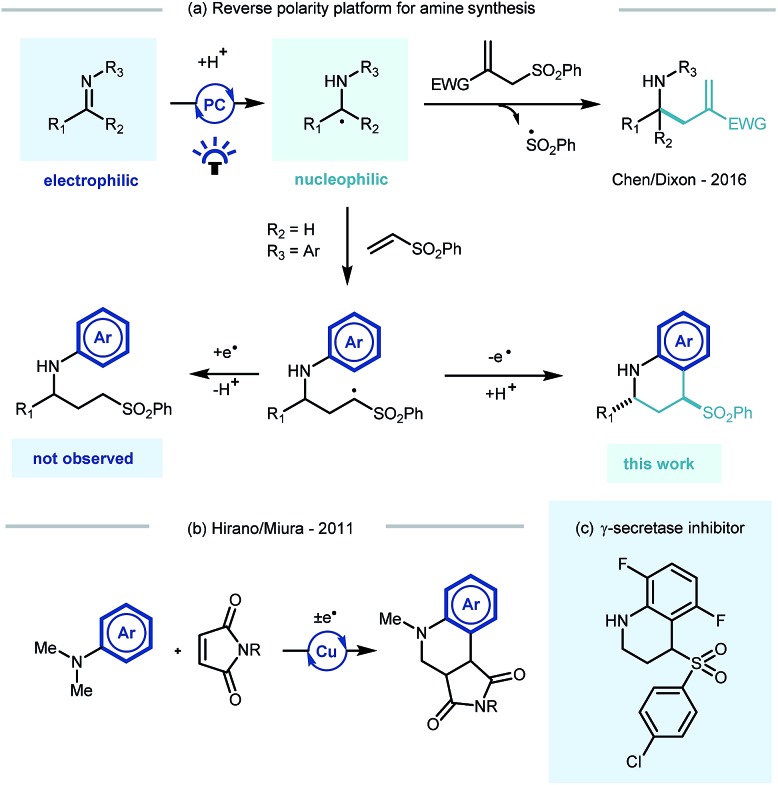
Previous reports in the context of this work. PC = photocatalyst.

Building on Knowles' seminal studies on proton coupled electron transfer (PCET) for the generation of α-heteroatom radicals,13*a* our group recently reported the photocatalytic reductive coupling of imines with allyl sulfone electrophiles using Eosin Y as the photocatalyst, and Hantzsch ester as the stoichiometric reductant, under green LED light irradiation.15*b* Contemporaneously Chen,15*a* and later Ngai,15*c* reported similar reactivity of *in situ* generated α-amino radicals from imines. Such a reversal of the natural imine polarity *via* the PCET manifold establishes a new umpolung approach for the synthesis of α-functionalised amines (Scheme 1a), and creates many opportunities for new reaction discovery.

In a continuation of our programme, we sought to explore and expand the synthetic utility of photocatalytic imine umpolung chemistry. Our previous work demonstrated that the α-allylated amine product was the outcome of the reaction with Michael acceptors bearing a sulfone leaving group.15*b* With prospective coupling partners not possessing a suitable leaving group it was unclear as to what the outcome of the reaction would be. Following nucleophilic addition, the intermediate γ-amino radical could either gain a further electron and proton to form the Michael addition product,15*a* or potentially cyclize onto the pendant electron rich aromatic ring and subsequently rearomatize (Scheme 1a). Both pathways would be synthetically useful but the latter would allow the direct construction of the biologically relevant tetrahydroquinoline scaffold, examples of which have shown to possess biological activity in γ-secretase inhibitors (Scheme 1c)^16^ and androgen agonists/antagonists.^17^ Previous studies by Hirano and Miura on the copper catalysed redox coupling of *N*,*N*-dimethylaniline and maleimide derivatives provided some precedent for the cyclisation pathway (Scheme 1b)^18^ and encouraged us to investigate along these lines of enquiry. Herein, we wish to report our findings.

Preliminary studies were carried out using fluorine tagged aldimine (**1a**), phenyl vinyl sulfone (**2a**, 3 equiv.) as the Michael acceptor, (Ir[dF(CF_3_)ppy]_2_(dtbbpy))PF_6_ (**[Ir]**, 1 mol%) as photocatalyst and the commercial Hantzsch ester (**HE1**, 1.2 equiv.) as a stoichiometric reductant, in DMSO, under blue LED light irradiation. Pleasingly, good reactivity was identified early and importantly cyclised product **3a** – a reverse polarity Povarov product^19^ – was formed as the sole coupling product in the reaction mixture (Table 1, entry 1) as a 7 : 1 mixture of diastereomers. Despite the typical use of Hantzsch esters as a superstoichiometric reductive quencher in photoredox coupling reactions of imines,^20^,^21^ our redox-neutral process benefited from the use of 60 mol% **HE1**. This was largely due to suppression of over-reduction and aza-pinacol side products (entry 2) and the use of substituted Hantzsch esters (**HE4** & **HE6**, entries 3 & 4) further suppressed their formation.^16^,^22^ Further reduction of the Hantzsch ester loading to 10 mol% led to lower reaction conversions (entry 5) using blue LED irradiation, however by switching to a commercial photoreactor, full conversion was achieved in 16 hours and the product was isolated in excellent yield (90%) as a 10 : 1 mixture of diastereomers. The reaction methodology was also amenable to a reduction in equivalents of vinyl sulfone coupling partner without major impact on reaction efficiency (entry 7) and reactivity was completely lost on removal of Hantzsch ester (entry 8), or iridium photocatalyst (entry 9), and in the absence of light (entry 10).

**Table 1:** Optimization of photocatalytic reverse polarity Povarov reaction

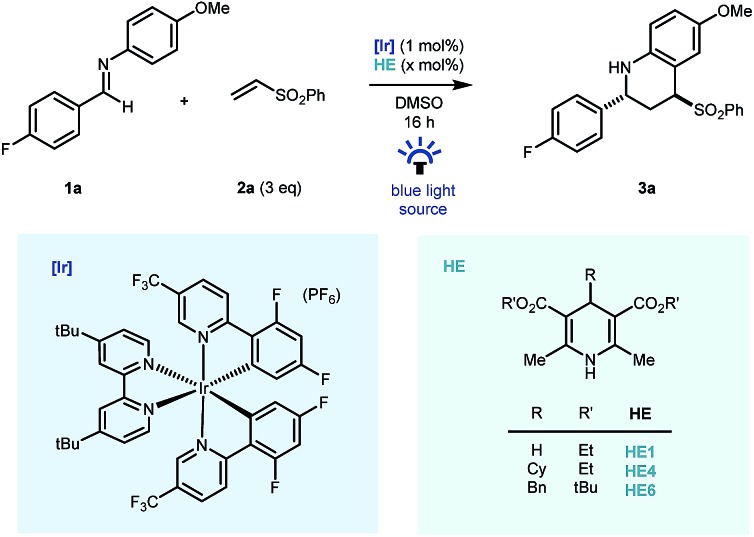				
Entry	HE (*x* mol%)	Light source	**3a** ^*c*^	dr
1	**HE1** (120 mol%)	LED bath	64	7 : 1
2	**HE1** (60 mol%)	LED bath	84	7 : 1
3	**HE4** (60 mol%)	LED bath	100 (56)^*d*^	10 : 1
4	**HE6** (60 mol%)	LED bath	99	11 : 1
5	**HE4** (10 mol%)	LED bath	54	8 : 1
**6**	**HE4** **(10 mol%)**	**Photoreactor**	**95 (90)** ^*d*^	**10** **:** **1**
**7** ^*e*^	**HE4** **(10 mol%)**	**Photoreactor**	**93 (88)** ^*d*^	**10** **:** **1**
8^*e*^	—	Photoreactor	—	—
9^*e*^ ^,^^*f*^	**HE4** (10 mol%)	Photoreactor	—	—
10^*e*^	**HE4** (10 mol%)	—	—	—

^*a*^General reaction conditions: **1a** (0.25 mmol), phenyl vinyl sulfone (1.25 mmol, 5 eq.), (Ir[dF(CF_3_)ppy]_2_(dtbbpy))PF_6_ (0.0025 mmol), Hantzsch ester derivatives (*x* mol%), DMSO (1 mL).

^*b*^For further details on blue light source see ESI.

^*c*^ ^19^F NMR yield determined by direct conversion between **1a** and **3a** (major + minor diastereoisomer) including by-products.

^*d*^Isolated yield after silica gel column chromatography.

^*e*^2 eq. phenyl vinyl sulfone used.

^*f*^Without the iridium catalyst.

**Scheme 2:**
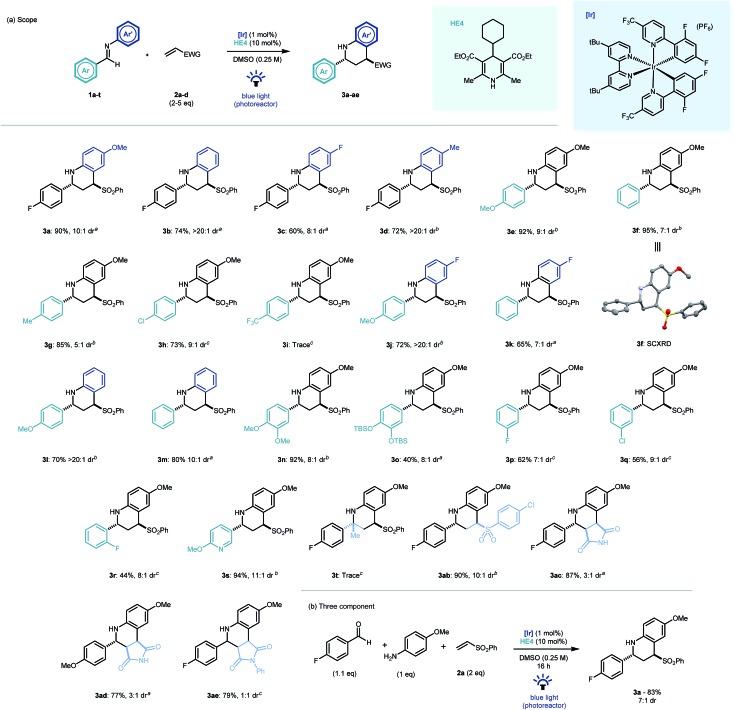
Scope of the photocatalytic reverse polarity Povarov reaction. Reaction time: ^*a*^16 h, ^*b*^40 h, ^*c*^64 h.

With optimal conditions established, we looked to probe the scope of the photocatalytic reverse polarity Povarov reaction (Scheme 2a). Initially, the tolerance to variation on the aniline portion of the aldimine was investigated. Pleasingly, the reaction proceeded well with a number of substituents in the 4-position (**3b–d**). Good reaction efficiency and excellent diastereoselectivity towards the *trans* diastereoisomeric product was noted in all cases.^23^ This is in contrast to Brønsted or Lewis acid catalysed Povarov reactions in which *cis*-configured stereoisomeric products often predominate.^19^ When variations to the aromatic ring of the aldehyde moiety were explored, electronics were found to play a pivotal role, with electron rich and neutral arenes giving excellent yields (**3e–g**, **3j–n**) whereas electron poor aromatics led to longer reaction times (**3h**) and even complete nullification of reactivity (**3i**). 3-Fluoro and 2-fluoro-substituted aldimines were tolerated in this chemistry however longer reaction times were required and yields diminished with proximity to the imine C=N bond (**3p–r**). A substituted pyridyl substrate was also shown to be effective in the reaction mixture leading to an excellent yield of product (**3s**).

Disappointingly, ketimines were not found to be viable substrates for this reaction (**3t**) even after prolonged reaction times. 4-Chlorophenyl vinyl sulfone was found to be an excellent electrophile (**3ab**). Similarly, maleimide and *N*-phenylmaleimide electrophiles were shown to be excellent coupling partners in the new cyclisation methodology, however diastereoselectivity was respectively reduced or absent in the reaction products (**3ac–ae**). The chemistry was extendable to a three component one-pot process (Scheme 2b) and demonstrated the expedient construction of complex tetrahydroquinoline structures using simple building blocks, as well as its potential application to modular library synthesis.

**Scheme 3:**
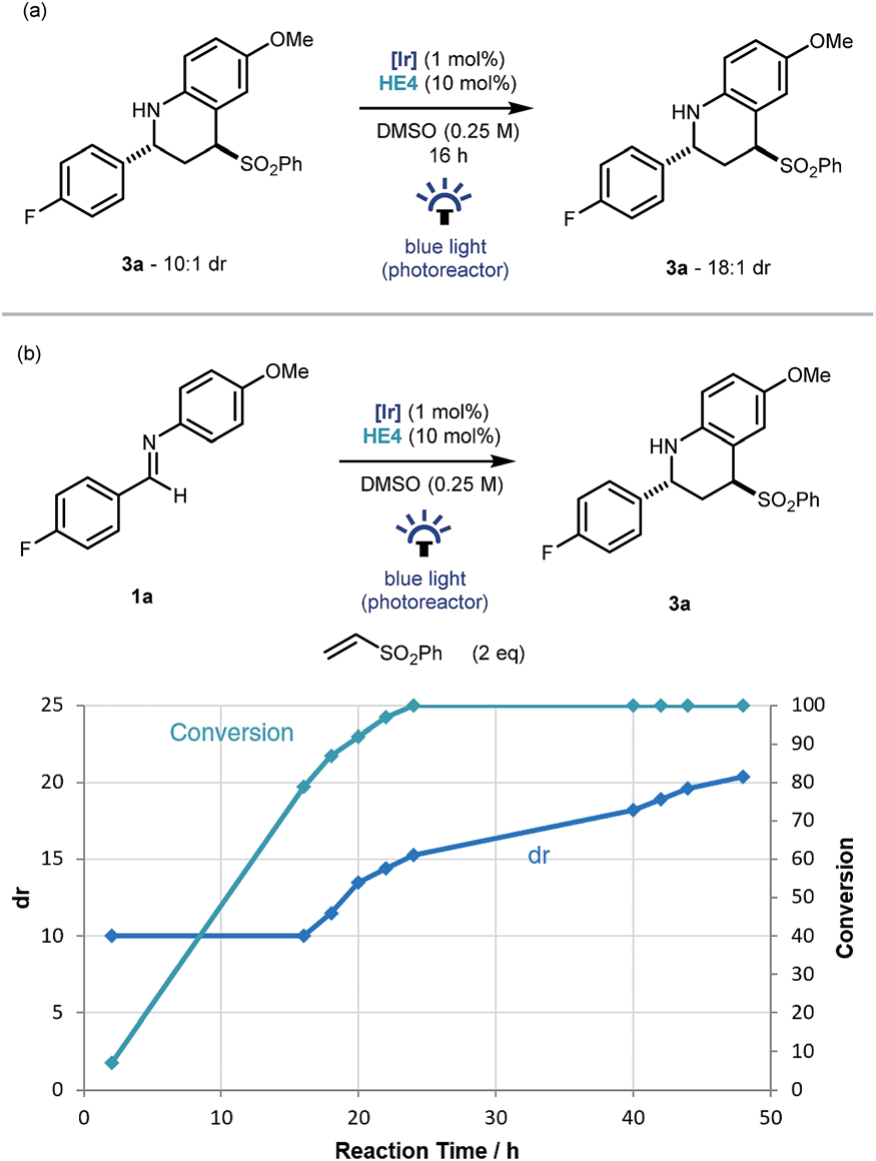
Investigation into the diastereoselectivity of the reaction.

An interesting observation made throughout the optimisation studies was that after the imine substrate **1a** was consumed in the reaction vessel, product diastereomeric ratio continued to increase, suggesting that epimerization was occurring under the reaction conditions. To investigate this further, Povarov product **3a** (dr 10 : 1) was resubmitted to the reaction conditions. Pleasingly an increase to 18 : 1 was observed and mass balance was maintained (Scheme 3a). As this epimerization does not take place without the iridium catalyst, or in the absence of light, a photoredox based mechanism for the formation of a planar α-amino radical is proposed.^24^ Monitoring the reaction over time from initiation using ^19^F NMR spectroscopy, demonstrated that the formation of **3a** had an intrinsic kinetic dr of ∼10 : 1 in favor of the *trans* diastereoisomer. Then from the point of ∼90% conversion, the diastereoselectivity increased gradually up to 20 : 1 after 48 hours (Scheme 3b).

**Scheme 4:**
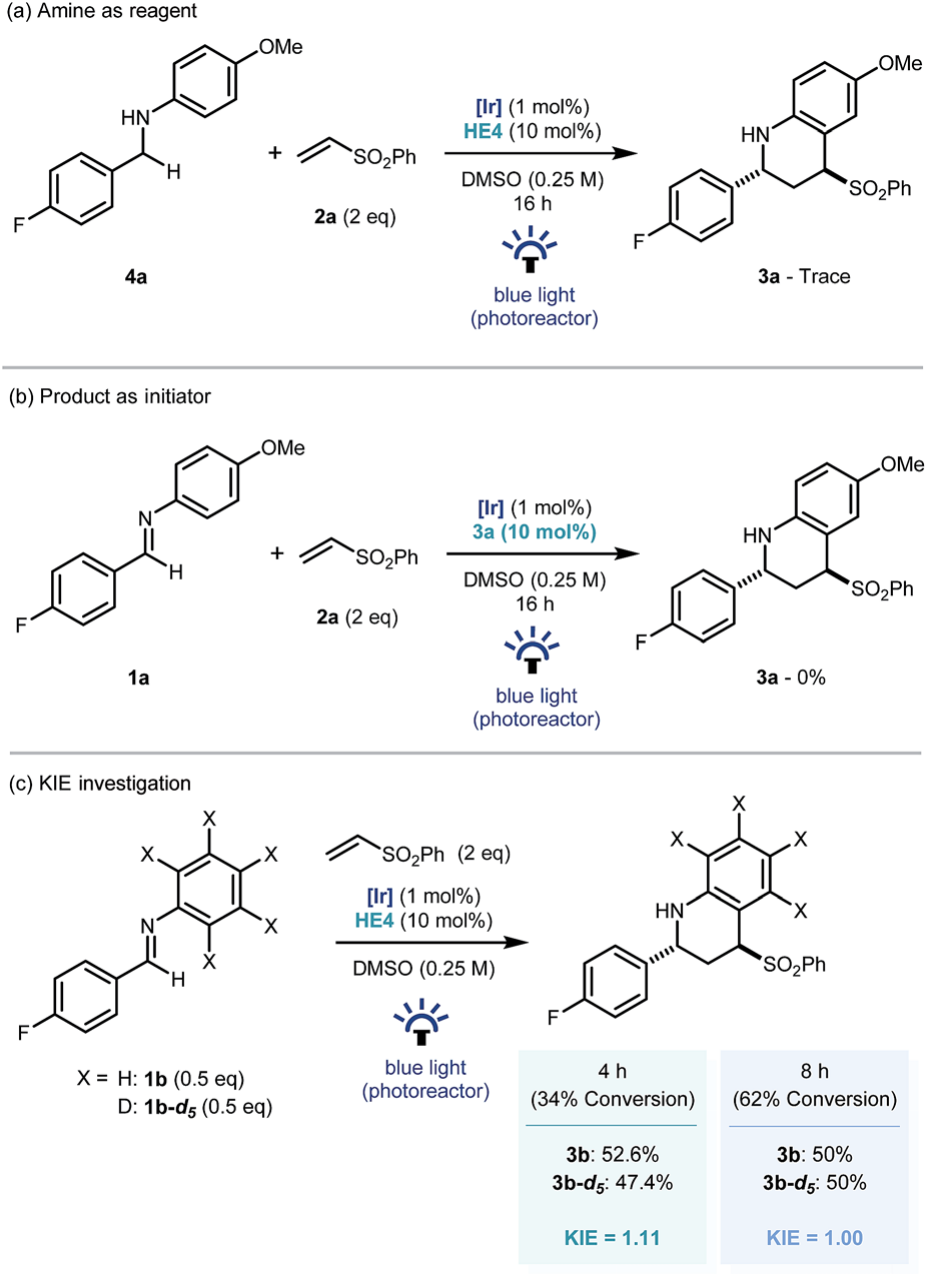
Control and mechanistic experiments.

Recent investigations have shown that imines (*E*01/2 = –1.90 V *vs.* SCE in CH_3_CN),^25^ can be reduced more readily using proton-coupled electron transfer. Chen's,15*a* ours,15*b* and Ngai's15*c* previous investigations have demonstrated that partially oxidized Hantzsch esters are acidic enough to be used as proton donors in PCET mechanisms.^26^ To probe this further, the reaction was repeated using the reduced amine – which is a by-product of the reaction – as starting material (**4a**, Scheme 4a). Only trace amounts of product were observed, suggesting the reverse polarity Povarov reaction does not proceed *via* initial reduction of the imine. This reinforces the proposal of PCET construction of the key α-amino radical.

**Scheme 5:**
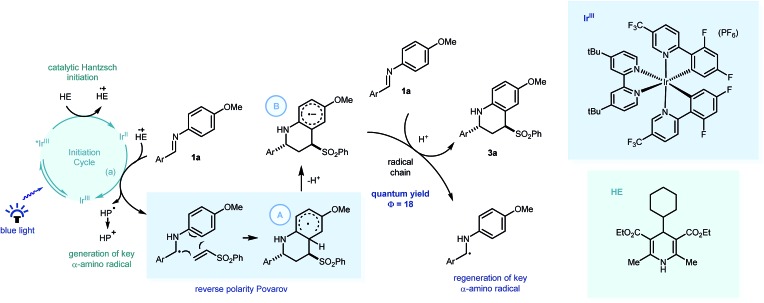
Postulated mechanism for the visible light mediated reverse polarity Povarov reaction with key PCET processes highlighted. HE = Hantzsch ester, HP = Hantzsch pyridine.

As only sub stoichiometric quantities of the Hantzsch ester are needed for full conversion, its role is likely as an initiator in a self-propagating mechanism. We were intrigued to identify whether either the cyclised product **3a** or an intermediate could act as a reductive quencher to propagate further product formation. To this end, we carried out a control experiment where **HE4** (10 mol%) was replaced with **3a** (Scheme 4b). Importantly, no product formation was observed suggesting that an intermediate – not the product of the reaction – is capable of propagating this reaction mechanism. Kinetic isotope effect (KIE) studies demonstrated that the isotopic sensitivity of selectivity determining steps has a value of ∼1, suggesting that the substrates are kinetically committed toward product formation prior to any C–H cleavage events (Scheme 5c).^27^

From these insights, we propose that PCET would enable Ir[dF(CF_3_)ppy]_2_(dtbbpy)PF_6_ (*E*01/2 = –1.37 V *vs.* SCE in CH_3_CN)1*a* to reduce the imine readily to form the key α-amino radical (Scheme 5) which can react with the phenyl vinyl sulfone in a step-wise radical cyclization to give the stabilised Povarov radical intermediate **A**. As this methodology only requires sub-stoichiometric quantities of the substituted Hantzsch ester (**HE4**), we suggest this Povarov radical intermediate **A** can lose a proton (kinetically facile) to form radical anion intermediate **B**, in a base assisted homolytic aromatic substitution-type mechanism.^28^ Using Yoon's method, we calculated the quantum yield of the reaction between **1a** and **2a**, to be 18.^29^ This suggests that a radical chain process is in operation, with the strongly reducing radical anion intermediate able to reduce fresh imine substrate with concomitant protonation,^30^ thus forming the product (**3a**) and regenerating the key α-amino radical.

## Conclusions

In conclusion we have developed a new photocatalytic reverse polarity Povarov reaction to construct decorated tetrahydroquinolines in high yield and diastereoselectivity. This polarity reversal was postulated to stem from an α-amino radical formed *via* the PCET of imine derivatives. 10 mol% of a Hantzsch ester was found to be optimal as a reductive initiator, a feature which has not previously been disclosed in photoredox catalysis. Further investigations are ongoing to establish further coupling partners and scaffolds for this reverse polarity platform for the synthesis of α-functionalised amines.

## Conflicts of interest

There are no conflicts to declare.

## Supplementary Material

Supplementary information

Crystal structure data
