# Supplementary material for: Photocatalytic reverse polarity Povarov reaction
Source: Chem Sci. 2018 Jul 6;9(32):6653–8. doi: 10.1039/c8sc01704b (PMC6115624; doi:10.1039/c8sc01704b)
Supplement: Supplementary file 1 [file SC-009-C8SC01704B-s001.pdf]

Electronic Supplementary Material (ESI) for Chemical Science.
This journal is © The Royal Society of Chemistry 2018

Supporting Information

For

Photocatalytic Reverse Polarity Povarov Reaction

Jamie A. Leitch,[a] Angel L. Fuentes de Arriba,[a] Joanne Tan,[b] Oskar Hoff,[a] Carlos M. Martínez,[c] and Darren J. Dixon.*,[a]

[a] : Department of Chemistry, Chemical Research Laboratory, University of Oxford, 12 Mansfield Road, Oxford, UK.

[b] : Davenport Research Laboratories, Department of Chemistry, University of Toronto, 80 St. George Street, Toronto, ON, Canada

[c] : Janssen Research and Development, C/Rio Jarama, 75A, Toledo, Spain

* Email : darren.dixon@chem.ox.ac.uk

Contents

1. General S3

2. Reactor Set-Ups S4

3. Preliminary Experiments S6

4. Optimisation S7

5. Synthesis of Starting Materials S8

6. Synthesis of Cyclised Products S38

7. Mechanistic Experiments S62

    7.1: Diastereoselectivity S62

    7.2: Luminescence Quenching Studies S63

    7.3: Light Dark Experiments S64

    7.4: KIE Experiments S65

    7.5: Quantum Yield Determination S66

    7.6: Plausible Epimerisation Mechanism S69

8. NMR Spectra S70

9. References S107

1. General

Proton, carbon and fluorine NMR spectra were recorded on Bruker 400 MHz (^{1}H NMR at 400 MHz, ^{13}C NMR at 101 MHz, and ^{19}F NMR at 377 MHz). Chemical shifts for protons are reported in parts per million downfield from $Si(CH_3)_4$ and are referenced to residual protium in the deuterated solvent ($CHCl_3$ at 7.26 ppm, DMSO at 3.31 (H_2O), 2.50 depending on solvent used). Chemical shifts for fluorines are reported in parts per million downfield from $CFCl_3$. NMR data are presented in the following format: chemical shift (multiplicity [app = apparent, br = broad, d = doublet, t = triplet, q = quartet, dd = doublet of doublets), dt = doublet of triplets), dq = doublet of quartets), ddd = doublet of doublet of doublets), m = multiplet], coupling constant [in Hz], number of equivalent nuclei by integration).

High-resolution mass spectra (ESI) were recorded on Bruker µTOF mass spectrometer. Infrared spectra were recorded on a Bruker Tensor 27 FT-IR spectrometer as a thin film. Only selected maximum absorbances are reported (in ν_{max} (cm^{-1})). Melting points were obtained on a Leica Galen III Hot-stage melting point apparatus and microscope and on a Kofler hot block and are reported uncorrected. Analytical thin-layer chromatography (TLC) was performed on Merck silica gel 60 F254 plates and visualised with UV light (254 or 365 nm), and/or $KMnO_4$. Silica gel column chromatography was performed using 60 Å silica gel 40-63 µm purchased from VWR. Samples were dried onto silica gel prior to addition to column.

All reactions were performed using reagents obtained from Sigma-Aldrich, Acros Organics, Alfa Aesar, STREM or Fluorochem without further purification unless stated. All water used was purified through a Merck Millipore reverse osmosis purification system prior to use. Anhydrous toluene, tetrahydrofuran, dichloromethane and diethyl ether were dried by filtration through activated alumina (powder ~150 mesh, pore size 58 Å, basic, Sigma-Aldrich) columns and stored under an atmosphere of N_2 prior to use. Dimethyl sulfoxide (anhydrous) and dimethylformamide (anhydrous) were used as supplied. Deuterated solvents were used as supplied. Reactions were performed in under a balloon of N_2 if not stated. Temperatures quoted are external. Solvents were removed under reduced pressure using Büchi Rotavapor apparatus.

2. Reactor Set-Ups

1. LED Strip "Loop" Set-Up

12V LED strips wrapped around in coils and fastened with cable ties. Up to 3 reactions at one time, leads to more variable results.

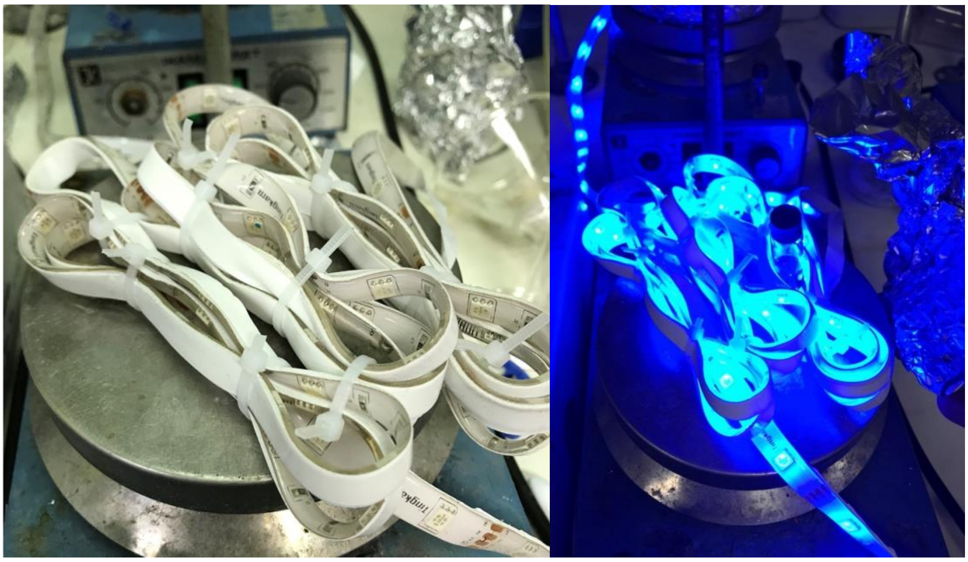

2. LED Strip "Bath" Set-Up

12V LED strips wrapped around the inside of a crystallising dish, with foil encased around the outside and top when reactions taking place. Reactions were placed in the centre of the dish, one reaction at a time. Leads to more accurate results but lower throughput.

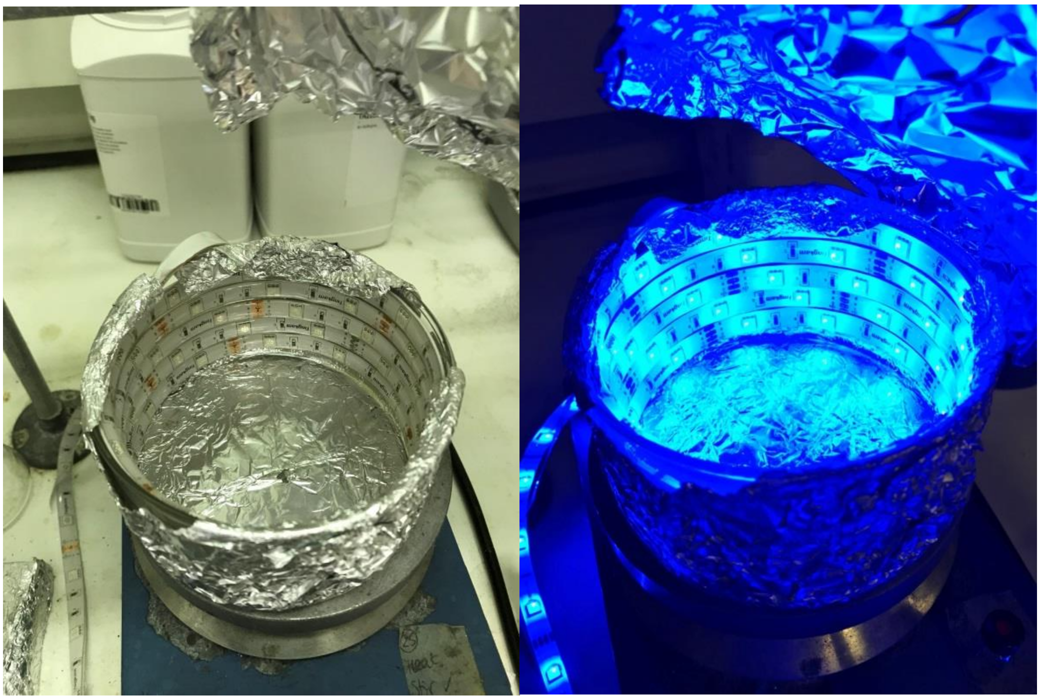

3. Photoreactor

Hepatochem PhotoRedOx Box, equipped with a Kessil 40W LED light. A cardboard cover was also placed over the reactor during reactions. Capable of carrying out up to 8 reactions at one time. Accurate and reproducible results, high throughput.

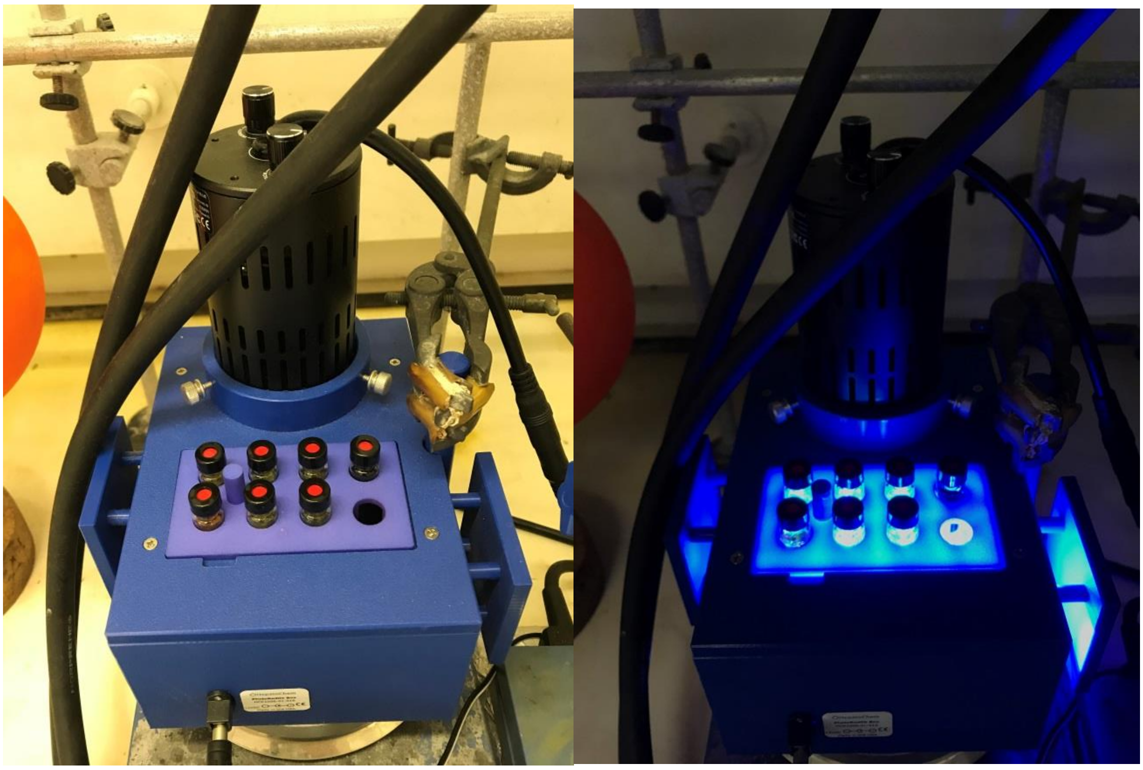

3. Preliminary Experiments

Investigations were initiated using slightly modified conditions from our previous report,[1] utilising Eosin Y as photocatalyst and the commercial Hantzsch ester as stoichiometric reductant.

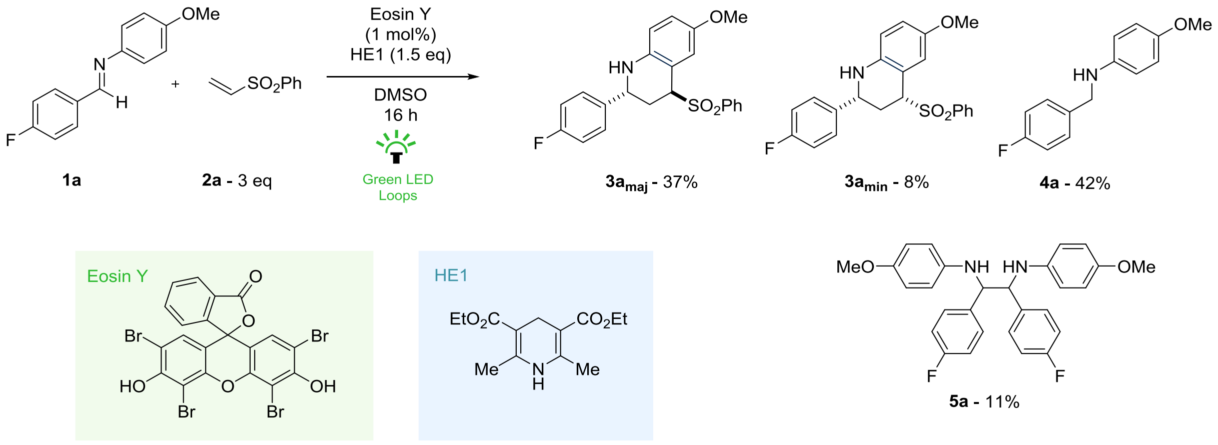

Pleasingly the formation of cyclised was observed in modest NMR yields, with both diastereomers formed under the reaction conditions (dr 5:1). A large quantity of the over-reduced amine was used which is assumed to form from background reaction between the imine and the Hantzsch ester. To circumvent this 1.2 eq of Hantzsch ester was used in the methodology in order to suppress this by-product.

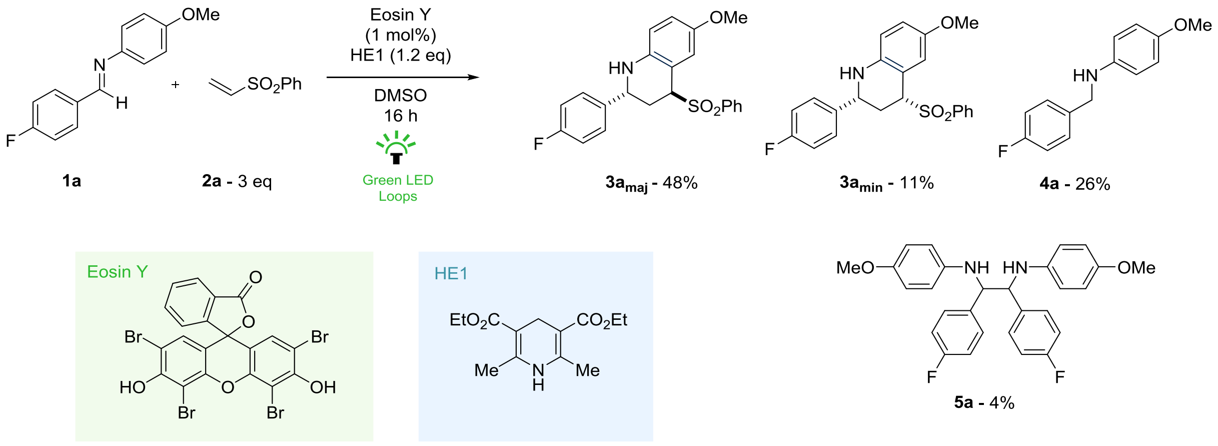

This investigation led to reduced yield of the **4a** and increased formation of the cyclised products (**3a**$_{maj}$ & **3a**$_{min}$). From this point we were interested in changing the photocatalyst to Ru(bpy)$_3$ and using blue light for photoexcitation. The results from this point are given in the optimization table.

4. Optimisation

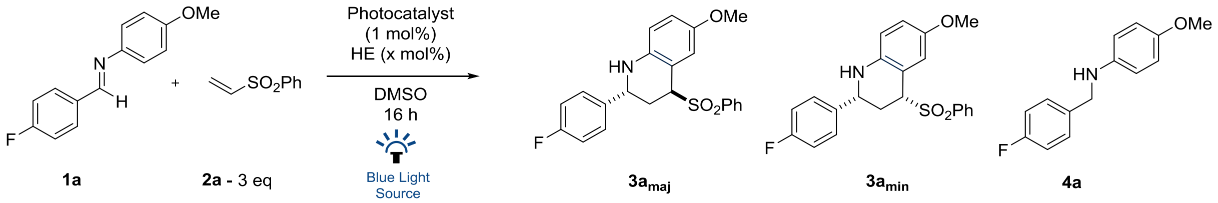

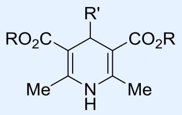

HE	R	R'
1	Et	H
2	Et	*i*Pr
3	Et	Ph
4	Et	Cy
5	Me	Cy
6	*t*Bu	Bn

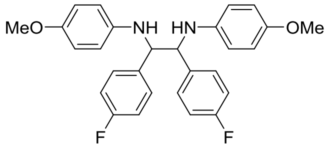

5a

Entry	Photocatalyst	HE	x mol%	Light Source	$3a_{maj}$	$3a_{min}$	$3a_{total}$	dr	4a	5a
1	$[Ru(bpy)_3]Cl_2$	1	120	Loop	64	10	74	6:1	24	2
2	$[Ru(bpy)_3]Cl_2$	1	60	Loop	64	10	74	6:1	8	17
3	$[Ru(bpz)_3](PF_6)_2$	1	60	Loop	3	1	4	3:1	1	1
4	$[Ru(bpy)_3](PF_6)_2$	1	60	Loop	80	11	91	8:1	0	8
5	$[Ir(ppy)_2(dtbbpy]PF_6$	1	60	Loop	70	11	81	7:1	5	8
6	$[Ir(dF(CF_3)ppy)_2(dtbbpy)]PF_6$	1	60	Loop	81	11	92	7:1	5	9
7	Eosin Y[a]	1	60	Loop	69	12	81	6:1	6	12
8	4-CzIPN	1	60	Loop	67	11	78	6:1	6	11
9	$[Ir(dF(CF_3)ppy)_2(dtbbpy)]PF_6$	1	60	Bath	82	12	94	7:1	5	1
10	$[Ir(dF(CF_3)ppy)_2(dtbbpy)]PF_6$	1	120	Bath	59	10	69	7:1	30	-
11	$[Ir(dF(CF_3)ppy)_2(dtbbpy)]PF_6$	2	60	Bath	75	5	80	15:1	-	16
12	$[Ir(dF(CF_3)ppy)_2(dtbbpy)]PF_6$	3	60	Bath	76	12	88	6:1	6	6
13	$[Ir(dF(CF_3)ppy)_2(dtbbpy)]PF_6$	4	60	Bath	91[a]	9	100 (56)[b]	10:1	-	-
14	$[Ir(dF(CF_3)ppy)_2(dtbbpy)]PF_6$	5	60	Bath	61	8	69	8:1	-	-
15	$[Ir(dF(CF_3)ppy)_2(dtbbpy)]PF_6$	6	60	Bath	91	8	99	11:1	-	-
16	$[Ir(dF(CF_3)ppy)_2(dtbbpy)]PF_6$	4	10	Bath	50	4	54	8:1	-	-
**17**	$\mathbf{[Ir(dF(CF_3)ppy)_2(dtbbpy)]PF_6}$	**4**	**10**	**Photoreactor**	**85**	**10**	**95 (90)[b]**	**10:1**	**-**	**-**
**18[c]**	$\mathbf{[Ir(dF(CF_3)ppy)_2(dtbbpy)]PF_6}$	**4**	**10**	**Photoreactor**	**84**	**9**	**93 (86)[b]**	**10:1**	**-**	**-**
19[c]	-	4	10	Photoreactor	Trace	-	-	-	-	-
20[c]	$[Ir(dF(CF_3)ppy)_2(dtbbpy)]PF_6$	4	-	Photoreactor	-	-	-	-	-	-
21[c]	$[Ir(dF(CF_3)ppy)_2(dtbbpy)]PF_6$	4	10	-	-	-	-	-	-	-

a : Green LEDs were used. b: : Isolated yield after silica column chromatography. c : 2 eq phenyl vinyl sulfone was used (to facilitate purification).

5. Synthesis of Starting Materials

General Procedure **A** for the Synthesis of *N*-Arylaldimines.

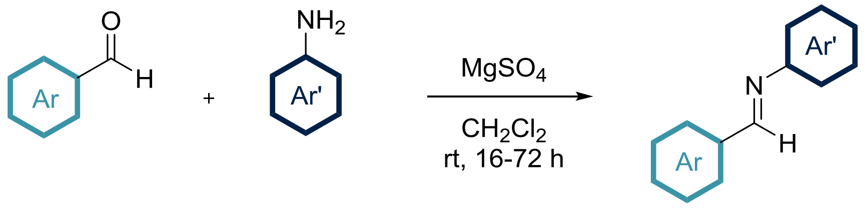

A 25 mL round bottomed flask was charged with CH_2Cl_2 (10 mL) and $MgSO_4$ (~1 g) and allowed to stir for 5 minutes at rt. To the flask was added relevant aldehyde (10 mmol) and aniline (10 mmol). The flask was sealed and purged with nitrogen for 10 minutes. The needle was removed and a the reaction was allowed to stir under a balloon of nitrogen for 16 hours. After this time the mixture was diluted further with CH_2Cl_2 and filtered through a pad of cotton wool. The filtrate was concentrated *in vacuo* to give crude aldimine. The compounds were either used crude as ^{1}H NMR showed >95% purity, or purified *via* trituration with pentane, or recrystallization from EtOH or CH_2Cl_2/pentane.

Synthesis of **1a**

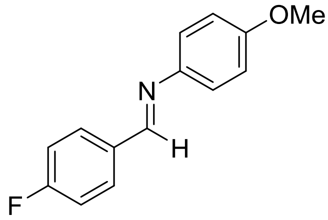

The above compound was prepared according to General Procedure **A** using 4-fluorobenzaldehyde (1.07 mL, 1.24 g, 10 mmol) and *p*-anisidine (1.23 g, 10 mmol). Recrystallization from CH_2Cl_2/pentane gave a pale lavender solid, 50% (1.15 g). **^{1}H NMR** (400 MHz, $CDCl_3$) δ 8.44 (s, 1H), 7.89 (dd, *J* = 8.6, 5.6 Hz, 2H), 7.25 – 7.20 (m, 2H), 7.19 – 7.11 (m, 2H), 6.97 – 6.89 (m, 2H), 3.83 (s, 3H). **^{19}F NMR** (377 MHz, $CDCl_3$) δ -108.60 – -108.77 (m). **^{13}C NMR** (101 MHz, Chloroform-*d*) δ 164.5 (d, *J* = 251.2 Hz), 158.4, 156.8, 144.7, 132.8, 130.5 (d, *J* = 8.6 Hz), 122.2, 115.9 (d, *J* = 22.0 Hz), 114.4, 55.5. Data was consistent with literature precedent.[2]

Synthesis of **1b**

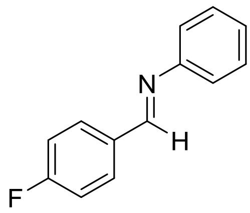

The above compound was prepared according to General Procedure **A** using 4-fluorobenzaldehyde (1.07 mL, 1.24 g, 10 mmol) and aniline (0.91 mL, 0.93 g, 10 mmol). The compound was used crude as >95% purity was confirmed *via* ^{1}H NMR, off-white solid, 95% (1.89 g). **^{1}H NMR** (400 MHz, $CDCl_3$) δ 8.32 (s, 1H), 7.85 – 7.76 (m, 2H), 7.30 (dd, *J* = 8.3, 7.4 Hz, 2H), 7.18 – 7.09 (m, 3H), 7.06 (t, *J* = 8.6 Hz, 2H). **^{19}F NMR** (377 MHz, $CDCl_3$) δ -108.02 (ddd, *J* = 13.9, 8.7, 5.5 Hz). **^{13}C NMR** (101 MHz, $CDCl_3$) δ 164.8 (d, *J* = 252.5 Hz), 158.9, 152.0, 132.7 (d, *J* = 3.2 Hz), 130.9 (d, *J* = 8.9 Hz), 129.3, 126.1, 121.0, 116.0 (d, *J* = 22.1 Hz). Data was consistent with literature precedent.[3]

Synthesis of **1c**

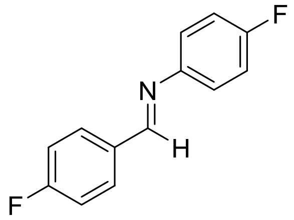

The above compound was prepared according to General Procedure **A** using 4-fluorobenzaldehyde (1.07 mL, 1.24 g, 10 mmol) and 4-fluoroaniline (0.95 mL, 1.11 g, 10 mmol). The compound was used crude as >95% purity was confirmed *via* ^{1}H NMR, off-white solid, 89% (1.93 g). **^{1}H NMR** (400 MHz, $CDCl_3$) δ 8.40 (s, 1H), 7.95 – 7.81 (m, 2H), 7.24 – 7.12 (m, 4H), 7.12 – 7.00 (m, 2H). **^{19}F NMR** (377 MHz, $CDCl_3$) δ -107.88 (tt, *J* = 8.1, 5.1 Hz), -116.76 – -117.46 (m). **^{13}C NMR** (101 MHz, $CDCl_3$) δ 164.7 (d, *J* = 252.1 Hz), 161.3 (d, *J* = 244.7 Hz), 158.6, 147.8, 132.4 (d, *J* = 2.6 Hz), 130.8 (d, *J* = 8.8 Hz), 122.3 (d, *J* = 8.6 Hz), 116.1 (d, *J* = 6.9 Hz), 115.8 (d, *J* = 7.2 Hz). Data was consistent with literature precedent.[4]

Synthesis of **1d**

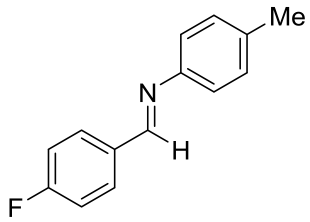

The above compound was prepared according to General Procedure **A** using 4-fluorobenzaldehyde (1.07 mL, 1.24 g, 10 mmol) and *p*-toluidine (1.24 g, 10 mmol). The compound was used crude as >95% purity was confirmed *via* ^{1}H NMR, off-white solid, 84% (1.79 g). **^{1}H NMR** (400 MHz, $CDCl_3$) δ 8.51 (s, 1H), 7.98 (dd, *J* = 8.8, 5.6 Hz, 2H), 7.30 (d, *J* = 8.4 Hz, 2H), 7.27 – 7.20 (m, 4H), 2.47 (s, 3H). **^{19}F NMR** (377 MHz, $CDCl_3$) δ -108.34 (tt, *J* = 8.9, 5.6 Hz). **^{13}C NMR** (101 MHz, $CDCl_3$) δ 164.7 (d, *J* = 251.8 Hz), 158.1, 149.3, 136.0, 132.8 (d, *J* = 3.1 Hz), 130.8 (d, *J* = 8.3 Hz), 129.9, 120.9, 115.6 (d, *J* = 22.3 Hz), 21.1. Data was consistent with literature precedent.[5]

Synthesis of **1e**

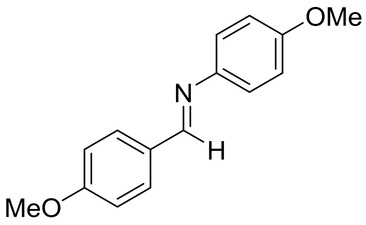

The above compound was prepared according to General Procedure **A** using *p*-anisaldehyde (1.21 mL, 1.36 g, 10 mmol) and *p*-anisidine (1.23 g, 10 mmol). The compound was used crude as >95% purity was confirmed *via* ^{1}H NMR, white solid, 78% (1.89 g). **^{1}H NMR** (400 MHz, $CDCl_3$) δ 8.43 (s, 1H), 7.86 (d, *J* = 8.8 Hz, 2H), 7.23 (d, *J* = 8.8 Hz, 2H), 7.00 (d, *J* = 8.8 Hz, 2H), 6.95 (d, *J* = 8.9 Hz, 2H), 3.89 (s, 3H), 3.85 (s, 3H). **^{13}C NMR** (101 MHz, $CDCl_3$) δ 162.1, 158.1, 158.0, 145.4, 130.4, 129.6, 122.2, 114.46, 114.3, 55.6, 55.5. Data was consistent with literature precedent.[6]

Synthesis of **1f**

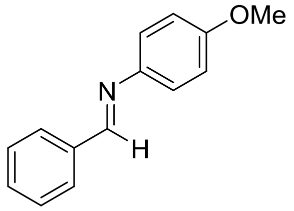

The above compound was prepared according to General Procedure **A** using benzaldehyde (1.02 mL, 1.06 g, 10 mmol) and *p*-anisidine (1.23 g, 10 mmol). The compound was used crude as >95% purity was confirmed *via* ^{1}H NMR, grey solid, 78% (1.65 g). **^{1}H NMR** (400 MHz, $CDCl_3$) δ 8.51 (s, 1H), 8.08 – 7.87 (m, 2H), 7.57 – 7.42 (m, 3H), 7.28 (d, *J* = 8.9 Hz, 2H), 6.97 (d, *J* = 8.8 Hz, 2H), 3.86 (s, 3H). **^{13}C NMR** (101 MHz, $CDCl_3$) δ 158.5, 158.4, 145.0, 136.5, 131.1, 128.8, 128.7, 122.3, 114.5, 55.6. Data was consistent with literature precedent.[7]

Synthesis of **1g**

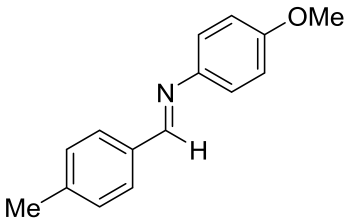

The above compound was prepared according to General Procedure **A** using 4-tolualdehyde (1.18 mL, 1.20 g, 10 mmol) and *p*-anisidine (1.23 g, 10 mmol). The compound was used crude as >95% purity was confirmed *via* ^{1}H NMR, grey solid, 72% (1.62 g). **^{1}H NMR** (400 MHz, $CDCl_3$) δ 8.35 (s, 1H), 7.69 (d, *J* = 8.2 Hz, 2H), 7.17 (d, *J* = 7.8 Hz, 2H), 7.18 – 7.09 (m, 2H), 6.91 – 6.75 (m, 2H), 3.73 (s, 3H), 2.32 (s, 3H). **^{13}C NMR** (101 MHz, $CDCl_3$) δ 158.6, 158.2, 145.2, 141.6, 134.0, 129.6, 128.7, 122.3, 114.5, 55.6, 21.7. Data was consistent with literature precedent.[8]

Synthesis of **1h**

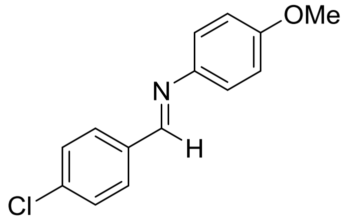

The above compound was prepared according to General Procedure **A** using 4-chlorobenzaldehyde (1.40 g, 10 mmol) and *p*-anisidine (1.23 g). The compound was purified *via* recrystallization from EtOH (200 mg, 8%). ^{1}**H NMR** (400 MHz, $CDCl_3$) δ 8.44 (s, 1H), 7.83 (d, *J* = 8.6 Hz, 2H), 7.43 (d, *J* = 8.5 Hz, 2H), 7.26 – 7.19 (m, 2H), 7.00 – 6.81 (m, 2H), 3.84 (s, 3H). ^{13}**C NMR** (101 MHz, $CDCl_3$) δ 158.6, 156.8, 144.6, 137.1, 135.1, 129.8, 129.2, 122.4, 114.6, 55.7. Data was consistent with literature precedent.[6]

Synthesis of **1i**

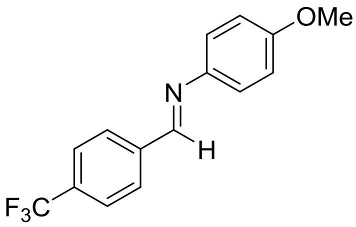

The above compound was prepared according to General Procedure **A** using 4-trifluoromethylbenzaldehyde (1.36 mL, 1.74 g, 10 mmol) and *p*-anisidine (1.23 g, 10 mmol). The compound was used crude as >95% purity was confirmed *via* ^{1}H NMR, off-white solid, 79% (2.21 g). **^{1}H NMR** (400 MHz, $CDCl_3$) δ 8.45 (s, 1H), 7.92 (d, *J* = 8.0 Hz, 2H), 7.64 (d, *J* = 8.1 Hz, 2H), 7.20 (dd, *J* = 9.2, 2.5 Hz, 2H), 7.00 – 6.79 (m, 2H), 3.77 (s, 3H). **^{19}F NMR** (377 MHz, $CDCl_3$) δ -62.74. **^{13}C NMR** (101 MHz, $CDCl_3$) δ 159.0, 156.3, 144.2, 139.7, 132.4 (q, *J* = 32.4 Hz), 128.8, 125.8 (q, *J* = 3.8 Hz), 122.5, 114.6, 55.6. Data was consistent with literature precedent.[9]

Synthesis of **1j**

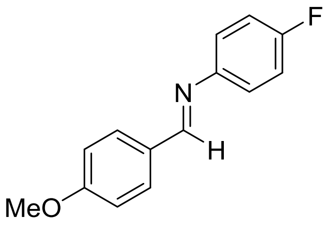

The above compound was prepared according to General Procedure **A** using *p*-anisaldehyde (1.21 mL, 1.36 g, 10 mmol) and 4-fluoroaniline (0.95 mL, 1.11 g, 10 mmol). The compound was used crude as >95% purity was confirmed *via* ^{1}H NMR, off-white solid, 74% (1.69 g). **^{1}H NMR** (400 MHz, $CDCl_3$) δ 8.36 (s, 1H), 7.84 (d, *J* = 8.8 Hz, 2H), 7.18 (dd, *J* = 9.0, 4.9 Hz, 2H), 7.07 (t, *J* = 8.7 Hz, 2H), 6.98 (d, *J* = 8.8 Hz, 2H), 3.87 (s, 3H). **^{19}F NMR** (377 MHz, $CDCl_3$) δ -117.87 (ddd, *J* = 13.1, 8.4, 5.0 Hz). **^{13}C NMR** (101 MHz, $CDCl_3$) δ 162.3, 161.0 (d, *J* = 244.3 Hz), 159.5, 148.4 (d, *J* = 3.1 Hz), 130.5, 129.1, 122.2 (d, *J* = 8.2 Hz), 115.8 (d, *J* = 21.8 Hz), 114.2, 55.4. Data was consistent with literature precedent.[10]

Synthesis of **1k**

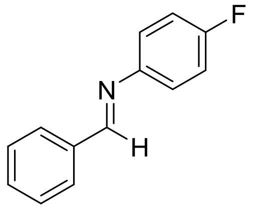

The above compound was prepared according to General Procedure **A** using benzaldehyde (1.02 mL, 1.06 g, 10 mmol) and 4-fluoroaniline (0.95 mL, 1.11 g, 10 mmol). The compound was used crude as >95% purity was confirmed *via* ^{1}H NMR, off-white solid, 83% (1.65 g). **^{1}H NMR** (400 MHz, $CDCl_3$) δ 8.45 (s, 1H), 7.98 – 7.80 (m, 2H), 7.49 (dd, *J* = 5.2, 2.0 Hz, 3H), 7.24 – 7.19 (m, 2H), 7.17 – 7.00 (m, 2H). **^{19}F NMR** (377 MHz, $CDCl_3$) δ -117.26 (ddd, *J* = 13.1, 8.1, 4.9 Hz). **^{13}C NMR** (101 MHz, $CDCl_3$) δ 161.4 (d, *J* = 244.7 Hz), 160.3, 148.2 (d, *J* = 3.1 Hz), 136.2, 131.6, 128.92, 128.90, 122.4 (d, *J* = 8.0 Hz), 116.0 (d, *J* = 22.6 Hz). Data was consistent with literature precedent. [11]

Synthesis of **1l**

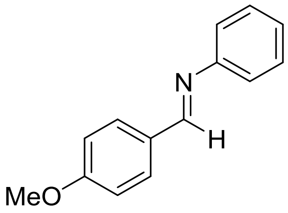

The above compound was prepared according to General Procedure **A** using *p*-anisaldehyde (1.21 mL, 1.36 g, 10 mmol) and aniline (0.91 mL, 0.93 g, 10 mmol). The compound was used crude as >95% purity was confirmed *via* ^{1}H NMR, pale canary yellow solid, 79% (1.67 g). **^{1}H NMR** (400 MHz, $CDCl_3$) δ 8.39 (s, 1H), 7.97 – 7.77 (m, 2H), 7.39 (dd, *J* = 8.3, 7.3 Hz, 2H), 7.25 – 7.16 (m, 3H), 7.09 – 6.89 (m, 2H), 3.88 (s, 3H). **^{13}C NMR** (101 MHz, $CDCl_3$) δ 162.4, 159.8, 152.5, 130.6, 129.4, 129.23, 129.20, 125.7, 125.7, 121.0, 114.3, 55.55. Data was consistent with literature precedent.[12]

Synthesis of **1m**

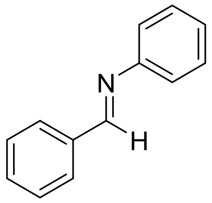

The above compound was prepared according to General Procedure **A** using benzaldehyde (1.02 mL, 1.06 g, 10 mmol) and aniline (0.91 mL, 0.93 g, 10 mmol). The compound was used crude as >95% purity was confirmed *via* ^{1}H NMR, pale canary yellow solid, 89% (1.61 g). **^{1}H NMR** (400 MHz, $CDCl_3$) δ 8.35 (s, 1H), 7.86 – 7.76 (m, 2H), 7.44 – 7.34 (m, 3H), 7.30 (t, *J* = 7.8 Hz, 2H), 7.21 – 6.96 (m, 3H). **^{13}C NMR** (101 MHz, $CDCl_3$) δ 160.5, 152.2, 136.3, 131.5, 129.3, 128.9, 128.9, 126.0, 121.0. Data was consistent with literature precedent.[13]

Synthesis of **1n**

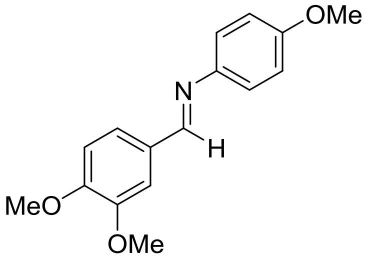

The above compound was prepared according to General Procedure **A** using veratraldehyde (1.66 g, 10 mmol) and *p*-anisidine (1.23 g, 10 mmol). The compound was used crude as >95% purity was confirmed *via* ^{1}H NMR, pale grey solid, 91% (2.46 g). **mp** (from CH_2Cl_2): 120-122 °C. **FT-IR** (thin film): ν_{max} (cm^{-1}) = 2981, 1621, 1602, 1578, 1509. **^{1}H NMR** (400 MHz, $CDCl_3$) δ 8.38 (s, 1H), 7.61 (d, *J* = 1.9 Hz, 1H), 7.34 – 7.23 (m, 1H), 7.24–7.12 (m, 2H), 6.92 (dd, *J* = 8.6, 1.4 Hz, 3H), 3.99 (s, 3H), 3.94 (s, 3H), 3.83 (s, 3H). **^{13}C NMR** (101 MHz, $CDCl_3$) δ 158.2, 158.1, 151.9, 149.6, 145.3, 129.9, 124.2, 122.2, 114.5, 110.6, 108.9, 56.1, 55.6. **HRMS** (ESI): m/z calculated for $C_{16}H_{18}O_3N_1$ requires 272.1281 for $[M+H]^+$, found 372.1279.

Synthesis of **1o**

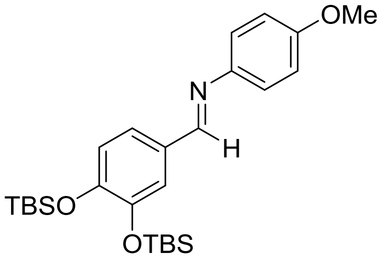

The above compound was prepared according to General Procedure **A** using 3,4-bis((tert-butyldimethylsilyl)oxy)benzaldehyde (1.83 g, 10 mmol) and *p*-anisidine (1.23 g, 10 mmol). The compound was used crude as >95% purity was confirmed *via* ^{1}H NMR, pale orange solid, 77% (3.62 g). **FTIR** (thin film): ν_{max} (cm^{-1}) = 2955, 2931, 2895, 2858, 1625, 1602, 1505. **^{1}H NMR** (400 MHz, Chloroform-*d*) δ 8.33 (s, 1H), 7.41 – 7.33 (m, 2H), 7.23 – 7.17 (m, 2H), 6.98 – 6.85 (m, 3H), 3.83 (s, 3H), 1.02 (s, 9H), 1.00 (s, 9H), 0.24 (s, 6H), 0.24 (s, 6H). **^{13}C NMR** (101 MHz, $CDCl_3$) δ 158.1, 158.0, 150.2, 147.4, 145.5, 130.6, 122.7, 122.2, 121.2, 121.0, 114.4, 55.6, 26.1, 26.1, 18.7, 18.6, -3.9, -3.9. **HRMS** (ESI): m/z calculated for $C_{26}H_{42}O_3N_1Si_2$ requires 272.2698 for $[M+H]^+$, found 472.2700.

Synthesis of **1p**

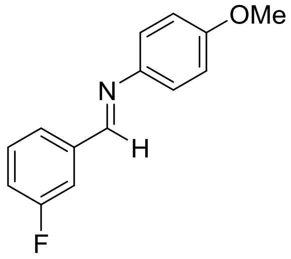

The above compound was prepared according to General Procedure **A** using 3-fluorobenzaldehyde (1.06 mL, 1.24 g, 10 mmol) and *p*-anisidine (1.23 g, 10 mmol). The compound was used crude as >95% purity was confirmed *via* ^{1}H NMR, deep grey solid, 90% (2.07 g). **^{1}H NMR** (400 MHz, $CDCl_3$) δ 8.36 (d, *J* = 1.3 Hz, 1H), 7.56 (ddd, *J* = 9.6, 2.7, 1.5 Hz, 1H), 7.52 (dt, *J* = 7.6, 1.2 Hz, 1H), 7.33 (td, *J* = 7.9, 5.6 Hz, 1H), 7.20 – 7.11 (m, 2H), 7.06 (tdd, *J* = 8.3, 2.7, 1.0 Hz, 1H), 6.89 – 6.79 (m, 2H), 3.74 (s, 3H). **^{19}F NMR** (377 MHz, $CDCl_3$) δ -112.66 (td, *J* = 8.9, 5.6 Hz). **^{13}C NMR** (101 MHz, $CDCl_3$) δ 163.2 (d, *J* = 246.4 Hz), 158.7, 156.8 (d, *J* = 3.1 Hz), 144.4, 138.9 (d, *J* = 7.2 Hz), 130.4 (d, *J* = 8.5 Hz), 124.9 (d, *J* = 3.0 Hz), 122.4, 118.0 (d, *J* = 21.5 Hz), 114.6 (d, *J* = 22.4 Hz), 114.6, 55.6. Data was consistent with literature precedent.[6]

Synthesis of **1q**

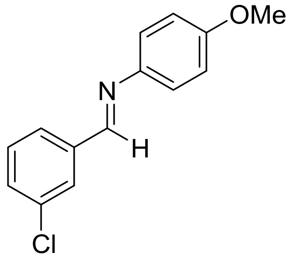

The above compound was prepared according to General Procedure **A** using 3-chlorobenzaldehyde (1.14 mL, 1.41 g, 10 mmol) and *p*-anisidine (1.23 g, 10 mmol). The compound was used crude as >95% purity was confirmed *via* ^{1}H NMR, pale off-white solid, 96% (2.36 g). **^{1}H NMR** (400 MHz, $CDCl_3$) δ 8.33 (s, 1H), 7.83 (t, *J* = 1.8 Hz, 1H), 7.63 (dt, *J* = 7.3, 1.5 Hz, 1H), 7.42 – 7.24 (m, 2H), 7.21 – 7.03 (m, 2H), 6.85 (d, *J* = 8.9 Hz, 2H), 3.74 (s, 3H). **^{13}C NMR** (101 MHz, $CDCl_3$) δ 158.7, 156.5, 144.3, 138.4, 135.0, 131.0, 130.1, 128.2, 127.0, 122.4, 114.6, 55.6. Data was consistent with literature precedent.[14]

Synthesis of **1r**

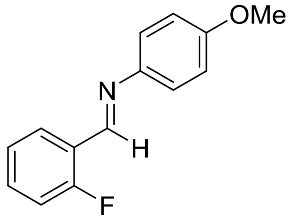

The above compound was prepared according to General Procedure **A** using 2-fluorobenzaldehyde (1.05 mL, 1.24 g, 10 mmol) and *p*-anisidine (1.23 g, 10 mmol). The compound was used crude as >95% purity was confirmed *via* ^{1}H NMR, deep grey solid, 91% (2.08 g). **^{1}H NMR** (400 MHz, $CDCl_3$) δ 8.71 (s, 1H), 8.09 (td, *J* = 7.5, 1.8 Hz, 1H), 7.34 (dddd, *J* = 8.4, 7.3, 5.3, 1.9 Hz, 1H), 7.22 – 7.11 (m, 3H), 7.03 (ddd, *J* = 10.7, 8.3, 1.1 Hz, 1H), 6.90 – 6.82 (m, 2H), 3.74 (s, 3H). **^{19}F NMR** (377 MHz, $CDCl_3$) δ -120.89 – -122.19 (m). **^{13}C NMR** (101 MHz, $CDCl_3$) δ 162.8 (d, *J* = 253.3 Hz), 158.7, 151.4 (d, *J* = 4.9 Hz), 144.9, 132.7 (d, *J* = 8.7 Hz), 127.8 (d, *J* = 2.5 Hz), 124.6 (d, *J* = 3.3 Hz), 124.3 (d, *J* = 8.8 Hz), 122.5, 115.9 (d, *J* = 21.3 Hz), 114.5, 55.6. Data was consistent with literature precedent.[15]

Synthesis of **1s**

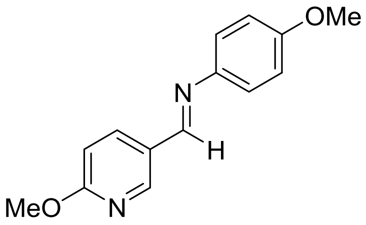

The above compound was prepared according to General Procedure **A** using 6-methoxy-3-pyridinecarboxaldehyde (686 mg, 5 mmol) and *p*-anisidine (616 mg, 5 mmol). The compound was used crude as >95% purity was confirmed *via* ^{1}H NMR, orange solid, 79% (960 mg). **mp** (from CH_2Cl_2) = 81 – 83 °C. **FTIR** (thin film): ν_{max} (cm^{-1}) = 3016, 2955, 2908, 2882, 2839, 1621, 1608, 1577, 1568, 1507. **^{1}H NMR** (400 MHz, $CDCl_3$) δ 8.46 (d, *J* = 2.4 Hz, 1H), 8.43 (s, 1H), 8.25 (dd, *J* = 8.7, 2.3 Hz, 1H), 7.22 (d, *J* = 8.8 Hz, 2H), 6.93 (d, *J* = 8.8 Hz, 2H), 6.83 (d, *J* = 8.7 Hz, 1H), 4.00 (s, 3H), 3.83 (s, 3H). **^{13}C NMR** (101 MHz, $CDCl_3$) δ 166.0, 158.4, 155.1, 149.7, 145.0, 137.0, 126.5, 122.3, 114.6, 111.8, 55.6, 54.0.

Synthesis of **1t**

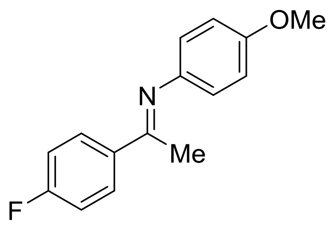

To a 100 mL round bottomed flask was charged *p*-anisidine (2.46 g, 20 mmol), 4Å molecular sieves (20 g), and 4-fluoroacetophenone (2.43 mL, 2.76 g, 20 mmol). The flask was equipped with a reflux condenser and the system purged with nitrogen for 5 minutes. After this time anhydrous toluene (30 mL) was added *via* syringe and the reaction mixture heated to 80 °C overnight. After this time, the reaction mixture was allowed to cool to room temperature and was filtered through a pad of celite, eluting with EtOAc. The filtrate was concentrated *in vacuo* and the crude residue was purified *via* recrystallization from EtOH to give pure ketimine, 41% (2.05 g). ^{1}**H NMR** (400 MHz, Chloroform-*d*) δ 8.05 – 7.85 (m, 2H), 7.21 – 7.02 (m, 2H), 7.01 – 6.86 (m, 2H), 6.86 – 6.71 (m, 2H), 3.84 (s, 3H), 2.26 (s, 3H). ^{19}**F NMR** (377 MHz, $CDCl_3$) δ -110.67 (tt, *J* = 8.5, 5.5 Hz). ^{13}**C NMR** (101 MHz, $CDCl_3$) δ 164.5, 164.3 (d, *J* = 250.3 Hz), 156.1, 144.7, 136.0 (d, *J* = 3.1 Hz), 129.3 (d, *J* = 8.7 Hz), 120.9, 115.3 (d, *J* = 21.5 Hz), 114.4, 55.6, 17.3. Data was consistent with literature precedent.[16]

Synthesis of *N*-(4-fluorobenzyl)-4-methoxyaniline

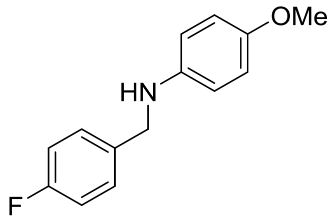

To a vial equipped with a stirrer bar was added **1a** (228 mg, 1 mmol) and MeOH (4 mL). The vial was cooled to 0 °C and sodium borohydride (42 mg, 1.1 mmol) was added portionwise. The vial was allowed to return to room temperature and stir for 2 hours. The reaction mixture was diluted in EtOAc (50 mL) and brine (50 mL) and the organic phase separated. The aqueous phase was re-extracted with EtOAc (2 x 50 mL) and the combined organics were dried over $MgSO_4$ and concentrated *in vacuo* to give pure title compound, 85% (196 mg). **^{1}H NMR** (400 MHz, $CDCl_3$) δ 7.37 – 7.30 (m, 2H), 7.03 (t, *J* = 8.7 Hz, 2H), 6.86 – 6.62 (m, 2H), 6.67 – 6.50 (m, 2H), 4.26 (s, 2H), 3.75 (s, 3H). **^{19}F NMR** (377 MHz, $CDCl_3$) δ -115.71 (tt, *J* = 9.1, 5.1 Hz). **^{13}C NMR** (101 MHz, $CDCl_3$) δ 162.1 (d, *J* = 245.2 Hz), 152.4, 142.3, 135.5 (d, *J* = 3.2 Hz), 129.2 (d, *J* = 8.0 Hz), 115.5 (d, *J* = 21.4 Hz), 115.0, 114.3, 55.9, 48.6. Data was consistent with literature precedent.[17]

Synthesis of **HE2**

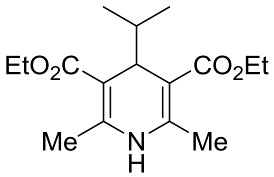

To an oven-dried carousel tube was charged ethyl acetoacetate (1.27 mL, 1.30 g, 10 mmol), isobutyraldehyde (0.45 mL, 360 mg, 5 mmol), ammonium acetate (385 mg, 5 mmol) and ethanol (6 mL). The mixture was then heated to reflux overnight before being allowed to return to room temperature and concentrated *in vacuo*. The crude residue was diluted in CH_2Cl_2 (50 mL) and brine (50 mL) and the organic phase was extracted. The aqueous phase was re-extracted with CH_2Cl_2 (2 x 50 mL). The combined organics were dried over $MgSO_4$ and concentrated *in vacuo*. The crude residue was then purified *via* silica gel column chromatography (EtOAc : Pentane 10:90 - 20:80 v:v) to give the desired compound as a pale orange solid, 55% (810 mg). **^{1}H NMR** (400 MHz, $CDCl_3$) δ 5.96 (s, 1H), 4.28 – 4.04 (m, 4H), 3.89 (d, *J* = 5.4 Hz, 1H), 2.27 (s, 6H), 1.26 (t, *J* = 7.1 Hz, 6H), 0.71 (d, *J* = 6.9 Hz, 6H). **^{13}C NMR** (101 MHz, $CDCl_3$) δ 168.9, 144.8, 101.7, 59.7, 38.9, 35.6, 19.4, 18.6, 14.5. Data was consistent with literature precedent.[18]

Synthesis of **HE3**

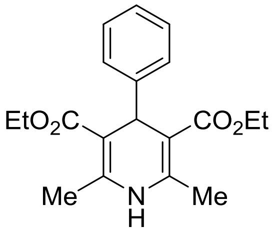

A flask equipped with a reflux condenser was charged with ethyl 3-oxobutanoate (11.5 ml, 60 mmol), benzaldehyde (5.0 mL, 30 mmol) and ethanol (anhydrous, 50 mL). The mixture was heated to reflux for 60 min and ammonium acetate (1.92 g, 30 mmol) was added and reflux maintained. After 3 days the reaction was cooled down, diluted with brine (100 mL) and extracted with ethyl acetate (3 x 50mL). The combined organic layers were dried and the solvent removed. The resulting heterogeneous mixture was triturated with pentane and purified *via* silica gel column chromatography (EtOAc:Pentane 5:95-10:90 v:v) to give the desired product as a white crystalline solid (6.22g, 39%). **^{1}H NMR** (400 MHz, $CDCl_3$) δ 7.33 – 7.27 (m, 2H), 7.22 (t, *J* = 7.5 Hz, 2H), 7.18 – 7.05 (m, 1H), 5.94 (s, 1H), 5.01 (s, 1H), 4.21 – 3.88 (m, 4H), 2.33 (s, 6H), 1.24 (t, *J* = 7.1 Hz, 6H). **^{13}C NMR** (101 MHz, $CDCl_3$) δ 167.8, 147.9, 144.1, 128.1, 127.9, 126.2, 104.1, 59.8, 39.7, 19.6, 14.3. Data was consistent with literature precedent.[19]

Synthesis of **HE4**

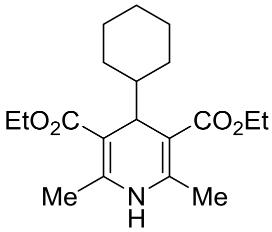

To a 250 mL round bottomed flask was charged ethyl acetoacetate (5.09 mL, 5.20 g, 40 mmol), cyclohexanecarboxaldehyde (2.42 mL, 2.24 g, 20 mmol), ammonium acetate (1.54 g, 20 mmol) and ethanol (40 mL). The mixture was then heated to reflux overnight before being allowed to return to room temperature and concentrated *in vacuo*. The crude residue was diluted in CH_2Cl_2 (150 mL) and brine (150 mL) and the organic phase was extracted. The aqueous phase was re-extracted with CH_2Cl_2 (2 x 150 mL). The combined organics were dried over $MgSO_4$ and concentrated *in vacuo*. The crude residue was then purified *via* silica gel column chromatography (EtOAc:Pentane - 20:80 v:v) to give a powdery white solid, 40% (2.7 g). **^{1}H NMR** (400 MHz, $CDCl_3$) δ 5.60 (s, 1H), 4.30–4.07 (m, 4H), 3.91 (d, *J* = 5.7 Hz, 1H), 2.29 (s, 6H), 1.65 (d, *J* = 7.5 Hz, 2H), 1.60–1.49 (m, 3H), 1.29 (t, *J* = 7.1 Hz, 6H), 1.21 (dt, *J* = 5.9, 3.0 Hz, 1H), 1.07 (tt, *J* = 8.9, 2.5 Hz, 3H), 0.98–0.83 (m, 2H). **^{13}C NMR** (101 MHz, $CDCl_3$) δ 168.9, 144.5, 102.1, 59.7, 45.9, 38.5, 29.0, 26.9, 26.8, 19.6, 14.5. Data was consistent with literature precedent.[20]

Synthesis of **HE5**

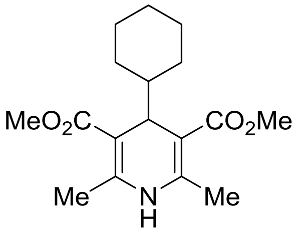

To an oven-dried carousel tube was charged methyl acetoacetate (1.08 mL, 1.16 g, 10 mmol), cyclohexanecarboxaldehyde (0.60 mL, 560 mg, 5 mmol), ammonium acetate (385 mg, 5 mmol) and ethanol (6 mL). The mixture was then heated to reflux overnight before being allowed to return to room temperature and concentrated *in vacuo*. The crude residue was diluted in CH_2Cl_2 (50 mL) and brine (50 mL) and the organic phase was extracted. The aqueous phase was re-extracted with CH_2Cl_2 (2 x 50 mL). The combined organics were dried over $MgSO_4$ and concentrated *in vacuo*. The crude residue was then purified *via* silica gel column chromatography (EtOAc : Pentane 10:90 - 20:80 v:v) to give the desired compound as a powdery white solid, 63% (970 mg). **^{1}H NMR** (400 MHz, $CDCl_3$) δ 5.99 (s, 1H), 3.86 (d, *J* = 5.8 Hz, 1H), 3.68 (s, 6H), 2.28 (s, 6H), 1.62 (dq, *J* = 6.6, 3.2 Hz, 2H), 1.57 – 1.45 (m, 3H), 1.17 (tdt, *J* = 11.9, 6.1, 3.2 Hz, 1H), 1.10 – 0.95 (m, 3H), 0.86 (tt, *J* = 12.5, 5.3 Hz, 2H). **^{13}C NMR** (101 MHz, $CDCl_3$) δ 169.3, 145.1, 101.5, 51.0, 45.7, 38.4, 28.7, 26.7, 26.7, 19.4. Data was consistent with literature precedent.[21]

Synthesis of **HE6**

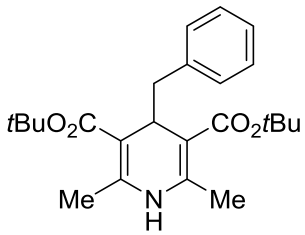

A flask equipped with a reflux condenser was charged with *tert*-butyl 3-oxobutanoate (10.0 ml, 60 mmol), 2-phenylacetaldehyde (3.6mL, 30 mmol) and ethanol (anhydrous, 60mL). The mixture was heated to reflux for 60 min and ammonia (30% in water, 20mL) was added and temperature retained at reflux. After 3 days the reaction was cooled down, quenched with HCl (aq. sol., 3M, 100mL) and extracted with CH_2Cl_2 (3 x 100 mL). The combined organic layers were dried and the solvent removed. The resulting crude residue was purified *via* silica gel column chromatography (EtOAc:Pentane - 10:90 v:v) and then triturated with pentane to give the desired product as a white crystalline solid (2.5g, 24%). This compound was subsequently stored under N_2 and at -20 °C to avoid spontaneous oxidation. **^{1}H NMR** (400 MHz, Chloroform-*d*) δ 7.23 – 7.10 (m, 3H), 7.06 – 6.98 (m, 2H), 5.19 (s, 1H), 4.14 (t, *J* = 5.7 Hz, 1H), 2.54 (d, *J* = 5.7 Hz, 2H), 2.16 (s, 6H), 1.42 (s, 18H). **^{13}C NMR** (101 MHz, $CDCl_3$) δ 167.2, 144.5, 139.6, 130.3, 127.6, 125.7, 103.4, 79.4, 42.0, 36.0, 28.4, 28.4, 28.3, 19.4. Data was consistent with literature precedent.[22]

Synthesis of 2-(2,4-difluorophenyl)-5-(trifluoromethyl)pyridine

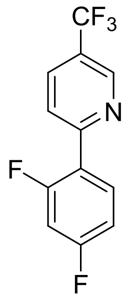

To a three-necked 250 mL round bottomed flask was charged with 2,4-difluorophenylboronic acid (5.68 g, 36 mmol), 2-bromo-5-trifluoromethylpyridine (6.78 g, 30 mmol), potassium carbonate (12.4 g, 90 mmol), palladium acetate (202 mg, 0.9 mmol) and triphenylphosphine (472 mg, 1.8 mmol). The flask was equipped with a condenser then evacuated and refilled with N_2 three times. Following this, toluene (40 mL), water (40 mL) and ethanol (8 mL) were added *via* septum. The flask was heated to reflux for 16 h. After this time, the flask was cooled to room temperature and quenched with water (100 mL). The organic phase was separated and then the aqueous phase re-extracted with Et_2O (3 x 200 mL). The combined organics were washed with brine (3 x 200 mL) and then dried over $MgSO_4$ and concentrated *in vacuo*. The crude residue was the then purified *via* silica gel column chromatography (EtOAc:Pentane 1:99 – 4:96 v:v) to give the title compound as a white solid, 85% (6.62 g). **^{1}H NMR** (400 MHz, $CDCl_3$) δ 8.85 (dd, *J* = 2.4, 1.2 Hz, 1H), 7.99 (td, *J* = 8.9, 6.6 Hz, 1H), 7.87 (dd, *J* = 8.3, 2.4 Hz, 1H), 7.82 – 7.76 (m, 1H), 6.98 – 6.88 (m, 1H), 6.82 (ddd, *J* = 11.3, 8.7, 2.5 Hz, 1H). **^{19}F NMR** (377 MHz, $CDCl_3$) δ -62.49, -107.28 (t, *J* = 8.0 Hz), -112.04 (d, *J* = 10.0 Hz). **^{13}C NMR** (101 MHz, $CDCl_3$) δ 164.0 (dd, *J* = 252.8, 12.5 Hz), 161.1 (dd, *J* = 253.6, 12.0 Hz), 155.9, 146.7 (q, *J* = 4.2 Hz), 133.8 (q, *J* = 3.5 Hz), 132.6 (dd, *J* = 10.0, 4.2 Hz), 125.3 (q, *J* = 33.2 Hz), 123.7 (q, *J* = 272.3 Hz), 123.7 (d, *J* = 11.1 Hz), 122.5 (dd, *J* = 11.3, 3.8 Hz), 112.4 (dd, *J* = 21.1, 3.6 Hz), 106.5 – 101.0 (app t). Data was consistent with literature precedent.[23]

Synthesis of [Ir(dF(CF_3)ppy)$_2$Cl]$_2$

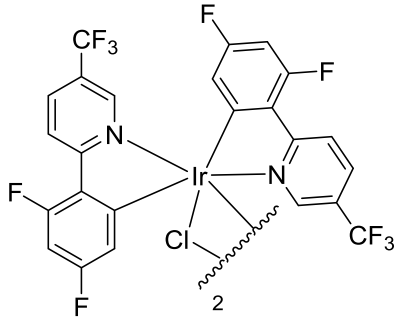

To a three-necked 100 mL round bottomed flask was charged iridium(III) chloride hydrate (448 mg, 1.5 mmol) and 2-(2,4-difluorophenyl)-5-(trifluoromethyl)pyridine (856 mg, 3.3 mmol). The flask was equipped with a condenser, then evacuated and refilled with nitrogen three times. Rigorously degassed 2-ethoxyehtanol (18 mL) and water (6 mL) were added *via* syringe. The reaction mixture was heated 150 °C for 16 h. After this time the reaction mixture was allowed to return to room temperature and the bright yellow precipitate formed was filtered under a blanket of N_2, washing with water (150 mL) and then hexane (60 mL), to give title compound after further removal of water *via* high vacuum, 86% (960 mg). **^{1}H NMR** (400 MHz, $CDCl_3$) δ 9.51 (d, *J* = 2.1 Hz, 1H), 8.46 (dd, *J* = 8.7, 3.0 Hz, 1H), 8.05 (dd, *J* = 8.7, 2.3 Hz, 1H), 6.43 (ddd, *J* = 12.5, 8.8, 2.3 Hz, 1H), 5.07 (dd, *J* = 8.8, 2.3 Hz, 1H). **^{19}F NMR** (377 MHz, $CDCl_3$) δ -62.36 (12 F), -103.41 – -103.72 (m, 4F), -106.97 – -107.91 (m, 4F). Data was consistent with literature precedent.[23]

Synthesis of (Ir[dF(CF_3)ppy)$_2$](dtbbpy))PF_6 - **[Ir]**

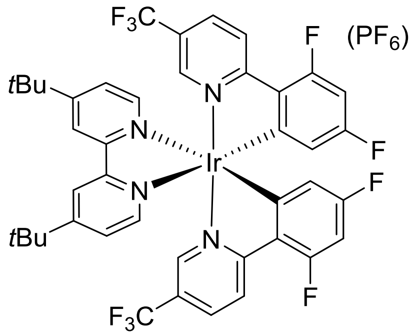

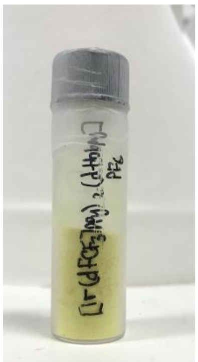

Procedure adapted from a previous report.[24] To a three-necked 250 mL round bottomed flask was charged [Ir(dF(CF_3)ppy)$_2$Cl]$_2$ (960 mg, 0.65 mmol) and 4,4'-di-*tert*-butyl-2,2'-dipyridyl (429 mg, 1.6 mmol). The flask was equipped with a reflux condenser, then evacuated and refilled three times with nitrogen. Rigorously degassed ethylene glycol (44 mL) was then added *via* syringe. The reaction mixture was then heated to 150 °C for 16 h. After this time the flask was allowed to return to room temperature. The mixture was diluted in water (300 mL) and hexane (300 mL). The aqueous phase was then separated and then re-extracted with hexane (2 x 300 mL). The aqueous phase was then decanted into a 500 mL conical flask and equipped with a stirrer bar. The flask was heated at 80 °C for 1 hour to remove residual hexane. The flask was allowed to return to room temperature, and an aqueous solution of potassium hexafluorophosphate (7 g in 70 mL water) was added with stirring, and a vibrant yellow precipitate was formed. The mixture was then allowed to stand at 5 °C for 1 hour, before the precipitate was collected *via* vacuum filtration washing with water (150 mL) and hexane (100 mL), The collected powdery solid was then subjected to further water removal *via* high vacuum, to give the title compound, 63% (915 mg). **^{1}H NMR** (400 MHz, $CDCl_3$) δ 8.88 (d, *J* = 2.1 Hz, 2H), 8.47 (dd, *J* = 8.7, 3.1 Hz, 2H), 8.04 (dd, *J* = 8.8, 2.1 Hz, 2H), 7.86 (d, *J* = 5.9 Hz, 2H), 7.59 (dd, *J* = 5.9, 1.9 Hz, 2H), 7.41 (s, 2H), 6.64 (ddd, *J* = 11.6, 8.9, 2.3 Hz, 2H), 5.63 (dd, *J* = 8.0, 2.4 Hz, 2H), 1.50 (s, 18H). **^{19}F NMR** (377 MHz, $CDCl_3$) δ -62.99 (6F), -72.24 (3F), -74.13 (3F), -101.81 (dt, *J* = 12.4, 8.4 Hz, 2F), -105.92 (td, *J* = 12.4, 3.4 Hz, 2F). NMR Spectra matched those from commercial sources.

6. Synthesis of Cyclised Products

General Procedure **B** for the Photoredox Catalysed Retropolar Povarov Reaction

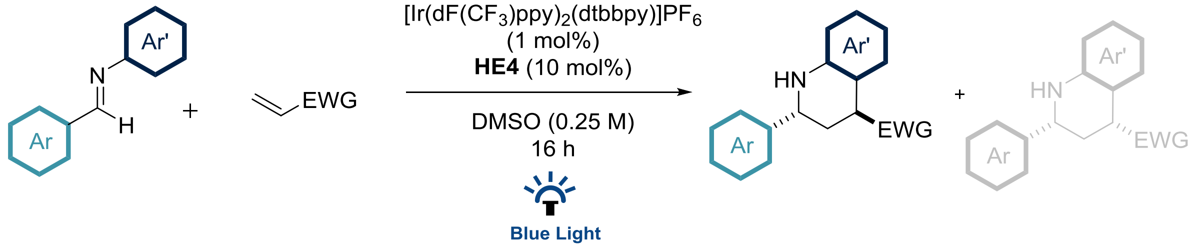

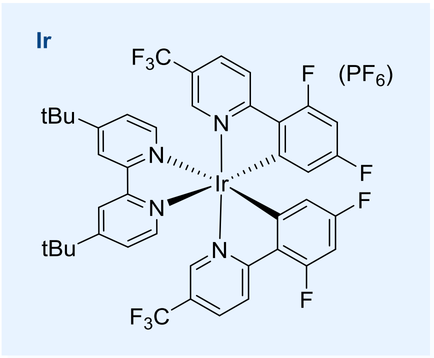

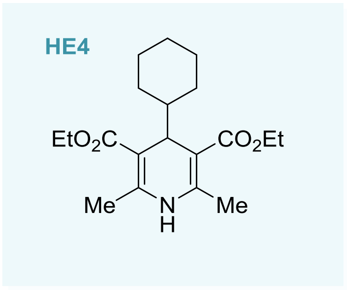

To a 1.6 mL glass vial equipped with a microstirrer, was charged relevant imine (0.25 mmol), relevant coupling partner (0.5-1.25 mmol), diethyl 4-cyclohexyl-2,6-dimethyl-1,4-dihydropyridine-3,5-dicarboxylate (**HE4**, 8.4 mg, 0.025 mmol), and $[Ir(dF(CF_3)ppy)_2(dtbbpy)]PF_6$ (2.8 mg, 0.0025 mmol). To the vial was added anhydrous DMSO (1 mL). Nitrogen was then bubbled through the stirred solution for 10 minutes. The vial was then sealed and then irradiated with blue light while stirring using a photoreactor set-up for 16-64 h. After this time an aliquot of the reaction mixture was dissolved in $CDCl_3$ and ^{1}H NMR spectroscopy was used to determine the crude diastereomeric ratio. The reaction mixture was then diluted in EtOAc (20 mL), H_2O (10 mL) and brine (10 mL). The organic phase was extracted and the aqueous phase re-extracted with EtOAc (2 x 20 mL). The combined organic phases were dried over $MgSO_4$ and concentrated *in vacuo*. The crude residue was purified *via* silica gel column chromatography (either Pentane:EtOAc, Pentane:CH_2Cl_2 or Pentane:Acetone – see individual experiment – phenyl vinyl sulfone and Hantzsch Ester often close in R_f to product) to give a diastereomeric mixture of products. Trituration with $CHCl_3$:Pentane was utilised to improve diastereomeric ratio for analysis when necessary and/or possible.

Synthesis of **3a**

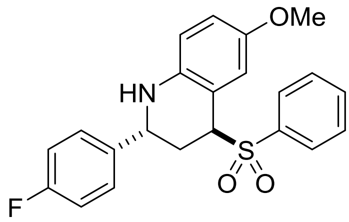

The above compound was synthesized according to General Procedure **B** using **1a** (57 mg, 0.25 mmol) and phenyl vinyl sulfone (210 mg, 1.25 mmol) and irradiated with blue light for 16 h. Crude dr: 10:1 Silica gel column chromatography (EtOAc:Pentane 5:95 – 40:60 v:v) to give **3a** as a white solid, 90% (89 mg, isolated as 17:1 dr). **mp** (from EtOAc:Pentane): 170-174 °C. **FT-IR** (thin film): ν_{max} (cm^{-1}) = 3373, 2930, 1506, 1479, 1303 S=O) ^{1}**H NMR** (400 MHz, $CDCl_3$) δ 7.86 – 7.78 (m, 2H), 7.75 – 7.64 (m, 1H), 7.57 (dd, *J* = 8.5, 7.1 Hz, 2H), 7.36 (dd, *J* = 8.6, 5.4 Hz, 2H), 7.04 (t, *J* = 8.6 Hz, 2H), 6.78 (dd, *J* = 8.8, 2.9 Hz, 1H), 6.55 (d, *J* = 8.8 Hz, 1H), 6.34 (d, *J* = 2.9 Hz, 1H), 4.73 (dd, *J* = 12.5, 3.1 Hz, 1H), 4.29 (dd, *J* = 5.9, 1.5 Hz, 1H), 3.99 (s, 1H), 3.57 (s, 3H), 2.61 (ddd, *J* = 14.6, 3.1, 1.5 Hz, 1H), 2.04 (ddd, *J* = 14.7, 12.5, 5.9 Hz, 1H). ^{19}**F NMR** (377 MHz, $CDCl_3$) δ -114.29 (ddd, *J* = 13.7, 8.3, 5.2 Hz) – minor diastereomer δ -113.89 (ddd, *J* = 13.6, 8.9, 5.3 Hz). ^{13}**C NMR** (101 MHz, $CDCl_3$) δ 162.5 (d, *J* = 246.4 Hz), 151.3, 140.4, 139.0 (d, *J* = 3.1 Hz), 137.9, 134.3 (d, *J* = 85.1 Hz), 134.0, 129.4 (d, *J* = 24.4 Hz), 128.4 (d, *J* = 8.0 Hz), 118.2, 116.4, 115.8, 115.7, 115.6, 109.9, 63.3, 55.7, 51.2, 31.0. **HRMS** (ESI): m/z calculated for $C_{22}H_{21}O_3N_1F_1S_1$ requires 398.1121 for $[M+H]^+$, found 398.1220.

Synthesis of **3b**

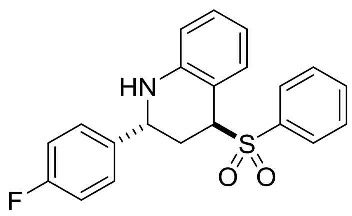

The above compound was synthesized according to General Procedure **B** using **1b** (50 mg, 0.25 mmol) and phenyl vinyl sulfone (84 mg, 0.5 mmol) and irradiated with blue light for 16 h. Crude dr: >20:1. Silica gel column chromatography (CH_2Cl_2:Pentane 10:90 – 50:50 v:v) to give **3b** as a white solid, 74% (68 mg – isolated as >20:1 dr). **mp** (from CH_2Cl_2:Pentane): 192-196 °C. **FT-IR** (thin film): ν_{max} (cm^{-1}) =3378, 2980, 1493, 1382, 1305. **^{1}H NMR** (400 MHz, DMSO-d_6) δ 7.95 – 7.87 (m, 2H), 7.82 – 7.72 (m, 1H), 7.68 (dd, *J* = 8.3, 7.0 Hz, 2H), 7.40 (dd, *J* = 8.7, 5.6 Hz, 2H), 7.19 (t, *J* = 8.8 Hz, 1H), 7.06 (ddd, *J* = 8.4, 7.2, 1.6 Hz, 1H), 6.90 (dd, *J* = 7.8, 1.5 Hz, 1H), 6.69 (dd, *J* = 8.3, 1.3 Hz, 1H), 6.56 – 6.42 (m, 2H), 4.68 (dd, *J* = 5.7, 1.6 Hz, 1H), 4.63 (dd, *J* = 12.2, 3.4 Hz, 1H), 2.07 – 1.97 (m, 1H), 1.90 (ddd, *J* = 14.6, 12.2, 5.7 Hz, 1H). **^{19}F NMR** (377 MHz, DMSO-d_6) δ -110.16 (td, *J* = 9.1, 5.7 Hz) – minor diastereomer δ -110.01 – -110.10 (m). **^{13}C NMR** (101 MHz, $CDCl_3$) δ 161.6 (d, *J* = 243.2 Hz), 146.8, 139.4 (d, *J* = 3.1 Hz), 137.4, 134.1, 132.0, 129.4, 129.4, 128.9, 128.6 (d, *J* = 8.1 Hz), 115.3 (d, *J* = 24.3 Hz), 115.2, 114.6, 108.1. **HRMS** (ESI): m/z calculated for $C_{21}H_{19}O_2N_1F_1S_1$ requires 368.1115 for $[M+H]^+$, found 368.1113.

Synthesis of **3c**

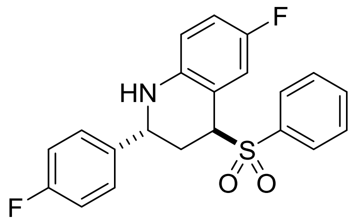

The above compound was synthesized according to General Procedure **B** using **1c** (54 mg, 0.25 mmol) and phenyl vinyl sulfone (84 mg, 0.5 mmol) and irradiated with blue light for 16 h. Crude dr: 8:1. Silica gel column chromatography (CH_2Cl_2:Pentane 10:90 – 50:50 v:v) to give **3c** as a white solid, 60% (58 mg – isolated as 9:1 dr). **mp** (from CH_2Cl_2:Pentane): 202-206 °C. **FT-IR** (thin film): ν_{max} (cm^{-1}) = 3383 (NH), 2980 (CH), 1505, 1383, 1305 (SO). **^{1}H NMR** (400 MHz, DMSO-d_6) δ 7.93 (d, *J* = 7.1 Hz, 2H), 7.83 – 7.76 (m, 1H), 7.71 (dd, *J* = 8.4, 7.0 Hz, 2H), 7.41 (dd, *J* = 8.6, 5.6 Hz, 2H), 7.20 (t, *J* = 8.8 Hz, 2H), 6.99 (td, *J* = 8.6, 3.0 Hz, 1H), 6.72 (td, *J* = 9.6, 8.9, 4.0 Hz, 2H), 6.47 (s, 1H), 4.82 – 4.70 (m, 1H), 4.57 (dd, *J* = 12.1, 3.4 Hz, 1H), 2.03 – 1.96 (m, 1H), 1.90 (ddd, *J* = 14.7, 12.1, 5.8 Hz, 1H). **^{19}F NMR** (377 MHz, DMSO-d_6) δ -114.83 (tt, *J* = 9.9, 5.5 Hz), -129.84 (td, *J* = 9.0, 5.0 Hz) – minor diastereomer δ -114.71 (ddd, *J* = 14.4, 9.6, 5.6 Hz), -126.90 (td, *J* = 9.5, 9.0, 5.2 Hz). **^{13}C NMR** (101 MHz, DMSO-d_6) δ 161.6 (d, *J* = 243.3 Hz), 153.3 (d, *J* = 230.9 Hz), 143.6, 139.2, 137.1, 134.3, 129.5 (d, *J* = 38.3 Hz), 128.7 (d, *J* = 8.0 Hz), 117.5 (d, *J* = 22.5 Hz), 116.7 (d, *J* = 22.4 Hz), 115.4, 115.2, 115.5, 108.6 (d, *J* = 7.2 Hz), 60.8, 49.6, 30.2. **HRMS** (ESI): m/z calculated for $C_{21}H_{18}O_2N_1F_2S_1$ requires 386.1021 for $[M+H]^+$, found 386.1026.

Synthesis of **3d**

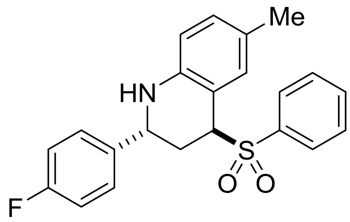

The above compound was synthesized according to General Procedure **B** using **1d** (53 mg, 0.25 mmol) and phenyl vinyl sulfone (84 mg, 0.5 mmol) and irradiated with blue light for 40 h. Crude dr: >20:1. Silica gel column chromatography (CH_2Cl_2:Pentane 10:90 – 50:50 v:v) to give **3d** as a white solid, 73% (68 mg – isolated as >20:1 dr). **mp** (from CH_2Cl_2): 208-212 °C). **FT-IR** (thin film): ν_{max} (cm^{-1}) =3376, 2912, 1617, 107, 1447, 1303. **^{1}H NMR** (400 MHz, DMSO-d_6:$CDCl_3$ 1:1) δ 7.81 (dd, *J* = 7.6, 2.1 Hz, 2H), 7.70 (dd, *J* = 8.4, 6.2 Hz, 1H), 7.60 (td, *J* = 7.8, 2.1 Hz, 2H), 7.34 (ddd, *J* = 8.3, 5.3, 2.2 Hz, 2H), 7.03 (td, *J* = 8.8, 2.3 Hz, 2H), 6.89 – 6.79 (m, 1H), 6.62 – 6.51 (m, 2H), 4.64 (dd, *J* = 12.4, 2.7 Hz, 1H), 4.38 (d, *J* = 5.6 Hz, 1H), 3.46 (s, 1H), 2.24 – 2.10 (m, 1H), 2.05 (d, *J* = 2.1 Hz, 3H), 1.87 (tdd, *J* = 14.5, 5.9, 2.2 Hz, 1H). **^{19}F NMR** (377 MHz, DMSO-d_6) δ -114.81 (q, *J* = 7.5 Hz) – minor diastereomer δ -112.61 (q, *J* = 7.4, 6.1 Hz). **^{13}C NMR** (101 MHz, DMSO-d_6) δ 161.4 (d, *J* = 244.6 Hz), 144.2, 139.1 (d, *J* = 3.0 Hz), 137.2, 133.5, 131.8, 130.0, 128.8 (d, *J* = 11.7 Hz), 128.1 (d, *J* = 8.0 Hz), 123.7, 115.0, 114.7 (d, *J* = 15.8 Hz), 107.8, 61.7, 49.8, 30.5, 19.8. **HRMS** (ESI): m/z calculated for $C_{22}H_{21}O_2N_1F_1S_1$ requires 382.1272 for $[M+H]^+$, found 382.1276.

Synthesis of **3e**

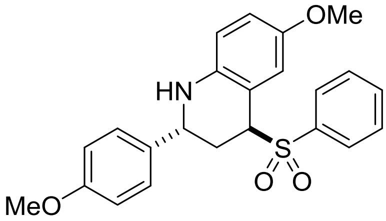

The above compound was synthesized according to General Procedure **B** using **1e** (60 mg, 0.25 mmol) and phenyl vinyl sulfone (84 mg, 0.5 mmol) and irradiated with blue light for 40 h. Crude dr: 9:1. Silica gel column chromatography (CH_2Cl_2:Pentane 10:90 – 50:50 v:v) to give **3e** as an off-white solid, 92% (94 mg – isolated as 8:1 dr). The product was then triturated with $CHCl_3$:Pentane to obtain improve diastereoselectivity for analysis. **mp** (from CH_2Cl_2:Pentane): 201-204 °C. **FT-IR** (thin film): ν_{max} (cm^{-1}) = 3375, 2980, 1506, 1383. **^{1}H NMR** (400 MHz, DMSO-d_6) δ 7.89 (d, *J* = 7.6 Hz, 2H), 7.78 (d, *J* = 7.4 Hz, 1H), 7.69 (t, *J* = 7.6 Hz, 2H), 7.27 (d, *J* = 8.1 Hz, 2H), 6.92 (d, *J* = 8.1 Hz, 2H), 6.74 (dd, *J* = 8.8, 2.9 Hz, 1H), 6.64 (d, *J* = 8.9 Hz, 1H), 6.41 (d, *J* = 3.0 Hz, 1H), 6.04 (s, 1H), 4.67 (d, *J* = 5.6 Hz, 1H), 4.52 (dd, *J* = 12.3, 3.0 Hz, 1H), 3.74 (s, 3H), 3.50 (s, 3H), 3.36 (s, 1H), 2.07 (d, *J* = 14.5 Hz, 1H), 1.89 (td, *J* = 13.9, 5.8 Hz, 1H). **^{13}C NMR** (101 MHz, DMSO-d_6) δ 158.7, 149.6, 141.4, 137.6, 135.4, 134.1, 129.5, 128.9, 127.7, 117.0, 115.8, 115.7, 113.9, 108.6, 61.6, 55.2, 55.1, 49.9, 30.7. **HRMS** (ESI): m/z calculated for $C_{23}H_{24}O_4N_1S_1$ requires 410.1421 for $[M+H]^+$, found 410.1419.

Synthesis of **3f**

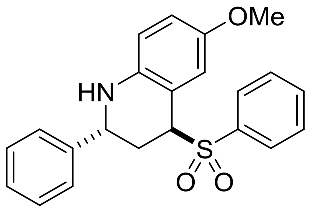

The above compound was synthesized according to General Procedure **B** using **1f** (53 mg, 0.25 mmol) and phenyl vinyl sulfone (84 mg, 0.5 mmol) and irradiated with blue light for 40 h. Crude dr: 7:1. Silica gel column chromatography (Acetone:Pentane 5:95 – 20:80 v:v) to give **3f** as a white solid, 83% (79 mg – isolated as 7:1 dr). **mp** (from Acetone:Pentane): 118-123 °C. **FT-IR** (thin film): ν_{max} (cm^{-1}) = 2980, 2889, 1585, 1505, 1474, 1462, 1447, 1382 (S=O). **^{1}H NMR** (400 MHz, $CDCl_3$) δ 7.91 – 7.79 (m, 2H), 7.70 – 7.64 (m, 1H), 7.63 – 7.52 (m, 3H), 7.42 – 7.29 (m, 4H), 6.79 (dd, *J* = 8.8, 2.9 Hz, 1H), 6.54 (d, *J* = 8.8 Hz, 1H), 6.42 (d, *J* = 2.9 Hz, 1H), 4.72 (dd, *J* = 12.5, 3.1 Hz, 1H), 4.30 (dd, *J* = 5.9, 1.5 Hz, 1H), 4.02 (d, *J* = 3.7 Hz, 1H), 3.59 (s, 2H), 2.61 (ddd, *J* = 14.7, 3.1, 1.4 Hz, 1H), 2.13 – 1.98 (m, 1H). **^{13}C NMR** (101 MHz, DMSO-d_6) δ 149.7, 143.5, 141.4, 137.5, 134.4, 134.1, 129.6, 129.5, 129.0, 128.5, 128.5, 128.0, 127.6, 126.6, 117.1, 115.8, 115.8, 108.7, 61.5, 55.2, 50.6, 30.7. **HRMS** (ESI): m/z calculated for $C_{22}H_{22}O_3N_1S_1$ requires 380.1315 for $[M+H]^+$, found 380.1311.

Synthesis of **3g**

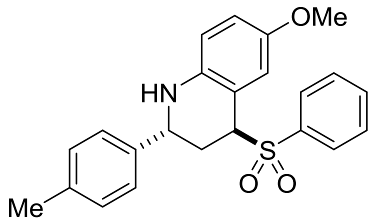

The above compound was synthesized according to General Procedure **B** using **1g** (56 mg, 0.25 mmol) and phenyl vinyl sulfone (84 mg, 0.5 mmol) and irradiated with blue light for 64 h. Crude dr: 5:1 Silica gel column chromatography (CH_2Cl_2:Pentane 20:80 – 90:10 v:v) to give **3g** as a white solid, 85% (83 mg, isolated as 5:1 dr) . The product was then triturated with $CHCl_3$:Pentane to obtain a single diastereomer for analysis. **mp** (from CH_2Cl_2:Pentane): 206-208 °C. **FT-IR** (thin film): ν_{max} (cm^{-1}) = 3375, 2980, 1506, 1382, 1303. **^{1}H NMR** (400 MHz, DMSO-d_6) δ 7.89 (d, *J* = 7.3 Hz, 2H), 7.78 (t, *J* = 7.3 Hz, 1H), 7.69 (t, *J* = 10.0 Hz,2H), 7.24 (d, *J* = 7.9 Hz, 2H), 7.16 (d, *J* = 8.0 Hz, 2H), 6.75 (dd, *J* = 8.8, 2.9 Hz, 1H), 6.66 (d, *J* = 8.9 Hz, 1H), 6.42 (d, *J* = 2.9 Hz, 1H), 6.06 (s, 1H), 4.67 (d, *J* = 5.6 Hz, 1H), 4.54 (dd, *J* = 12.3, 3.0 Hz, 1H), 3.50 (s, 3H), 2.29 (s, 3H), 2.15 – 2.04 (m, 1H), 1.88 (ddd, *J* = 14.5, 12.3, 5.9 Hz, 1H). **^{13}C NMR** (101 MHz, DMSO) δ 150.1, 141.9, 141.0, 138.1, 137.2, 134.5, 129.9, 129.5, 129.4, 127.0, 117.5, 116.3, 116.2, 109.2, 62.1, 55.7, 50.7, 21.2. **HRMS** (ESI): m/z calculated for $C_{23}H_{24}O_3N_1S_1$ requires 394.1471 for $[M+H]^+$, found 394.1474.

Synthesis of **3h**

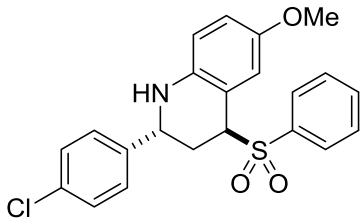

The above compound was synthesized according to General Procedure **B** using **1h** (62 mg, 0.25 mmol) and phenyl vinyl sulfone (84 mg, 0.5 mmol) and irradiated with blue light for 64 h. Crude dr: 9:1 Silica gel column chromatography (Acetone:Pentane 5:95 – 20:80 v:v) to give **3h** as a white solid, 73% (75 mg, isolated as >20:1 dr). **mp** (from Acetone:Pentane): 202-206 °C. **FT-IR** (thin film): ν_{max} (cm^{-1}) = 3373, 2922, 2851, 1509, 1491, 1463, 1448, 1410, 1339, 1302 (S=O). **^{1}H NMR** (400 MHz, DMSO-d_6) δ 7.94 – 7.85 (m, 2H), 7.83 – 7.75 (m, 1H), 7.69 (dd, *J* = 8.4, 7.0 Hz, 2H), 7.47 – 7.30 (m, 4H), 6.75 (dd, *J* = 8.8, 2.9 Hz, 1H), 6.64 (d, *J* = 8.8 Hz, 1H), 6.39 (d, *J* = 2.9 Hz, 1H), 6.15 (s, 1H), 4.73 – 4.65 (m, 1H), 4.58 (dd, *J* = 12.3, 3.0 Hz, 1H), 3.49 (s, 3H), 2.10 (dq, *J* = 13.4, 1.7 Hz, 1H), 1.89 (ddd, *J* = 14.6, 12.3, 5.9 Hz, 1H). **^{13}C NMR** (101 MHz, DMSO) δ 149.8, 142.5, 141.1, 137.5, 134.1, 131.9, 129.5, 129.0, 128.5, 128.5, 117.1, 115.8, 108.8, 61.3, 55.2, 50.0, 30.5. **HRMS** (ESI): m/z calculated for $C_{22}H_{21}O_3N_1Cl_1S_1$ requires 414.0925 for $[M+H]^+$, found 414.0927.

Synthesis of **3j**

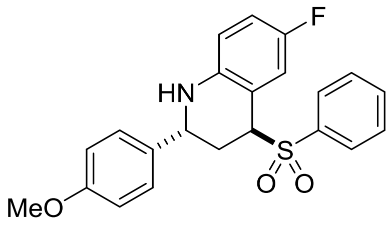

The above compound was synthesized according to General Procedure **B** using **1j** (57 mg, 0.25 mmol) and phenyl vinyl sulfone (84 mg, 0.5 mmol) and irradiated with blue light for 16 h. Crude dr: 7:1. Silica gel column chromatography (CH_2Cl_2:Pentane 10:90 – 50:50 v:v) to give **3j** as a white solid, 73% (73 mg, isolated as 13:1 dr). **mp** (from CH_2Cl_2:Pentane): 184-188 °C. **FT-IR** (thin film): ν_{max} (cm^{-1}) = 3376, 2980, 1504, 1304,. **^{1}H NMR** (400 MHz, DMSO-d_6) δ 7.91 (dd, *J* = 8.4, 1.3 Hz, 2H), 7.85 – 7.77 (m, 1H), 7.75 – 7.67 (m, 2H), 7.27 (d, *J* = 8.7 Hz, 2H), 7.04 – 6.86 (m, 3H), 6.78 – 6.59 (m, 2H), 6.38 (s, 1H), 4.72 (dd, *J* = 5.8, 1.6 Hz, 1H), 4.49 (dd, *J* = 11.9, 3.4 Hz, 1H), 3.74 (s, 3H), 2.02 – 1.78 (m, 2H). **^{19}F NMR** (377 MHz, DMSO-d_6) -130.12 (td, *J* = 9.0, 4.7 Hz) – minor diastereomer δ -127.17 (td, *J* = 9.2, 5.4 Hz**).** **^{13}C NMR** (101 MHz, DMSO-d_6) δ 158.8, 153.1 (d, *J* = 230.5 Hz), 143.8, 137.1, 134.9, 134.2, 129.5, 128.9, 127.8, 117.4 (d, *J* = 22.6 Hz), 116.6 (d, *J* = 22.8 Hz), 115.4 (d, *J* = 7.2 Hz), 113.9, 108.4, 61.0, 55.1, 49.6, 30.3. **HRMS** (ESI): m/z calculated for $C_{22}H_{21}O_3N_1F_1S_1$ requires 398.1221, found 398.1219.

Synthesis of **3k**

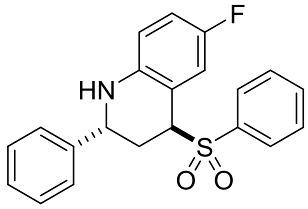

The above compound was synthesized according to General Procedure **B** using **1k** (50 mg, 0.25 mmol) and phenyl vinyl sulfone (84 mg, 0.5 mmol) and irradiated with blue light for 16 h. Crude dr: 7:1. Silica gel column chromatography (CH_2Cl_2:Pentane 10:90 – 50:50 v:v) to give **3k** as a white solid, 65% (60 mg, isolated as >20:1 dr). **mp** (from CH_2Cl_2:Pentane): 175-180 °C. **FT-IR** (thin film): ν_{max} (cm^{-1}) = 3377, 2980, 1504, 1383, 1304. **^{1}H NMR** (400 MHz, DMSO-*d$_6$*) δ 7.97 – 7.87 (m, 2H), 7.83 – 7.76 (m, 1H), 7.70 (t, *J* = 7.6 Hz, 2H), 7.36 (d, *J* = 5.2 Hz, 4H), 7.33 – 7.27 (m, 1H), 6.98 (td, *J* = 8.7, 3.1 Hz, 1H), 6.81 – 6.64 (m, 2H), 6.46 (s, 1H), 4.74 (dd, *J* = 5.8, 1.6 Hz, 1H), 4.55 (dd, *J* = 12.2, 3.3 Hz, 1H), 2.01 (ddd, *J* = 14.7, 3.4, 1.6 Hz, 1H), 1.90 (ddd, *J* = 14.7, 12.1, 5.8 Hz, 1H). **^{19}F NMR** (377 MHz, DMSO-*d$_6$*) δ -125.22 (td, *J* = 8.9, 5.0 Hz) – minor diastereomer δ -122.28 (td, *J* = 9.6, 9.1, 5.2 Hz). **^{13}C NMR** (101 MHz, DMSO-*d$_6$*) δ 153.2 (d, *J* = 231.0 Hz), 143.7, 143.1, 137.1, 134.3, 129.2 (d, *J* = 62.3 Hz), 128.6, 127.7, 126.7, 117.4 (d, *J* = 22.7 Hz), 116.7 (d, *J* = 22.3 Hz), 115.5 (d, *J* = 7.4 Hz), 108.5 (d, *J* = 7.2 Hz), 60.9, 50.3, 30.3. **HRMS** (ESI): m/z calculated for $C_{21}H_{19}O_2N_1F_1S_1$ requires 368.1115 for $[M+H]^+$, found 368.1115.

Synthesis of **3l**

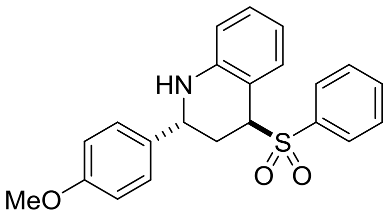

The above compound was synthesized according to General Procedure **B** using **1l** (53 mg, 0.25 mmol) and phenyl vinyl sulfone (84 mg, 0.5 mmol) and irradiated with blue light for 64 h. Crude dr: >20:1 Silica gel column chromatography (CH_2Cl_2:Pentane 20:80 – 90:10 v:v) to give **3l** as a white solid, 70% (66 mg, isolated as >20:1 dr). **mp** (from CH_2Cl_2:Pentane): 178-180 °C. **FT-IR** (thin film): ν_{max} (cm^{-1}) = 3381, 2980, 1608, 1511, 1446, 1303. **^{1}H NMR** (400 MHz, $CDCl_3$) δ 7.85 – 7.75 (m, 2H), 7.75 – 7.63 (m, 1H), 7.54 (dd, *J* = 8.3, 7.1 Hz, 2H), 7.33 – 7.27 (m, 2H), 7.20 – 7.04 (m, 1H), 6.94 – 6.86 (m, 2H), 6.83 – 6.77 (m, 1H), 6.70 – 6.43 (m, 2H), 4.75 (dd, *J* = 12.4, 3.3 Hz, 1H), 4.31 (dd, *J* = 5.8, 1.6 Hz, 1H), 4.21 (s, 1H), 3.81 (s, 3H), 2.58 (ddt, *J* = 14.7, 3.3, 1.4 Hz, 1H), 2.05 (ddd, *J* = 14.7, 12.4, 5.7 Hz, 1H). **^{13}C NMR** (101 MHz, $CDCl_3$) δ 159.5, 146.1, 138.0, 135.1, 133.9, 132.1, 130.2, 129.5, 129.2, 127.9, 116.7, 114.9, 114.2, 109.3, 63.4, 55.4, 51.0, 30.7. **HRMS** (ESI): m/z calculated for $C_{22}H_{22}O_3N_1S_1$ requires 380.1315 for $[M+H]^+$, found 380.1318.

Synthesis of **3m**

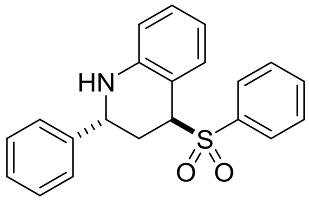

The above compound was synthesized according to General Procedure **B** using **1m** (45 mg, 0.25 mmol) and phenyl vinyl sulfone (84 mg, 0.5 mmol) and irradiated with blue light for 16 h. Crude dr: 10:1. Silica gel column chromatography (CH_2Cl_2:Pentane 20:80 – 80:20 v:v) to give **3m** as a pale pink solid, 80% (70 mg, isolated as 10:1 dr). The product was then triturated with $CHCl_3$:Pentane to obtain single diastereomer for analysis. **mp** (from CH_2Cl_2:Pentane): 190-194 °C. **FT-IR** (thin film): ν_{max} (cm^{-1}) =3383, 2980, 1383, 1252. **^{1}H NMR** (400 MHz, DMSO-d_6) δ 7.90 (d, *J* = 7.6 Hz, 2H), 7.78 (t, *J* = 7.4 Hz, 1H), 7.68 (t, *J* = 7.6 Hz, 2H), 7.37 (d, *J* = 4.4 Hz, 4H), 7.34 – 7.24 (m, 1H), 7.06 (t, *J* = 7.7 Hz, 1H), 6.91 (d, *J* = 7.7 Hz, 1H), 6.70 (d, *J* = 8.2 Hz, 1H), 6.58 – 6.39 (m, 2H), 4.68 (d, *J* = 5.3 Hz, 1H), 4.61 (dd, *J* = 12.2, 3.4 Hz, 1H), 2.03 (dd, *J* = 14.7, 3.3 Hz, 1H), 1.91 (ddd, *J* = 14.5, 12.1, 5.7 Hz, 1H). **^{13}C NMR** (101 MHz, DMSO) δ 146.9, 143.3, 137.4, 134.1, 132.0, 129.4, 129.4, 128.9, 128.6, 127.6, 126.6, 115.02, 114.6, 108.0, 61.3, 50.3, 30.6. **HRMS** (ESI): m/z calculated for $C_{21}H_{20}O_2N_1S_1$ requires 350.1209 for $[M+H]^+$, found 350.1208.

Synthesis of **3n**

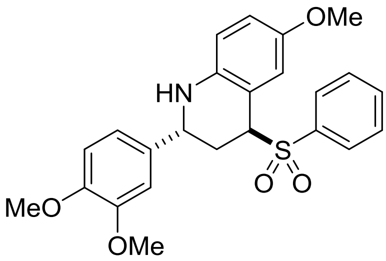

The above compound was synthesized according to General Procedure **B** using **1n** (68 mg, 0.25 mmol) and phenyl vinyl sulfone (84 mg, 0.5 mmol) and irradiated with blue light for 40 h. Crude dr: 8:1. Silica gel column chromatography (EtOAc:Pentane 10:90 – 40:60 v:v) to give **3n** as a white solid, 92% (100 mg, isolated as 9:1 dr). **mp** (from EtOAc:Pentane): 162-166 °C. **FT-IR** (thin film): ν_{max} (cm^{-1}) = 3367, 2980, 1505, 1382. **^{1}H NMR** (400 MHz, DMSO-d_6:$CDCl_3$ 1:5) δ 7.85 – 7.79 (m, 2H), 7.71 – 7.64 (m, 1H), 7.55 (t, *J* = 7.7 Hz, 2H), 6.95 – 6.89 (m, 2H), 6.85 – 6.69 (m, 2H), 6.55 (d, *J* = 8.8 Hz, 1H), 6.33 (d, *J* = 2.8 Hz, 1H), 4.69 (dd, *J* = 12.4, 3.0 Hz, 1H), 4.29 (d, *J* = 5.6 Hz, 1H), 3.86 (s, 6H), 3.55 (s, 3H), 2.59 (ddd, *J* = 14.7, 3.2, 1.5 Hz, 1H), 2.05 (ddd, *J* = 14.7, 12.4, 5.9 Hz, 1H). **^{13}C NMR** (101 MHz, DMSO-d_6:$CDCl_3$ 1:5) δ 151.1, 149.3, 148.7, 140.5, 138.0, 135.8, 133.9, 129.5, 129.2, 118.9, 118.1, 116.3, 115.7, 111.3, 109.8, 109.7, 63.4, 56.0, 56.0, 55.7, 51.6, 30.9. **HRMS** (ESI): m/z calculated for $C_{24}H_{26}O_5N_1S_1$ requires 440.1526 for $[M+H]^+$, found 440.1526.

Synthesis of **3o**

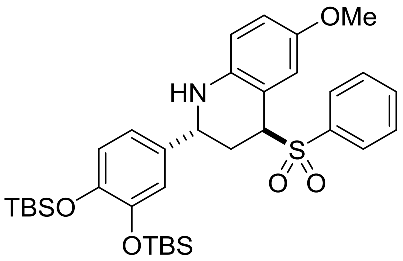

The above compound was synthesized according to General Procedure **B** using **1o** (118 mg, 0.25 mmol) and phenyl vinyl sulfone (84 mg, 0.5 mmol) and irradiated with blue light for 16 h. Crude dr: 8:1. Silica gel column chromatography (EtOAc:Pentane 10:90 – 40:60 v:v) to give **3o** as an off-white solid, 40% (64 mg, isolated as 8:1 dr). **FTIR** (thin film): ν_{max} (cm^{-1}) = 3372 (NH), 2955, 2930, 2895, 2858, 1604, 1579, 1303 (SO) **^{1}H NMR** (400 MHz, DMSO-d_6) δ 7.90 – 7.85 (m, 2H), 7.81 – 7.74 (m, 1H), 7.68 (t, *J* = 7.6 Hz, 2H), 6.83 (d, *J* = 1.1 Hz, 2H), 6.79 – 6.71 (m, 2H), 6.65 (d, *J* = 8.8 Hz, 1H), 6.48 (d, *J* = 2.9 Hz, 1H), 6.06 (s, 1H), 4.72 – 4.62 (m, 1H), 4.40 (dd, *J* = 12.1, 3.0 Hz, 1H), 3.52 (s, 3H), 2.03 (ddd, *J* = 14.6, 3.1, 1.5 Hz, 1H), 1.82 (ddd, *J* = 14.6, 12.2, 6.0 Hz, 1H), 0.95 (d, *J* = 1.2 Hz, 18H), 0.20 – 0.15 (m, 12H). **^{13}C NMR** (101 MHz, DMSO-d_6) δ 149.7, 146.0, 145.4, 141.4, 137.6, 136.8, 134.0, 129.4, 128.8, 120.7, 119.6, 119.0, 117.0, 115.9, 115.8, 108.6, 61.4, 55.2, 49.8, 31.0, 25.8, 25.7, 18.1, -4.2. **HRMS** (ESI): m/z calculated for $C_{34}H_{50}O_5S_1Si_2$ requires 640.2943 for $[M+H]^+$, found 640.2940.

Synthesis of **3p**

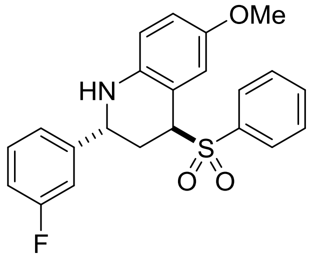

The above compound was synthesized according to General Procedure **B** using **1p** (57 mg, 0.25 mmol) and phenyl vinyl sulfone (84 mg, 0.5 mmol) and irradiated with blue light for 64 h. Crude dr: 7:1. Silica gel column chromatography (EtOAc:Pentane 5:95 – 40:60 v:v) to give **3p** as a pale orange solid, 62% (60 mg, isolated as 7:1 dr). **mp** (from EtOAc:Pentane): 160-164 °C. **FT-IR** (thin film): ν_{max} (cm^{-1}) = 3368 (NH), 2980 (CH), 1591, 1507, 1382, 1303 (SO). **^{1}H NMR** (400 MHz, DMSO-d_6) δ 7.91 (dd, *J* = 8.3, 1.3 Hz, 2H), 7.82 – 7.75 (m, 1H), 7.70 (dd, *J* = 8.3, 6.9 Hz, 2H), 7.41 (td, *J* = 8.0, 6.0 Hz, 1H), 7.23 – 7.16 (m, 2H), 7.16 – 7.07 (m, 1H), 6.76 (dd, *J* = 8.8, 2.9 Hz, 1H), 6.66 (d, *J* = 8.8 Hz, 1H), 6.42 (d, *J* = 2.9 Hz, 1H), 6.18 (s, 1H), 4.72 – 4.67 (m, 1H), 4.61 (dd, *J* = 12.3, 3.0 Hz, 1H), 3.50 (s, 3H), 2.18 – 2.04 (m, 1H), 1.92 (ddd, *J* = 14.6, 12.3, 5.9 Hz, 1H). **^{19}F NMR** (377 MHz, DMSO-d_6) δ -112.85 (td, *J* = 9.6, 5.7 Hz) – minor diastereomer δ -112.97 (td, *J* = 9.5, 5.9 Hz). **^{13}C NMR** (101 MHz, DMSO-d_6) δ 162.8 (d, *J* = 243.3 Hz), 150.3, 147.0 (d, *J* = 7.1 Hz), 141.5, 137.9, 134.6, 131.0 (d, *J* = 8.2 Hz), 129.7 (d, *J* = 51.0 Hz), 123.3, 117.6, 116.3, 114.8 (d, *J* = 21.2 Hz), 113.7 (d, *J* = 22.0 Hz), 109.3, 61.8, 55.7, 50.6, 30.9. **HRMS** (ESI): m/z calculated for $C_{22}H_{21}O_3N_1F_1S_1$ requires 398.1221 for $[M+H]^+$, found 398.1222.

Synthesis of **3q**

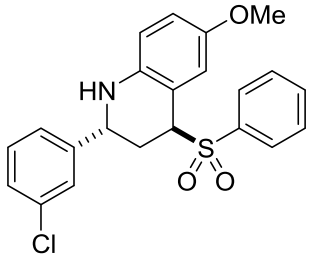

The above compound was synthesized using General Procedure **B** using **1q** (62 mg, 0.25 mmol) and phenyl vinyl sulfone (84 mg, 0.5 mmol) and irradiated with blue light for 64 h. Crude dr: 9:1. Silica gel column chromatography (CH_2Cl_2:Pentane 5:95 – 40:60 v:v) to give **3q** as off-white powdery solid, 56% (58 mg – isolated as 14:1 dr). **mp** (from CH_2Cl_2:Pentane): 172-176 °C. **FT-IR** (thin film): ν_{max} (cm^{-1}) = 3370, 2980, 1506, 1383. **^{1}H NMR** (400 MHz, DMSO-d_6:$CDCl_3$ 1:1) δ 7.85 (dd, *J* = 8.1, 2.8 Hz, 2H), 7.71 (dd, *J* = 7.4, 2.4 Hz, 1H), 7.66 – 7.57 (m, 2H), 7.37 (t, *J* = 2.2 Hz, 1H), 7.34 – 7.21 (m, 3H), 6.78 – 6.57 (m, 2H), 6.35 (d, *J* = 3.0 Hz, 1H), 5.96 (d, *J* = 2.9 Hz, 1H), 4.61 (dd, *J* = 12.6, 3.1 Hz, 1H), 4.48 (t, *J* = 4.1 Hz, 1H), 3.49 (s, 3H), 2.23 (d, *J* = 14.5 Hz, 1H), 2.01 – 1.73 (m, 1H). **^{13}C NMR** (101 MHz, DMSO-d_6:$CDCl_3$ 1:1) δ 149.8, 145.7, 140.7, 137.3, 133.6, 133.4, 129.8, 129.0, 128.7, 127.2, 126.2, 125.0, 117.0, 115.8, 115.4, 108.5, 61.6, 54.9, 50.1, 30.4. **HRMS** (ESI): m/z calculated for $C_{22}H_{21}O_3N_1Cl_1S_1$ requires 414.0925 for $[M+H]^+$, found 414.0929.

Synthesis of **3r**

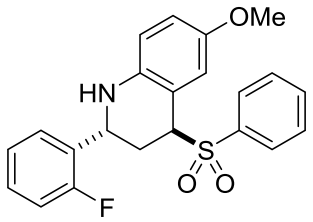

The above compound was synthesized according to General Procedure **B** using **1r** (57 mg, 0.25 mmol) and phenyl vinyl sulfone (84 mg, 0.5 mmol) and irradiated with blue light for 64 h. Crude dr: 8:1. Silica gel column chromatography (EtOAc:Pentane 5:95 – 40:60 v:v) to give **3r** as an amorphous solid, 44% (44 mg – isolated as 8:1 dr). **FT-IR** (thin film): ν_{max} (cm^{-1}) = 3364, 2980, 1507, 1383, 1251. **^{1}H NMR** (400 MHz, $CDCl_3$) δ 7.86 (dd, *J* = 8.4, 1.4 Hz, 2H), 7.68 – 7.63 (m, 1H), 7.59 – 7.47 (m, 3H), 7.30 – 7.20 (m, 1H), 7.13 (td, *J* = 7.6, 1.2 Hz, 1H), 7.04 (ddd, *J* = 10.5, 8.3, 1.3 Hz, 1H), 6.81 (dd, *J* = 8.8, 2.9 Hz, 1H), 6.75 (d, *J* = 2.9 Hz, 1H), 6.56 (d, *J* = 8.8 Hz, 1H), 5.02 (dd, *J* = 12.4, 3.0 Hz, 1H), 4.31 (dd, *J* = 5.9, 1.6 Hz, 1H), 3.89 (s, 1H), 3.68 (s, 3H), 2.56 (ddd, *J* = 14.5, 3.0, 1.5 Hz, 1H), 2.08 – 1.95 (m, 1H). **^{19}F NMR** (377 MHz, $CDCl_3$) δ -118.65 (dt, *J* = 11.3, 6.2 Hz) – minor diastereomer δ -119.54 (dt, *J* = 12.0, 6.1 Hz). **^{13}C NMR** (101 MHz, $CDCl_3$) δ 160.5 (d, *J* = 247.7 Hz), 151.7, 140.4, 137.7, 133.9, 130.0 (d, *J* = 12.0 Hz), 129.6, 129.8 – 128.7 (app t), 129.3, 127.6 (d, *J* = 4.1 Hz), 124.6 (d, *J* = 3.7 Hz), 118.2, 116.6, 115.9, 115.6 (d, *J* = 21.5 Hz), 109.9, 63.1, 55.9, 44.6 (d, *J* = 3.3 Hz), 29.6. **HRMS** (ESI): m/z calculated for $C_{22}H_{21}O_3N_1F_1S_2$ requires 398.1221 for $[M+H]^+$, found 398.1226.

Synthesis of **3s**

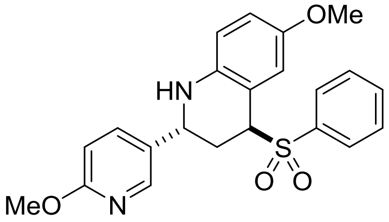

The above compound was synthesized according to General Procedure **B** using **1s** (61 mg, 0.25 mmol) and phenyl vinyl sulfone (84 mg, 0.5 mmol) and irradiated with blue light for 40 h. Crude dr: 8:1. Silica gel column chromatography (EtOAc:Pentane 20:80 – 40:60 v:v) to give **3s** as a powdery off-white solid, 94% (96 mg – isolated as 11:1 dr). **mp** (from $CHCl_3$) = 150-154 °C. **FTIR** (thin film): ν_{max} (cm^{-1}) = 3369 (NH), 2947, 2835, 1608, 1575, 1505, 1304 (SO) ^{1}**H NMR** (400 MHz, DMSO-d_6) δ 8.13 (d, *J* = 2.4 Hz, 1H), 7.95 – 7.86 (m, 2H), 7.78 (ddt, *J* = 6.6, 4.9, 1.3 Hz, 1H), 7.69 (ddd, *J* = 7.1, 5.5, 2.0 Hz, 2H), 6.81 (d, *J* = 8.6 Hz, 1H), 6.76 (dd, *J* = 8.8, 2.9 Hz, 1H), 6.63 (d, *J* = 8.9 Hz, 1H), 6.44 (d, *J* = 2.9 Hz, 1H), 6.07 (s, 1H), 4.73 – 4.64 (m, 1H), 4.57 (dd, *J* = 12.0, 3.6 Hz, 1H), 3.84 (s, 3H), 3.51 (s, 3H), 2.06 – 1.88 (m, 2H). ^{13}**C NMR** (101 MHz, DMSO-d_6) δ 163.3, 149.8, 145.1, 141.1, 137.7, 137.4, 134.0, 131.7, 129.4, 128.9, 117.0, 115.9, 115.7, 110.5, 108.8, 61.4, 55.2, 53.1, 47.7, 30.0. **HRMS** (ESI): m/z calculated $C_{22}H_{23}O_4N_2S_1$ requires 411.1373 for $[M+H]^+$, found 411.1369.

Synthesis of **3ab**

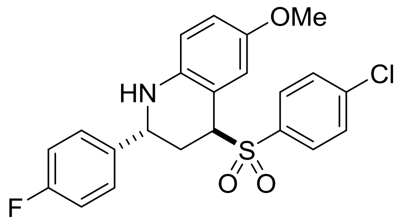

The above compound was synthesized according to General Procedure **B** using **1a** (57 mg, 0.25 mmol) and (4-chlorophenyl) vinyl sulfone (101 mg, 0.5 mmol) and irradiated with blue light for 40 h. Crude dr: 10:1. Silica gel column chromatography (Acetone:Pentane 5:95 – 20:80 v:v) to give **3ab** as a powdery white solid, 90% (97 mg – isolated as 10:1 dr). **mp** (from Acetone:Pentane): 176-180 °C. **FTIR** (thin film): ν_{max} (cm^{-1}) = 3376 (NH), 2980 (CH), 1506, 1475, 1394, 1310 (SO). **^{1}H NMR** (400 MHz, DMSO-d_6) δ 7.90 (d, *J* = 8.5 Hz, 3H), 7.81 – 7.74 (m, 2H), 7.45 – 7.38 (m, 2H), 7.24 – 7.09 (m, 2H), 6.76 (dd, *J* = 8.9, 2.9 Hz, 1H), 6.65 (d, *J* = 8.9 Hz, 1H), 6.43 (d, *J* = 2.9 Hz, 1H), 6.15 (s, 1H), 4.79 – 4.70 (m, 1H), 4.64 – 4.54 (m, 1H), 3.53 (s, 3H), 2.10 (dd, *J* = 14.5, 2.9 Hz, 1H), 1.93 (ddd, *J* = 14.6, 12.3, 5.8 Hz, 1H). **^{19}F NMR** (377 MHz, DMSO-d_6) -114.98 (ddd, *J* = 14.3, 9.1, 5.3 Hz).- minor diastereomer δ -114.85 (td, *J* = 9.1, 4.7 Hz). **^{13}C NMR** (101 MHz, DMSO-d_6) δ 161.6 (d, *J* = 242.8 Hz), 149.7, 141.2, 139.6 (d, *J* = 3.0 Hz), 139.3, 136.4, 131.0, 129.7, 129.6, 128.6 (d, *J* = 8.2 Hz), 117.20, 115.9, 115.3 (d, *J* = 21.1 Hz), 108.41, 61.5, 55.2, 49.9, 30.5. **HRMS** (ESI): m/z calculated for $C_{22}H_{20}O_3N_1Cl_1F_1S_1$ requires 432.0831 for $[M+H]^+$, found 432.0834.

Synthesis of **3ac**

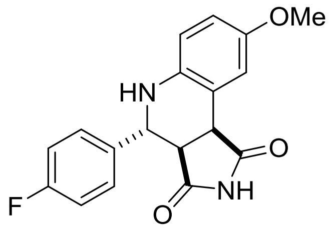

The above compound was synthesized according to General Procedure **B** using **1a** (57 mg, 0.25 mmol) and maleimide (121 mg, 1.25 mmol) and irradiated with blue light for 16 h. Crude dr: 3:1. Silica gel column chromatography (EtOAc:Pentane 10:90 – 40:60 v:v) to give **3ac** as a yellow crystalline solid, 87% (71 mg – isolated as 3:1 dr). Trituration to increase diastereomeric ratio for analysis using $CHCl_3$:Pentane was unsuccessful. **mp** (from EtOAc:Pentane): 116-120 °C. **FT-IR** (thin film): ν_{max} (cm^{-1}) = 3298, 3220, 2980, 1713, 1506, 1382. **^{1}H NMR** (400 MHz, DMSO-d_6) δ 11.32 (s, 1H), 7.35 (dd, *J* = 8.7, 5.6 Hz, 2H), 7.11 (td, *J* = 9.1, 7.0 Hz, 2H), 6.89 (dd, *J* = 16.2, 2.7 Hz, 1H), 6.67 – 6.55 (m, 2H), 6.01 – 5.92 (m, 1H), 4.49 (dd, *J* = 4.3, 2.3 Hz, 1H), 3.98 (d, *J* = 8.9 Hz, 1H), 3.64 (s, 3H). **^{19}F NMR** (377 MHz, DMSO-d_6) -116.08 (tt, *J* = 9.5, 5.5 Hz) – minor diastereomer δ -115.80 (tt, *J* = 9.4, 5.4 Hz). **^{13}C NMR** (101 MHz, DMSO-d_6) δ 178.4 (d, *J* = 80.4 Hz), 151.5, 138.4 (d, *J* = 3.9 Hz), 138.3, 129.2, 129.1, 117.3, 116.0, 114.9 (d, *J* = 21.3 Hz), 114.3 (d, *J* = 48.3 Hz), 55.2, 52.8, 47.8, 41.7. **HRMS** (ESI):m/z calculated for $C_{18}H_{16}O_3N_2F_1$ requires 327.1140 for $[M+H]^+$, found 327.1140.

Synthesis of **3ad**

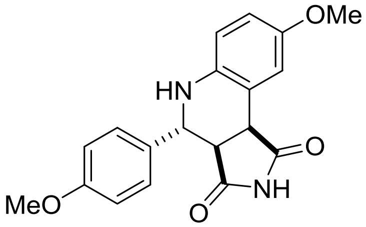

The above compound was synthesized according to General Procedure **B** using **1e** (60 mg, 0.25 mmol) and maleimide (49 mg, 0.5 mmol) and irradiated with blue light for 16 h. Crude dr: 3:1. Silica gel column chromatography (EtOAc:Pentane 10:90 – 40:60 v:v) to give **3ad** as a white solid, 77% (65 mg – isolated as 3:1 dr). Trituration to increase diastereomeric ratio for analysis using $CHCl_3$:Pentane was unsuccessful. **mp** (from EtOAc:Pentane): 205-201 °C. **FT-IR** (thin film): ν_{max} (cm^{-1}) = 3185, 2980, 1714, 1504, 1382. **^{1}H NMR** (400 MHz, DMSO-d_6) δ 11.29 (s, 1H), 7.23 (d, J = 8.5 Hz, 2H), 6.87 – 6.82 (m, 3H), 6.65 – 6.51 (m, 2H), 5.91 (d, J = 2.5 Hz, 1H), 4.46 (t, J = 3.1 Hz, 1H), 3.96 (d, J = 8.9 Hz, 1H), 3.70 (s, 3H), 3.64 (s, 3H), 3.61 (d, J = 4.0 Hz, 1H). **^{13}C NMR** (101 MHz, DMSO-d_6) δ 179.0, 178.0, 158.2, 151.4, 138.5, 134.2, 128.3, 117.3, 116.0, 114.5, 114.0, 113.5, 55.2, 55.0, 52.8, 47.9, 41.8. **HRMS** (ESI): m/z calculated for $C_{19}H_{19}O_4N_2$ requires 339.1339 for $[M+H]^+$, found 339.1342

Synthesis of **3ae**

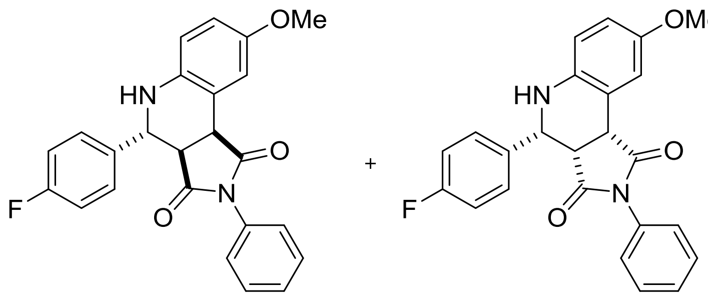

1 - *trans* **1'** - *cis*

The above compound was synthesized according to General Procedure **B** using **1a** (57 mg, 0.25 mmol) and *N*-phenylmaleimide (216 mg, 1.25 mmol) and irradiated with blue light for 64 h. Crude dr: 1:1. Silica gel column chromatography (EtOAc:Pentane 10:90 – 40:60 v:v) to give **3ad** as a white solid, 79% (79 mg – isolated as 4:3 dr). Trituration to increase diastereomeric ratio for analysis using $CHCl_3$:Pentane was unsuccessful. **mp** (from EtOAc:Pentane): 166-170 °C. **FT-IR** (thin film): ν_{max} (cm^{-1}) =2980, 1711, 1382. **^{1}H NMR** (400 MHz, DMSO-d_6) δ 7.49 (dd, *J* = 8.3, 6.7 Hz, 2H, **1** & **1’**), 7.46 – 7.32 (m, 7H, **1** & **1’**), 7.30 – 7.20 (m, 2H, **1** & **1’**), 7.15 (td, *J* = 8.9, 5.9 Hz, 4H, **1** & **1’**), 6.99 (q, *J* = 2.2, 1.5 Hz, 2H, **1** & **1’**), 6.94 – 6.85 (m, 1H, **1**), 6.81 (d, *J* = 8.7 Hz, 1H, **1’**), 6.75 (dd, *J* = 8.7, 2.8 Hz, 1H, **1’**), 6.69 (s, 2H, **1** & **1’**), 6.10 – 6.05 (m, 1H, **1**), 5.99 (s, 1H, **1’**), 4.55 (d, *J* = 5.6 Hz, 2H, **1** & **1’**), 4.37 (d, *J* = 8.9 Hz, 1H), 4.27 (d, *J* = 8.7 Hz, 1H, **1’**), 3.86 (dd, *J* = 8.9, 5.0 Hz, 1H, **1**), 3.76 – 3.71 (m, 2H, **1** & **1’**), 3.70 (s, 3H, **1’**), 3.66 (s, 3H, **1**). **^{19}F NMR** (377 MHz, DMSO-d_6) δ -115.45 (tt, *J* = 9.6, 5.5 Hz) – minor diastereomer δ -115.59 (ddd, *J* = 14.7, 9.6, 5.6 Hz). **^{13}C NMR** (101 MHz, DMSO-d_6) δ 176.1, 175.8, 175.4, 175.0, 162.6, 160.2, 152.0, 151.6, 140.6, 138.8, 138.0, 137.9, 136.6, 132.3, 132.1, 129.5, 129.4, 129.4, 129.3, 129.0, 128.8, 128.4, 128.2, 127.0, 126.6, 117.3, 116.5, 116.3, 116.1, 115.0, 114.9, 114.8, 114.7, 114.6, 114.4, 114.3, 114.2, 59.8, 55.5, 55.3, 55.2, 53.8, 47.1, 47.0, 42.4, 41.0, 40.1, 39.9, 39.7, 39.5, 39.3, 39.1, 38.9. **HRMS** (ESI): m/z calculated for $C_{24}H_{20}O_3N_2F$ requires 403.1453 for $[M+H]^+$, found 403.1451.

Unsuccessful Reactions

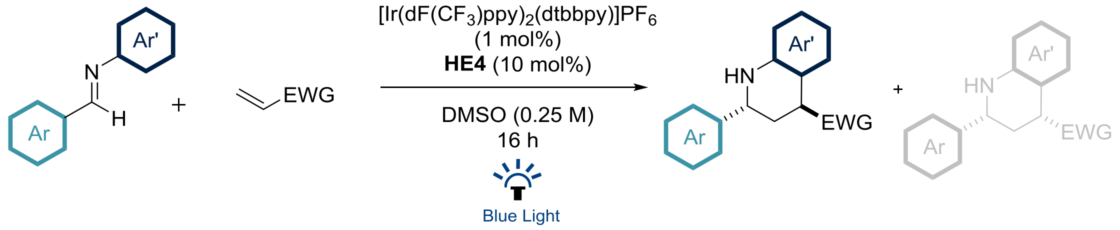

(a) Imine

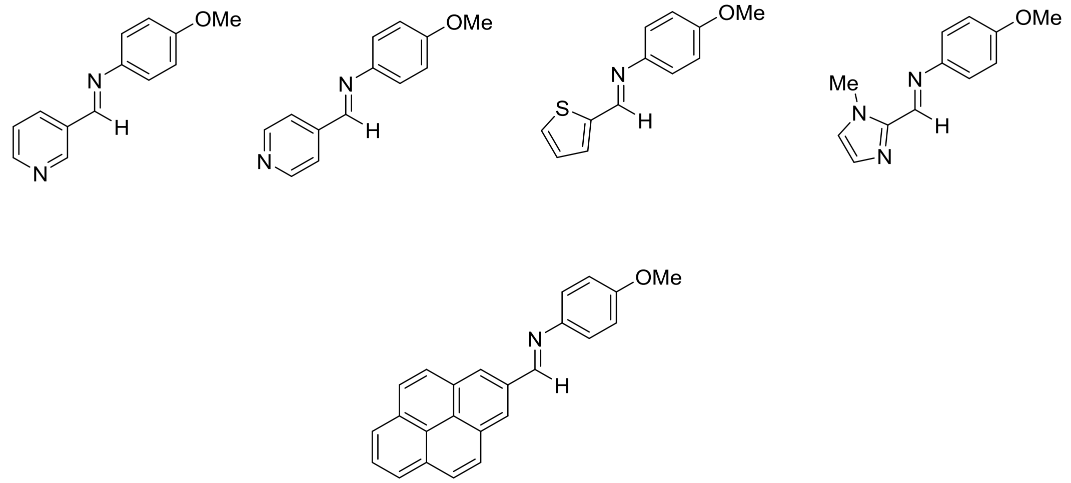

(b) Coupling Partner

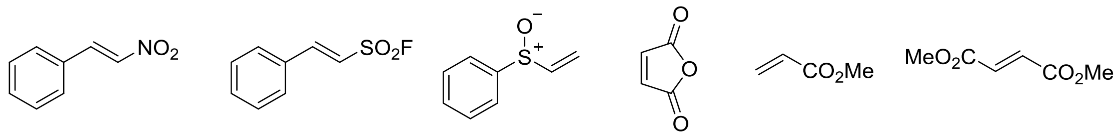

Acrylonitrile was also used in the reaction and was shown to give a mixture of cyclised and Michael addition products in low yields under the optimised conditions.

7. Mechanistic Experiments

7.1: Diastereoselectivity

Scheme 4a (Main Text)

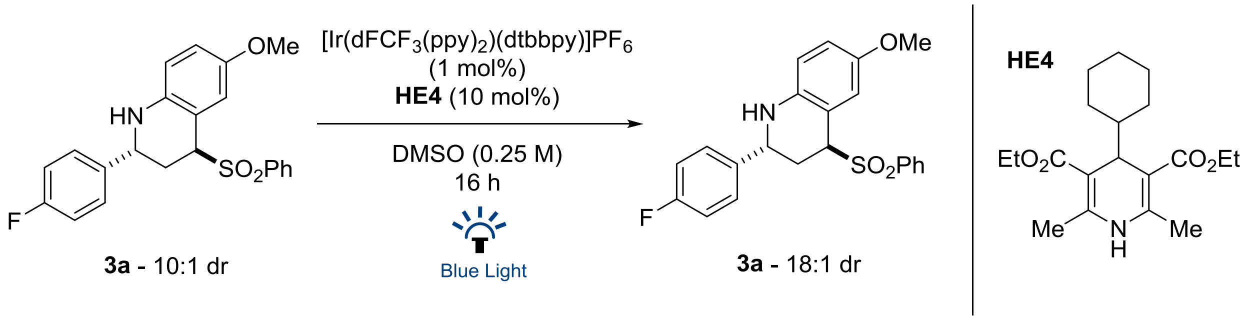

NMR's shown below are $^{19}F\{^1H\}$ NMR

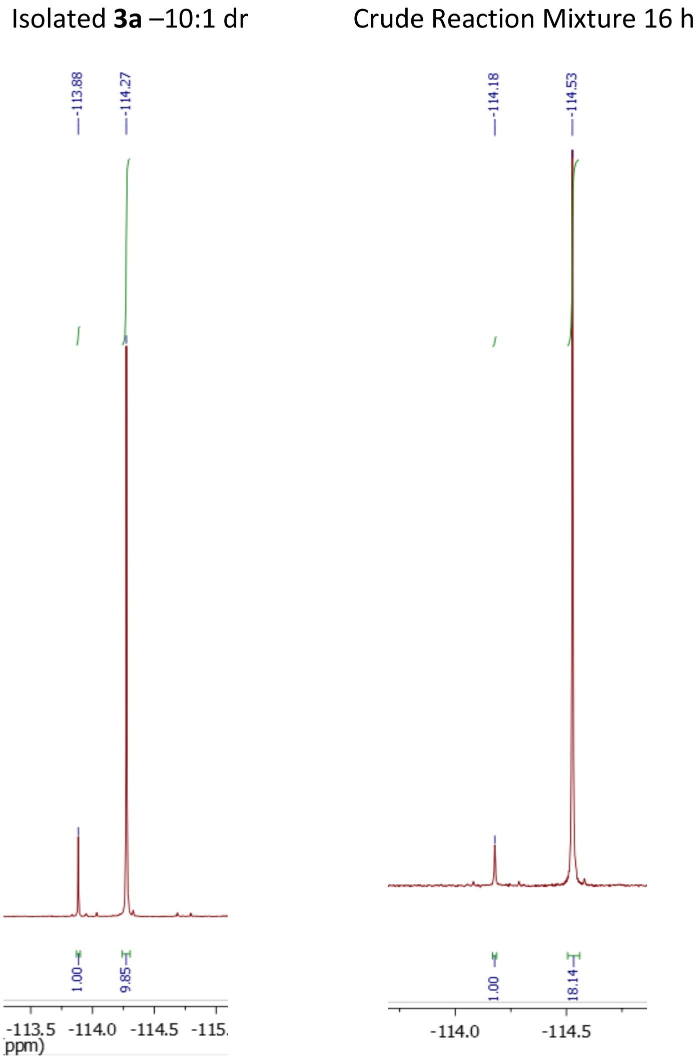

7.2: Luminescence Quenching Studies

A 4.17 x 10^{-5} M solution of $(Ir[dF(CF_3)ppy]_2(dtbbpy))PF_6$ was irradiated at 451.98 nm and the emission intensity was measured between 400-575 nm. $(Ir[dF(CF_3)ppy]_2(dtbbpy))PF_6$ gives an emission peak ~483 nm. Aliquots of the appropriate quencher were added to a stock solution of the iridium photocatalyst in anhydrous DMSO in a volumetric flask. The flask was then made up to 10 mL with anhydrous DMSO, and then degassed with N_2 for 10 minutes. The emission spectra of increasing concentration of the imine (**1a**, blue), HE(Cy) (**HE4**, red) and sulfone (**2a**, purple).

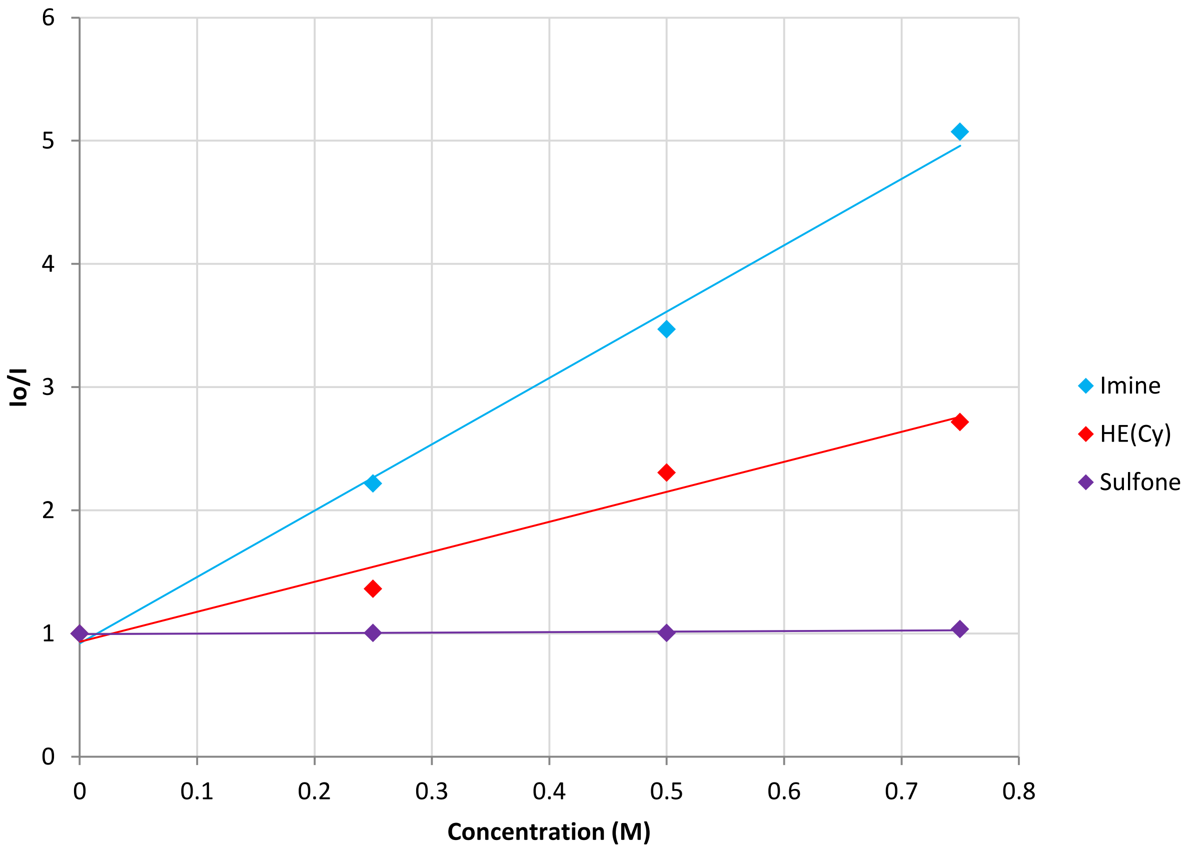

The emission spectra demonstrate that both the imine and Hantzsch ester are capable of quenching the fluorescence. Despite this, a PCET event would make the imine more readily reduced by the excited photocatalyst. However an alternative mechanism can be proposed whereby direct reduction of the imine to the split radical anion intermediate and subsequent proton transfer from an HE oxidised from an off-cycle excited iridium species, could be at play (or a combination of the two). This would again enable a propagating cycle whereby the cationic intermediate can then act as the proton donor for the next cycle.

Despite this, both initiation and propagation cycles contain the same oxidation of the cyclised intermediate. This does not agree with the finding that $[Ru(bpy)_3](PF_6)_2$ is not capable of leading to substantial quantities of product using catalytic quantities of **HE4** (see 7.5: Propagation Investigations)

7.3: Light Dark Experiments

The light dark experiments were carried out using the photoreactor set-up where the light was switched on and off at hourly intervals, where stirring was maintained. Conversions clearly show that the reaction only takes place when the light is on.

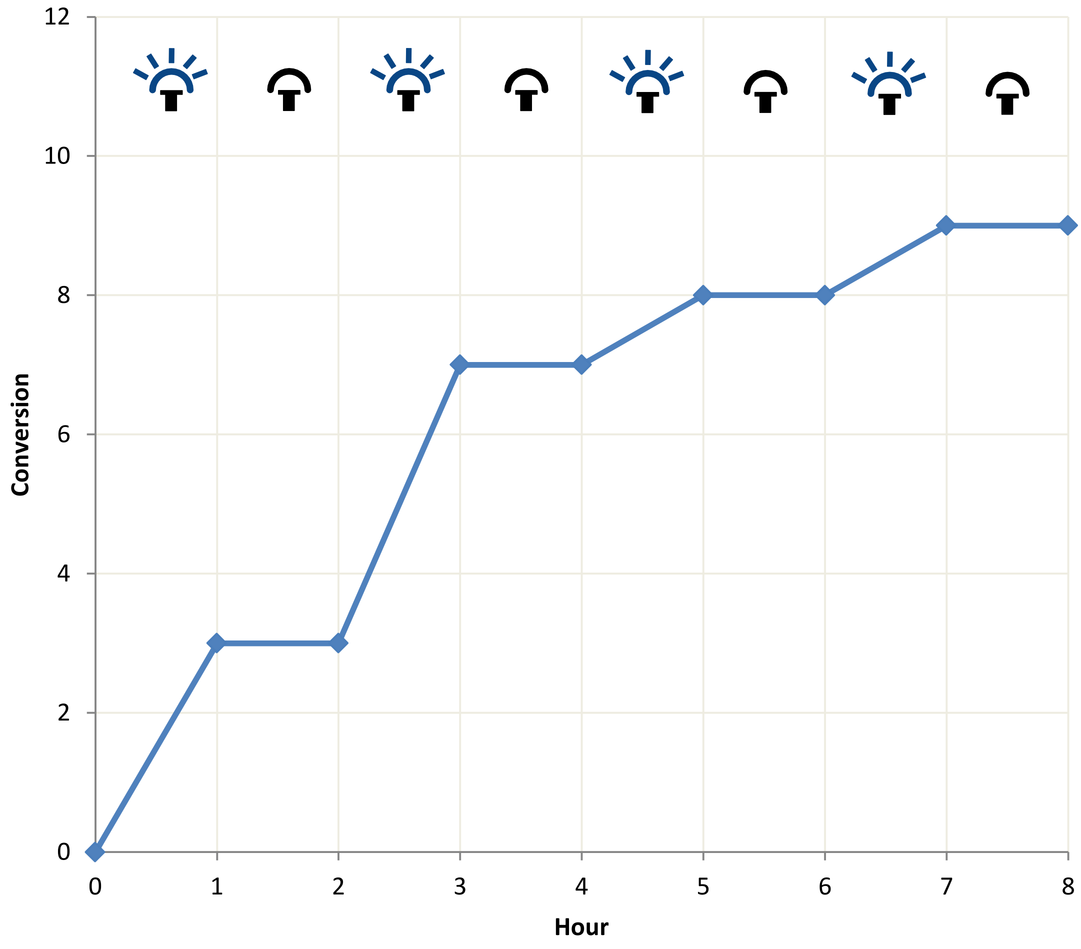

7.4: KIE Experiments

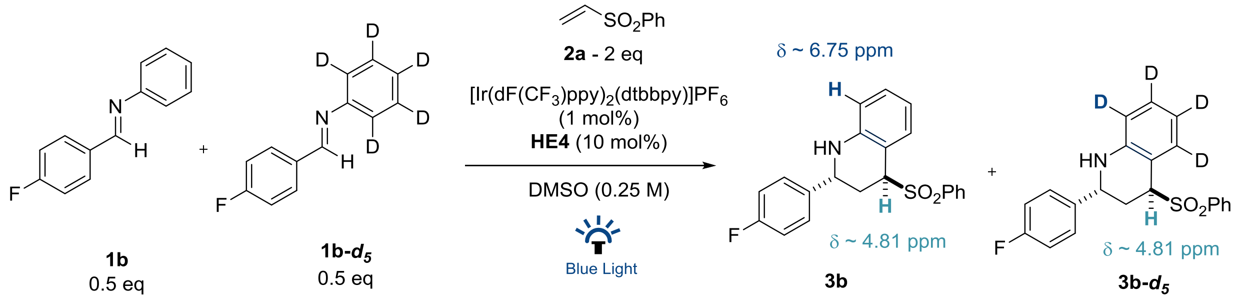

8 hour stop
62% Conversion

50% H - 50% D
KIE = 1.00

---

4 hour stop
34% Conversion

52.6% H 47.4% D
KIE = 1.11

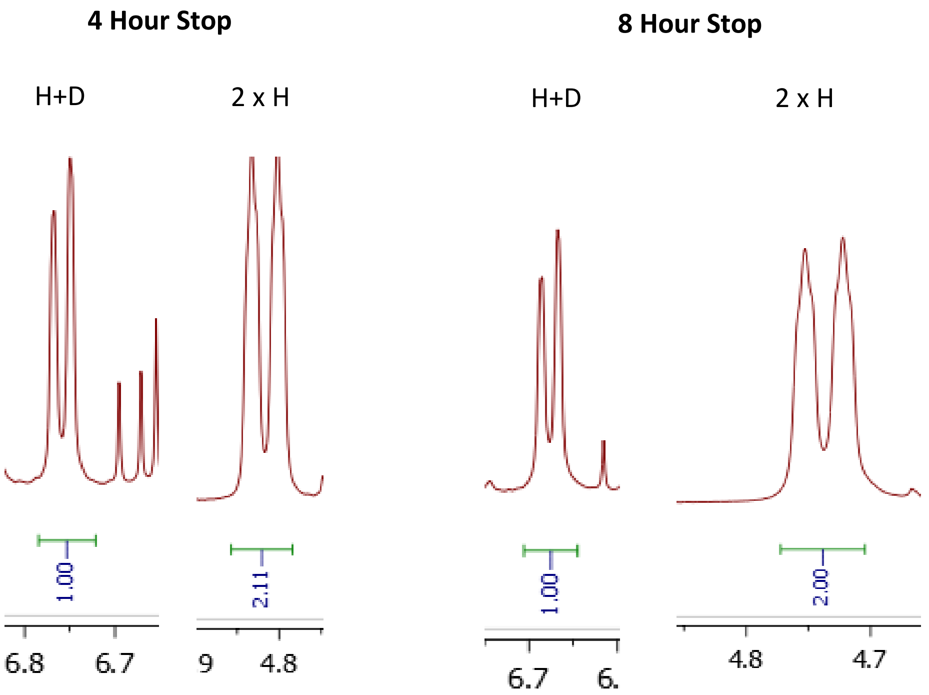

KIE investigations demonstrate that cleavage of the C-H bond on the arene ring is not in the rate limiting step, which suggests that the propagation PCET or proton shuttling takes place readily and is not a stalling point for the reaction mechanism.

7.5: Quantum Yield Determination

7.5.1: Photon flux calculation

The quantum yield determination was carried out using Yoon's method.[26] The photon flux of the light source (Kessil 40W LED light) by standard ferrioxalate actinometry. A 0.15 M solution of ferrioxalate was prepared by dissolving potassium ferrioxalate hydrate(1.84 g) in H_2SO_4 (0.05 M, 25 mL). A buffer solution of phenanthroline was prepared by dissolving 1,10-phenanthroline (50 mg) and sodium acetate (11.25 g) in H_2SO_4 (0.05 M, 50 mL) in a volumetric flask. Both solutions were prepared and stored in the dark.

In a dark room, ferrioxalate solution (2 mL) was added to a cuvette and irradiated for 90 secs at 436 nm. After irradiation, the phenanthroline buffer solution (0.35 mL) was added. The solution was sealed with a Teflon cap, mixed well and then stored in the dark for ~ 30 mins to allow ferrous ions to coordinate phenanthroline. The absorbance of the solution was measured at 510 nm. A non-irradiated sample was also prepared and absorbance measured at 510 nm. The amount of ferrous ions in solution was calculated using eq 1.

$$\text{(1)} \qquad \text{mol } Fe^{2+} = \frac{V \bullet \Delta A}{1 \bullet \varepsilon}$$

Where V is the total volume (0.00235 L) of the solution after addition of phenanthroline bugger, ΔA is the difference in absorbance at 510 nm between irradiated and non-irradiated solutions, 1 is the path length (1.000 cm) and ε is the molar absorptivity at 510 nm (11,100 L mol^{-1} cm^{-1}). The photon flux can then be calculated using eq 2.

$$\text{(2)} \qquad \text{photon flux} = \frac{\text{mol } Fe^{2+}}{\phi \bullet t \bullet f}$$

Where ϕ is the quantum yield for the ferrioxalate actinometer (1.01 for a 0.15 solution at 436 nm), t is the time (90.0 s) and f is the fraction of light absorbed at 436 nm (0.99014 *vide infra*). The photon flux was calculated (average of three experiments) to be 1.52845×10^{-9} einsteins s^{-1}

7.5.2: Quantum yield calculation

A cuvette was charged with **1a** (114 mg, 0.5 mmol), phenyl vinyl sulfone (168 mg, 1 mmol), 4-cyclohexyl-2,6-dimethyl-1,4-dihydropyridine-3,5-dicarboxylate (**HE4**, 16.8 mg, 0.05 mmol), and $[Ir(dF(CF_3)ppy)_2(dtbbpy)]PF_6$ (5.6 mg, 0.005 mmol). To the cuvette was added DMSO (anhydrous, 2 mL). The flask was then degassed for 10 mins, before being sealed with a cuvette screw cap. The sample was irradiated (436 nm) without stirring for 3600 s (60 min). After this time, a sample of the mixture was diluted in $CDCl_3$ and NMR conversion to product calculated using direct conversion between **1a**, **3a** (major + minor diastereomers) & 4-fluorobenzaldehyde (often seen as <3% by-product) *via* $^{19}F\{^1H\}$ NMR spectroscopy. The quantum yield was determined using eq 3, where t = 3600 s, and f = 0.99877 (calculated *vide infra*).

$$\phi = \frac{\text{mol } \mathbf{3a}}{\text{flux} \bullet t \bullet f} \qquad (3)$$

After 1 hour (average of three experiments) the set-up yielded 19.7% **3a** (major + minor), and the quantum yield was calculated to be 18.

Despite the quantum yield suggesting a chain process, a closed shell secondary propagation cycle can not be completely ruled out, especially for different substrates.

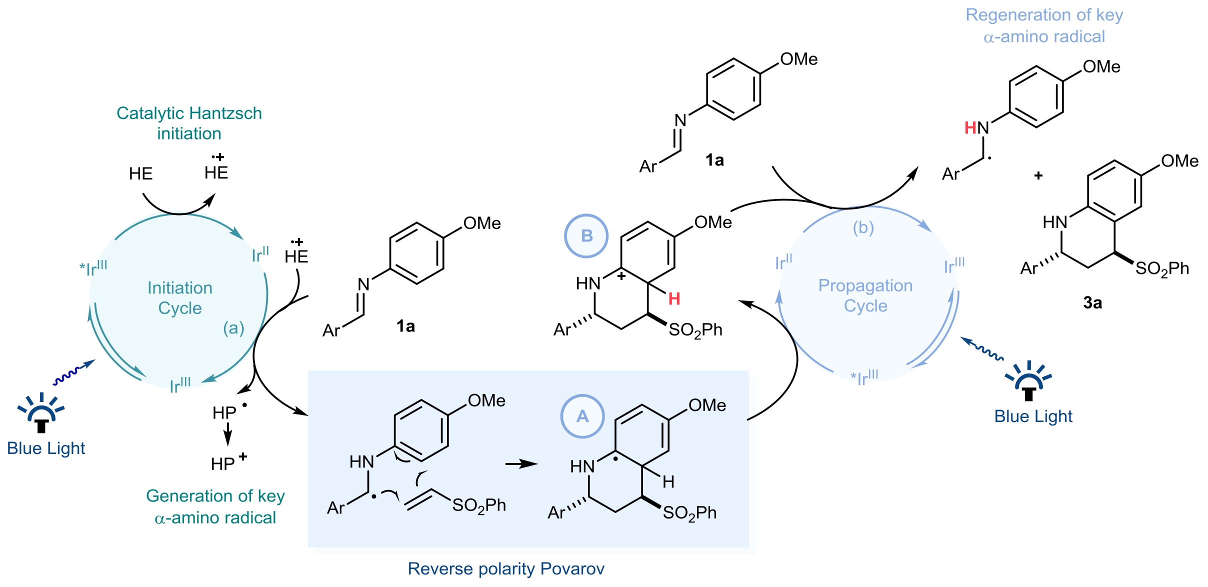

7.6: Plausible Epimerisation Mechanism

We hypothesise that due to allylic strain *trans*-**3a** is the thermodynamically preferred diastereomer. At the cyclisation step both diastereomers are possible and an all equatorial intermediate leading to *cis*-**23** is possible. We then believe that the small amounts of *cis*-**3a** formed can then be transformed into *trans*-**23** under the reaction conditions. We suggest that the tetrahydroquinoline product can quench an iridium species generating the radical cation. Subsequent loss of a proton enables a radical species capable of a 1,2-hydrogen shift. This gives the planar α-amino radical which can act as a point of epimerisation. The epimerised intermediate then regains a proton and an electron from the reduced iridium species to give *trans*-**3a**.

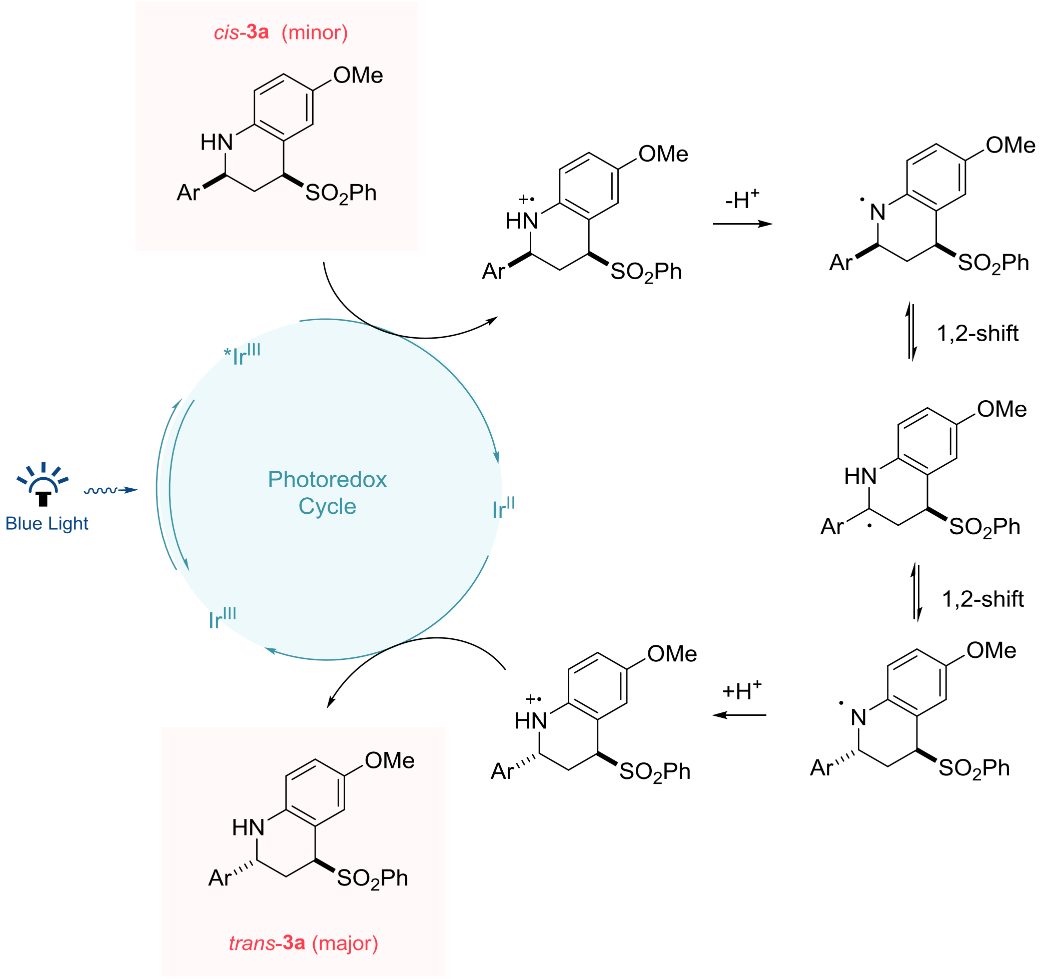

It is proposed that as this secondary cycle does not come into effect until after about 90% conversion, that the Hantzsch ester and the radical intermediate out-compete the secondary amine product **3a**. However towards the end of the reaction with minimum starting material remaining and the substoichiometric Hantzsch partially in the oxidised state, **3a** can act as a reductive quencher for the iridium species.

8. NMR Spectra

1n – ^{1}H NMR (400 MHz, $CDCl_3$)

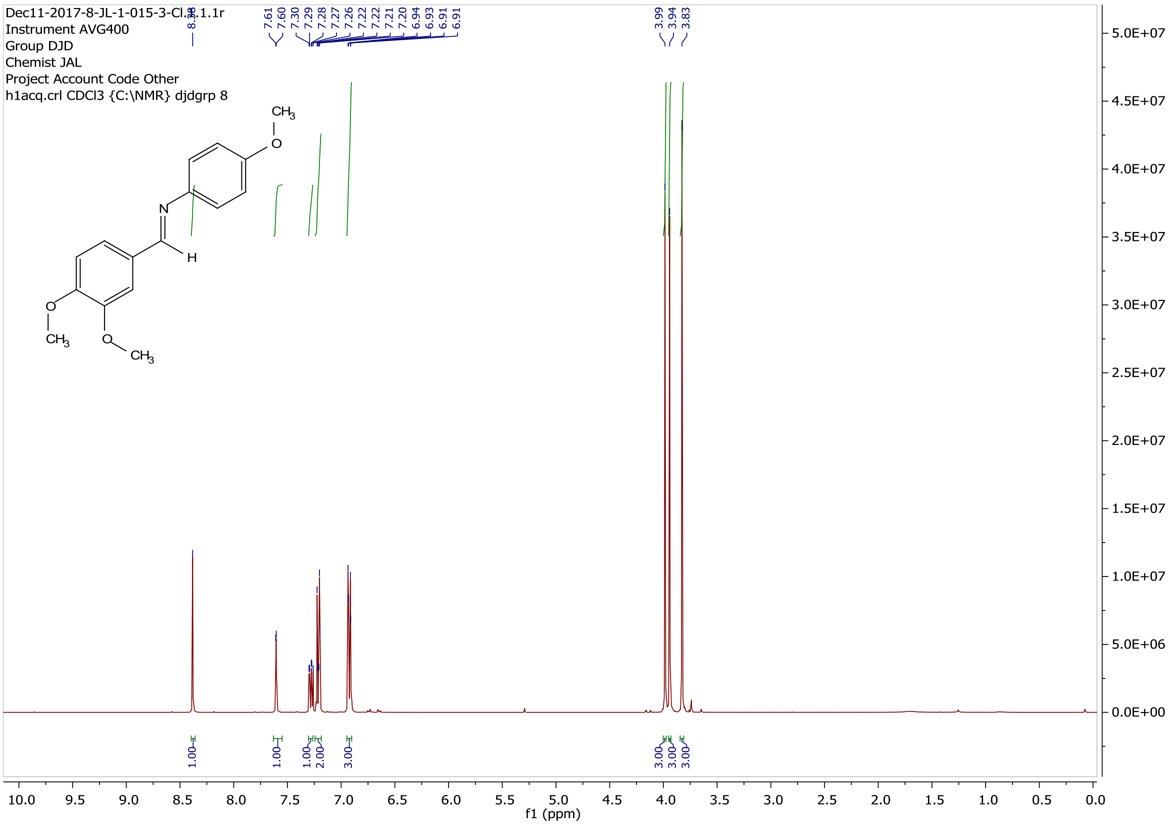

1n – ^{13}C NMR (100 MHz, $CDCl_3$)

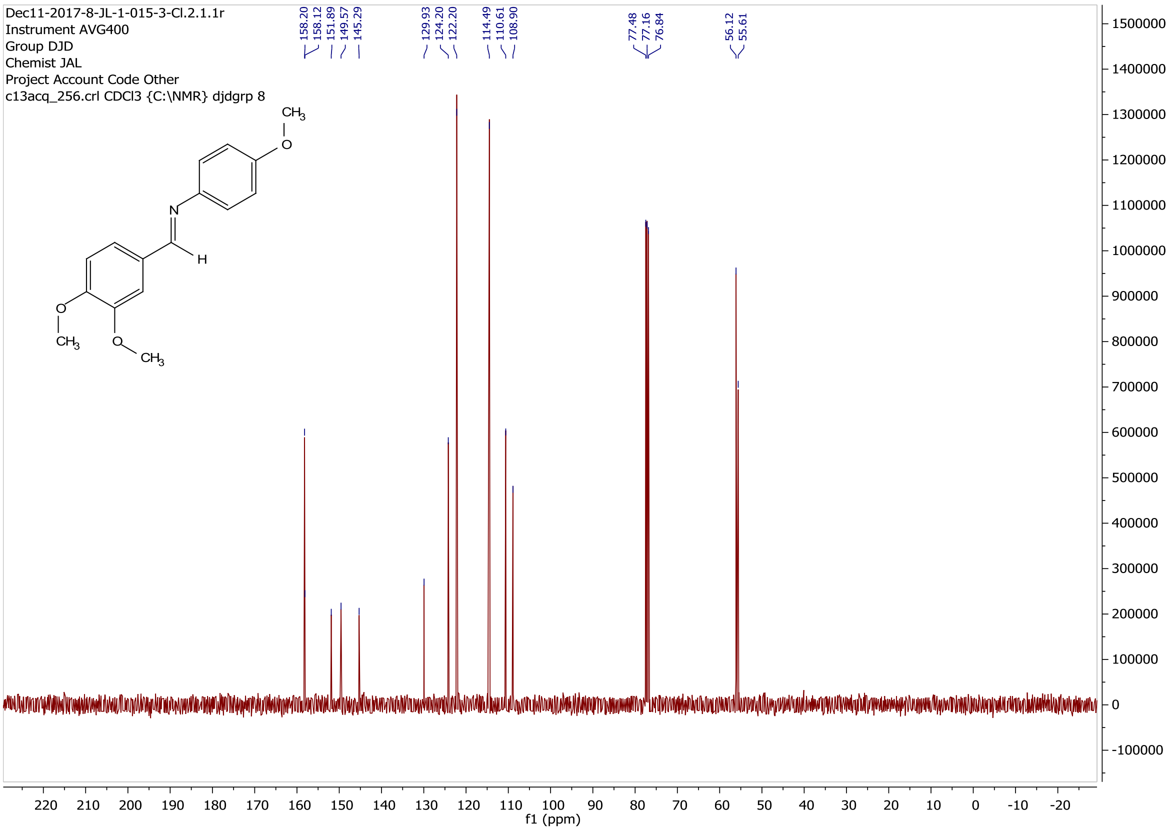

1o – ^{1}H NMR (400 MHz, $CDCl_3$)

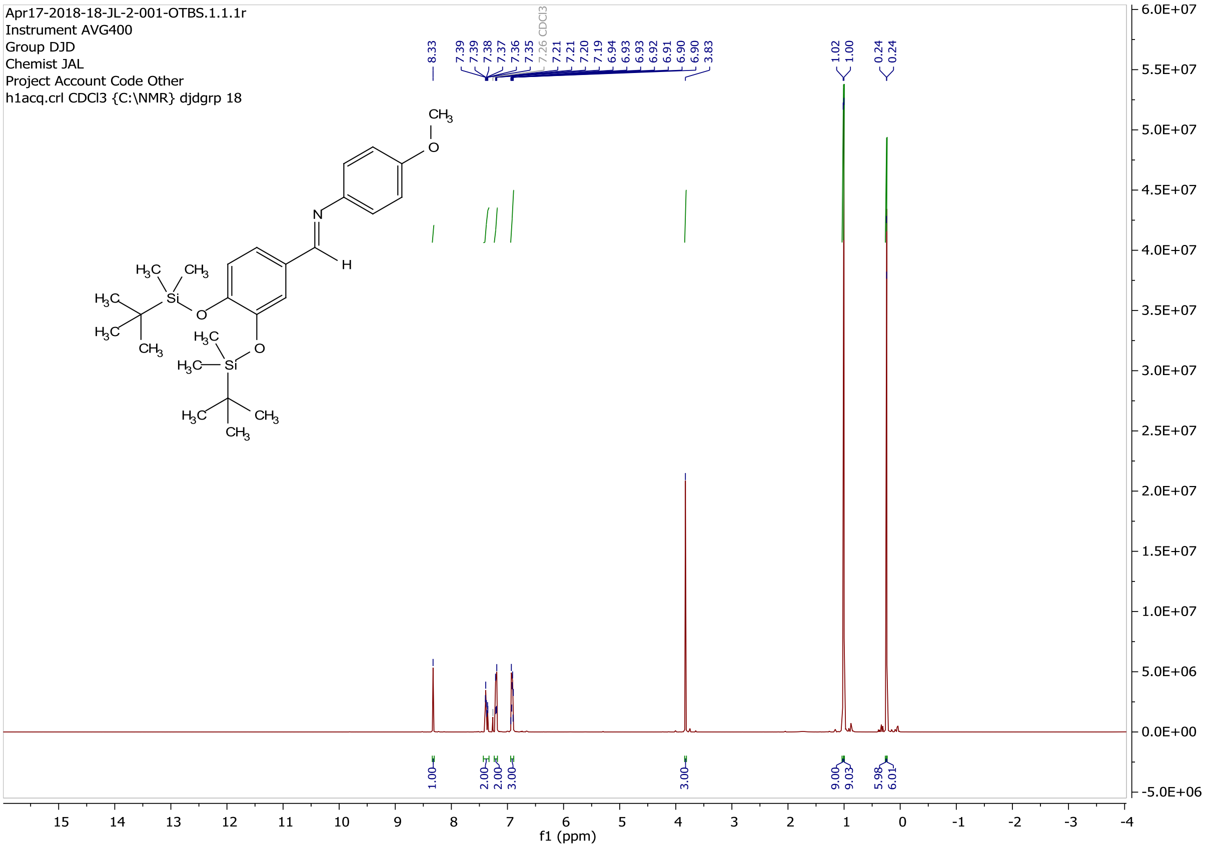

1o – ^{13}C NMR (126 MHz, $CDCl_3$)

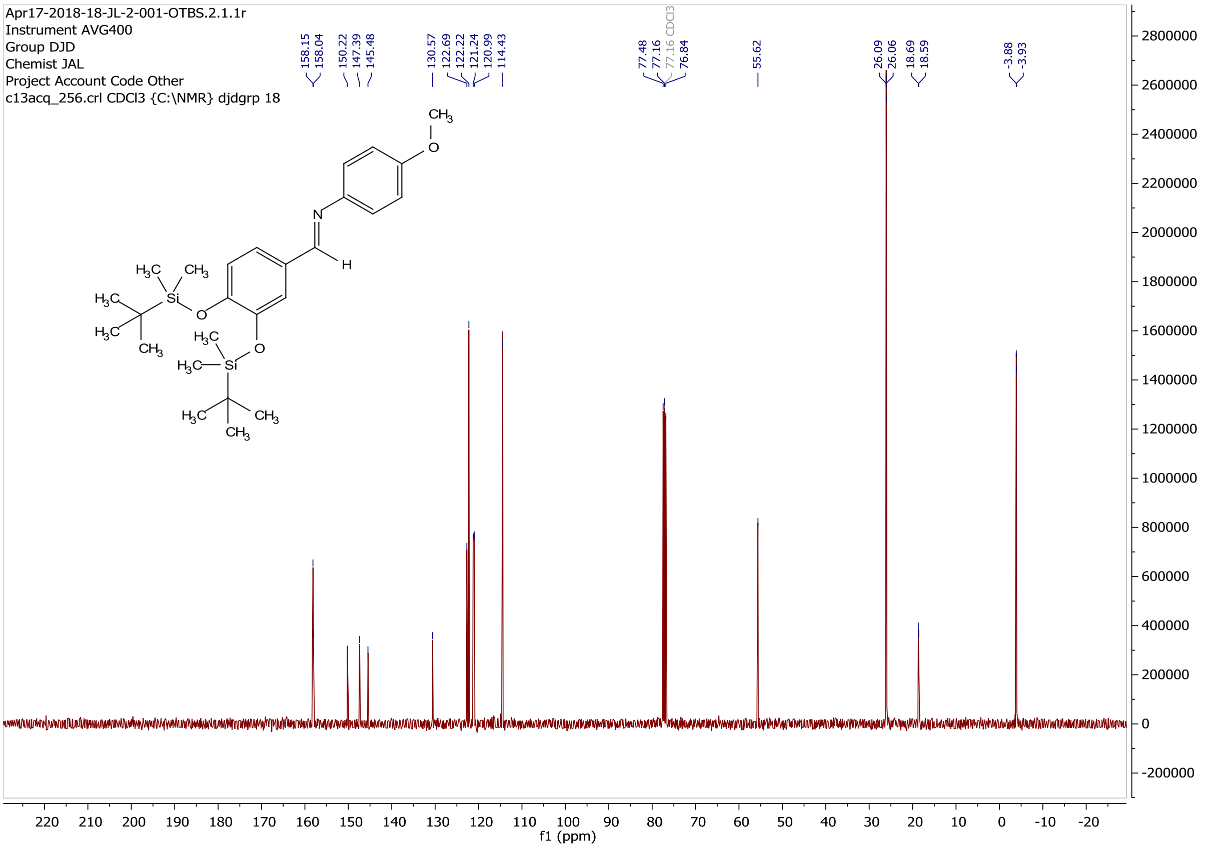

1s – ^{1}H NMR (400 MHz, $CDCl_3$)

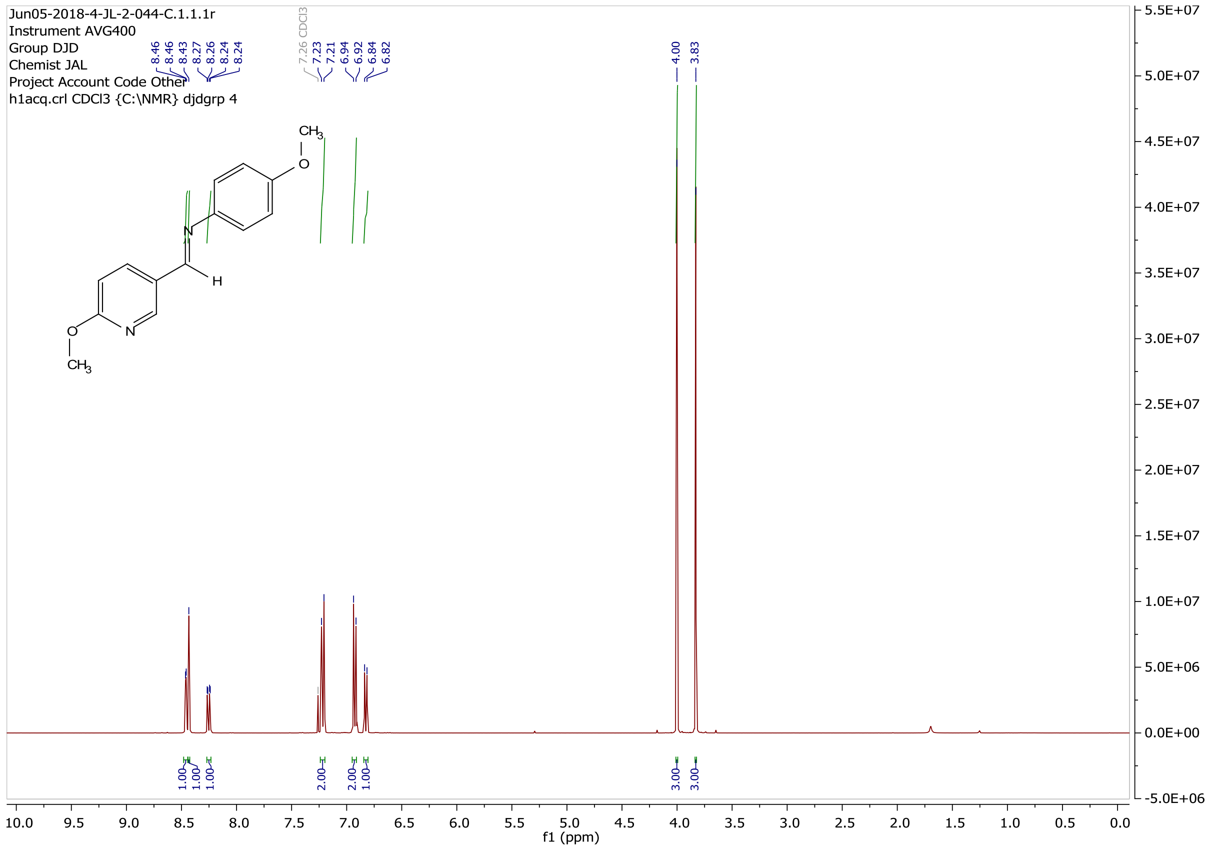

1s – ^{13}C NMR (126 MHz, $CDCl_3$)

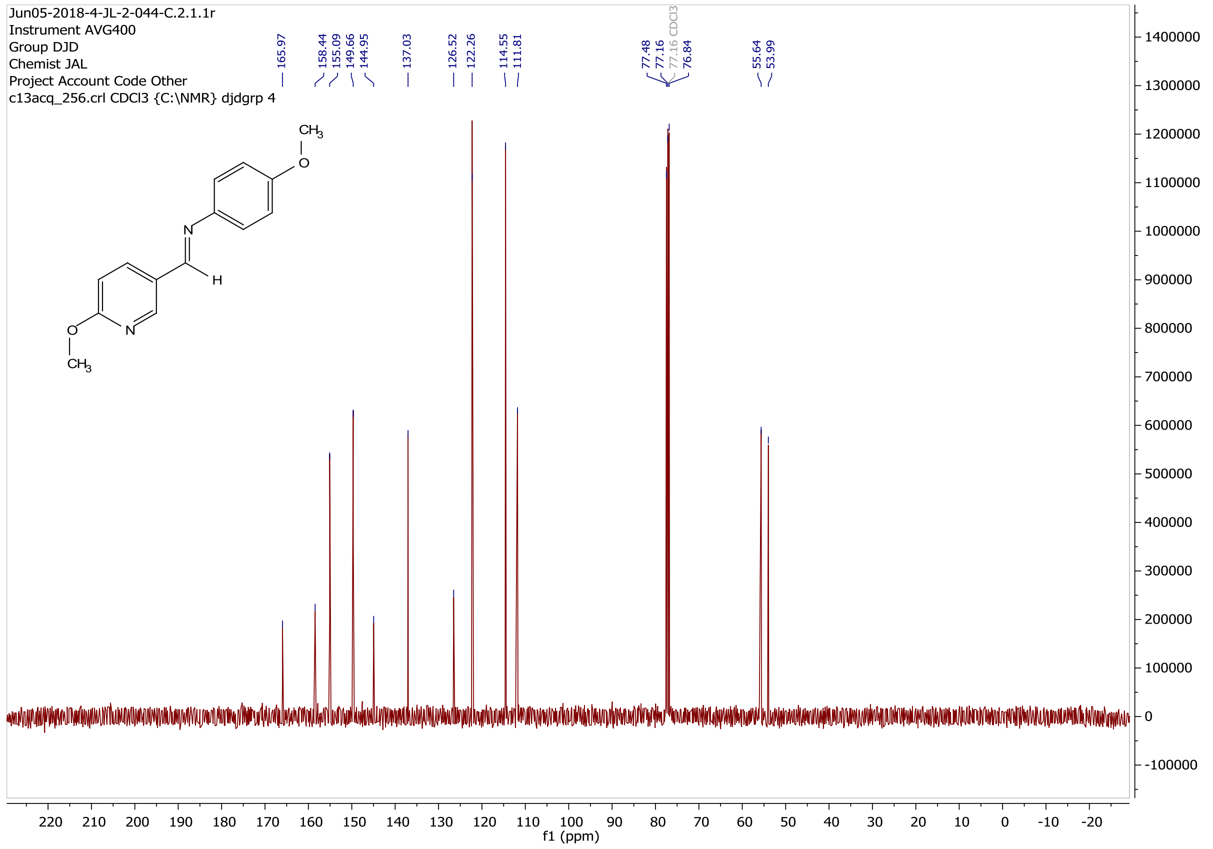

3a – ^{1}H NMR (400 MHz, $CDCl_3$)

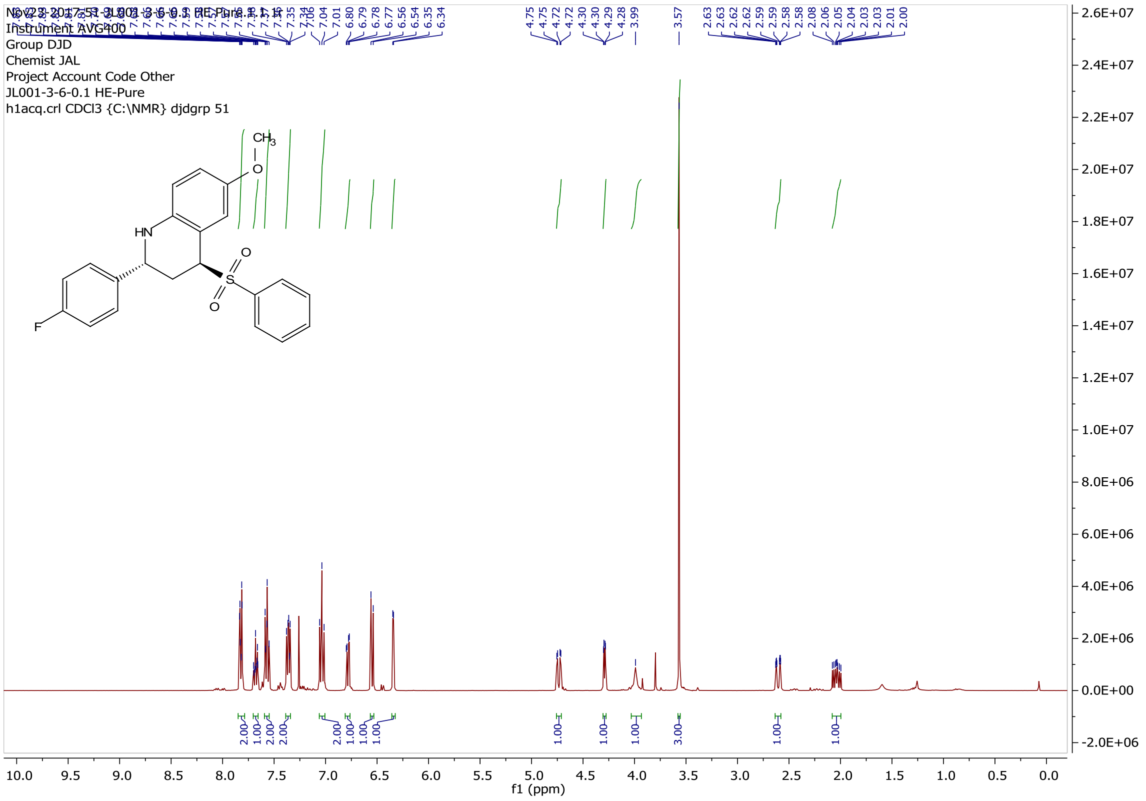

3a – ^{19}F NMR (377 MHz, $CDCl_3$)

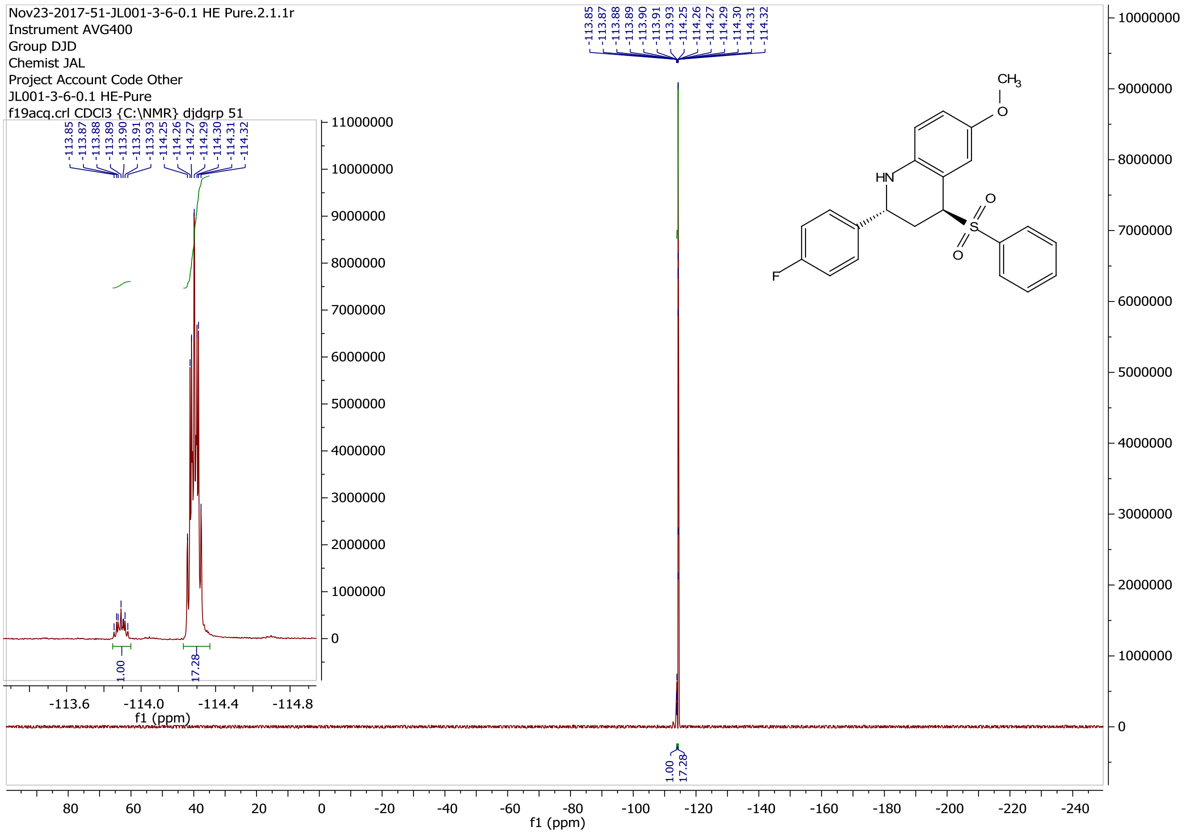

3a – ^{13}C NMR (100 MHz, $CDCl_3$)

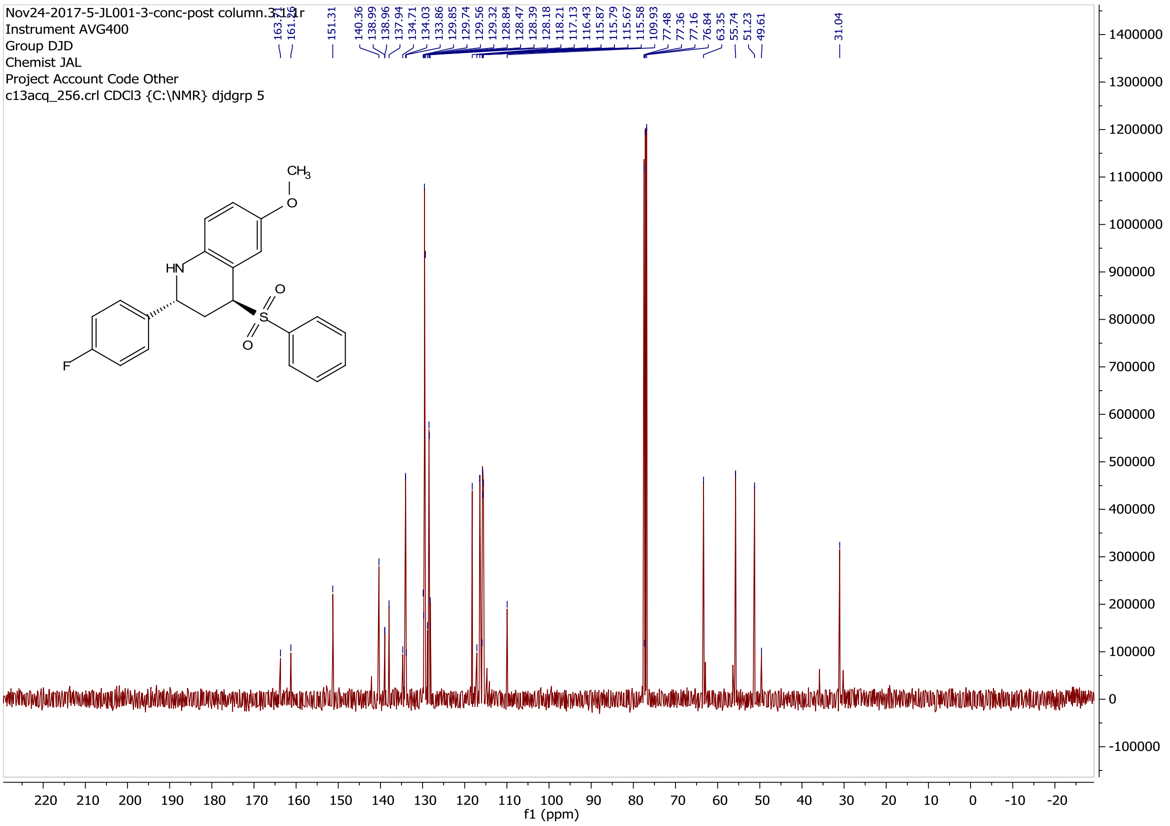

3b – ^{1}H NMR (400 MHz, DMSO-d_6)

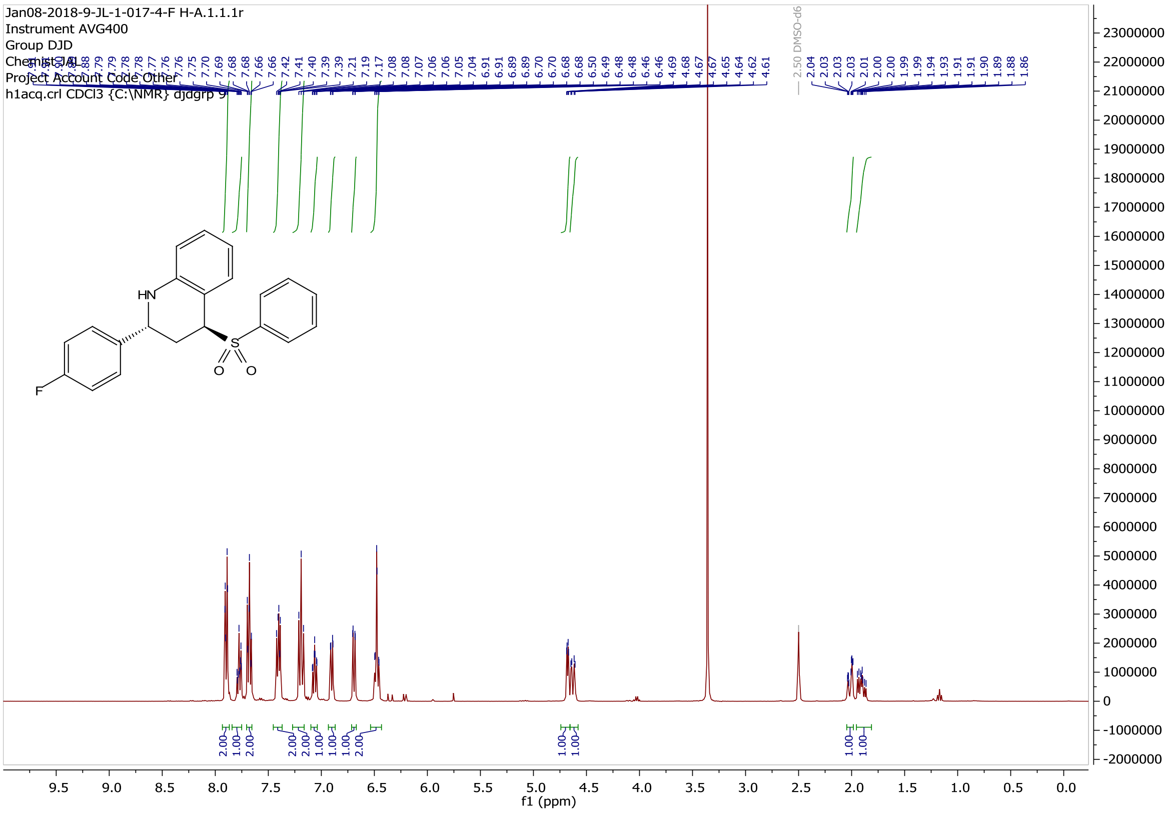

3b – ^{19}F NMR (377 MHz, DMSO-d_6)

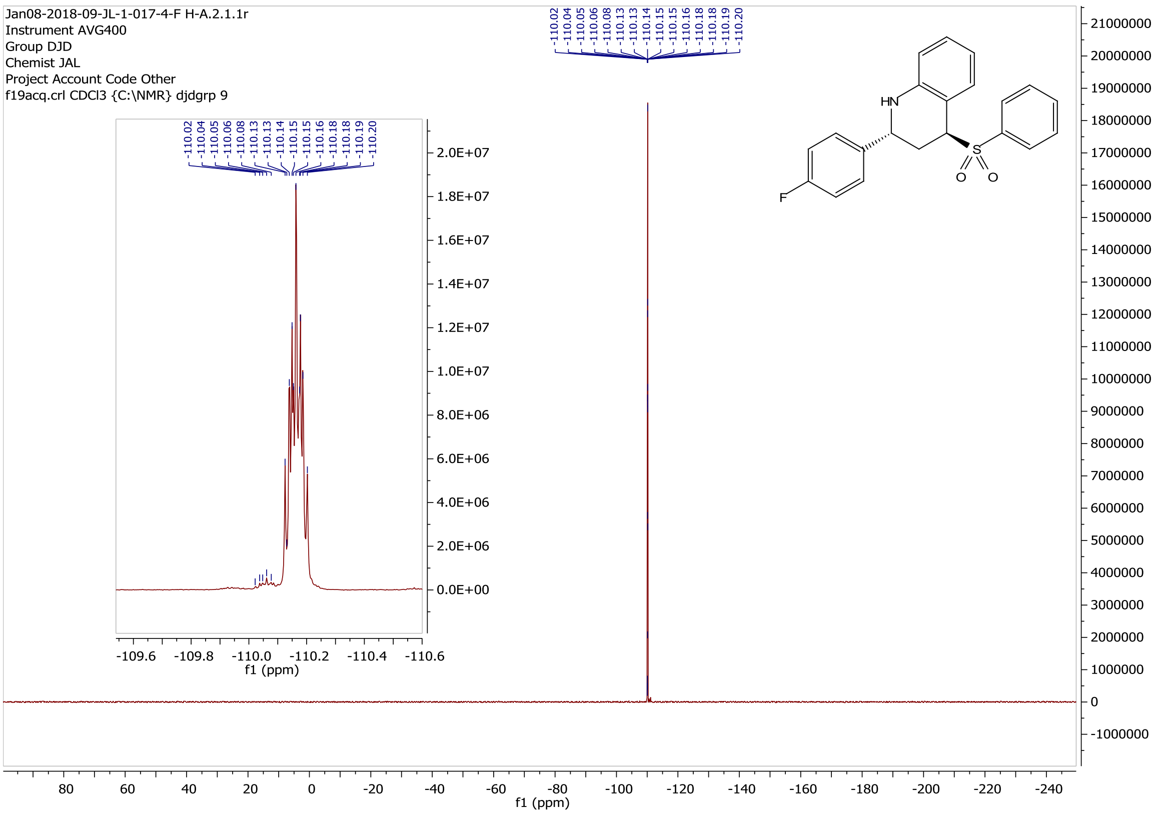

3b – ^{13}C NMR (100 MHz, DMSO-d_6)

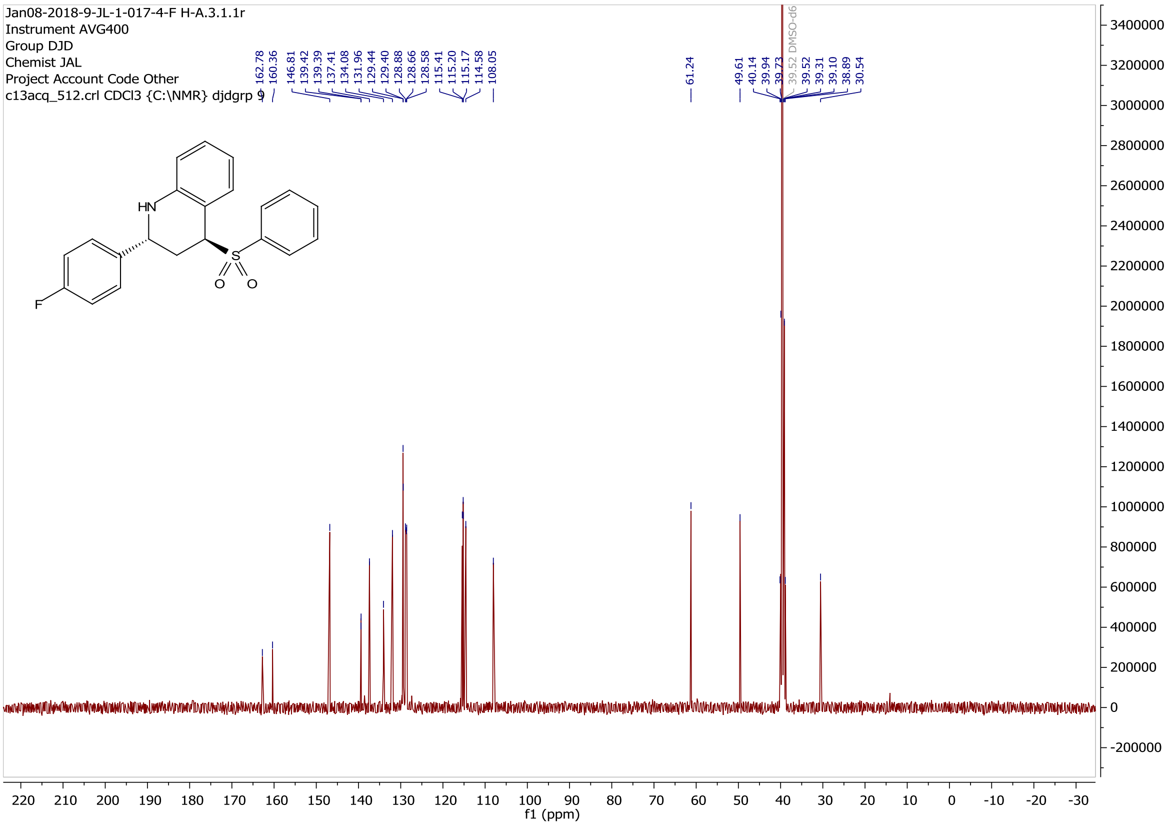

3c – ^{1}H NMR (400 MHz, DMSO-d_6)

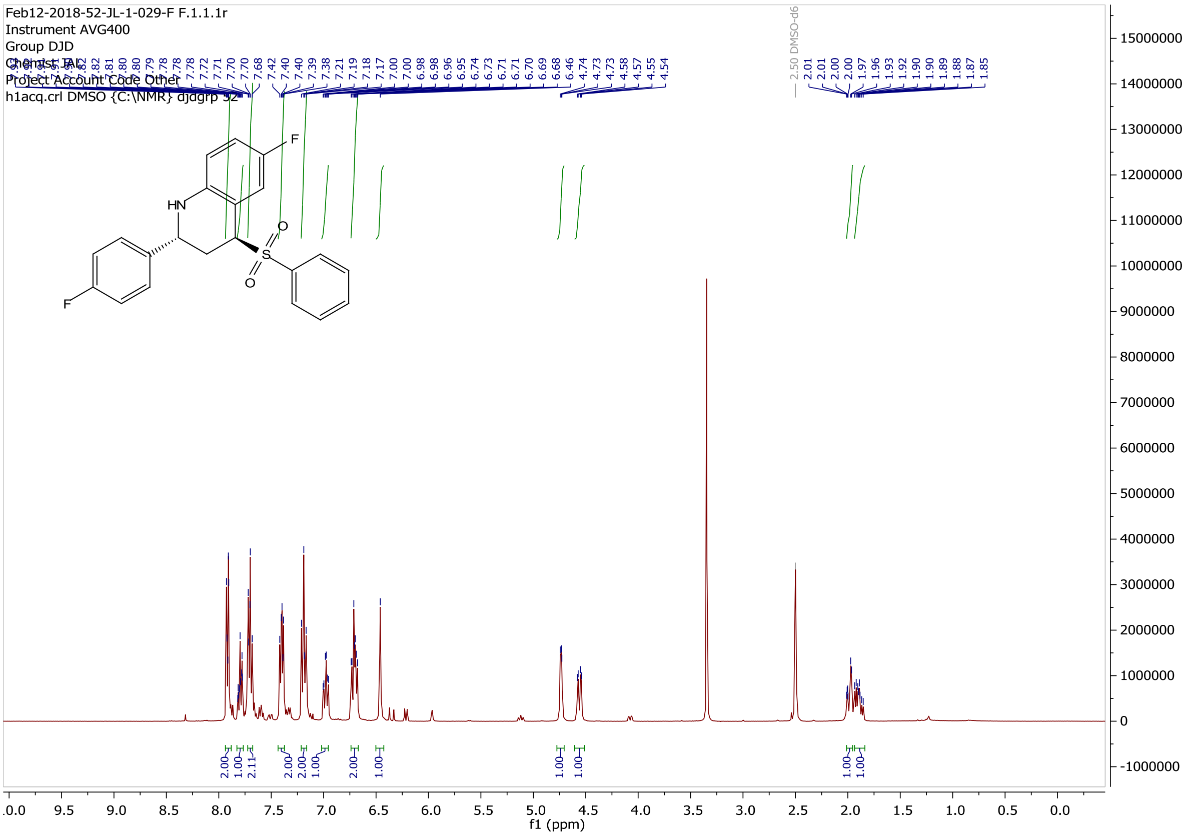

3c – ^{19}F NMR (377 MHz, $CDCl_3$)

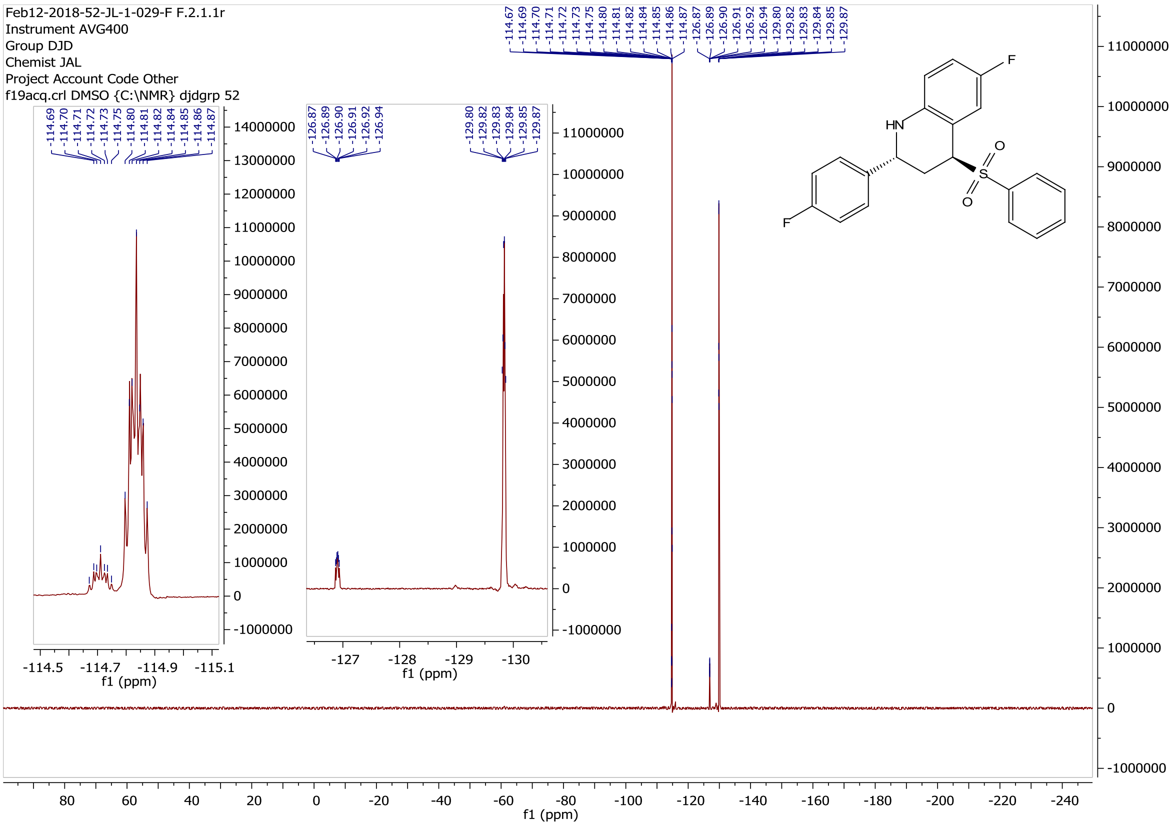

3c – ^{13}C NMR (DMSO-d_6)

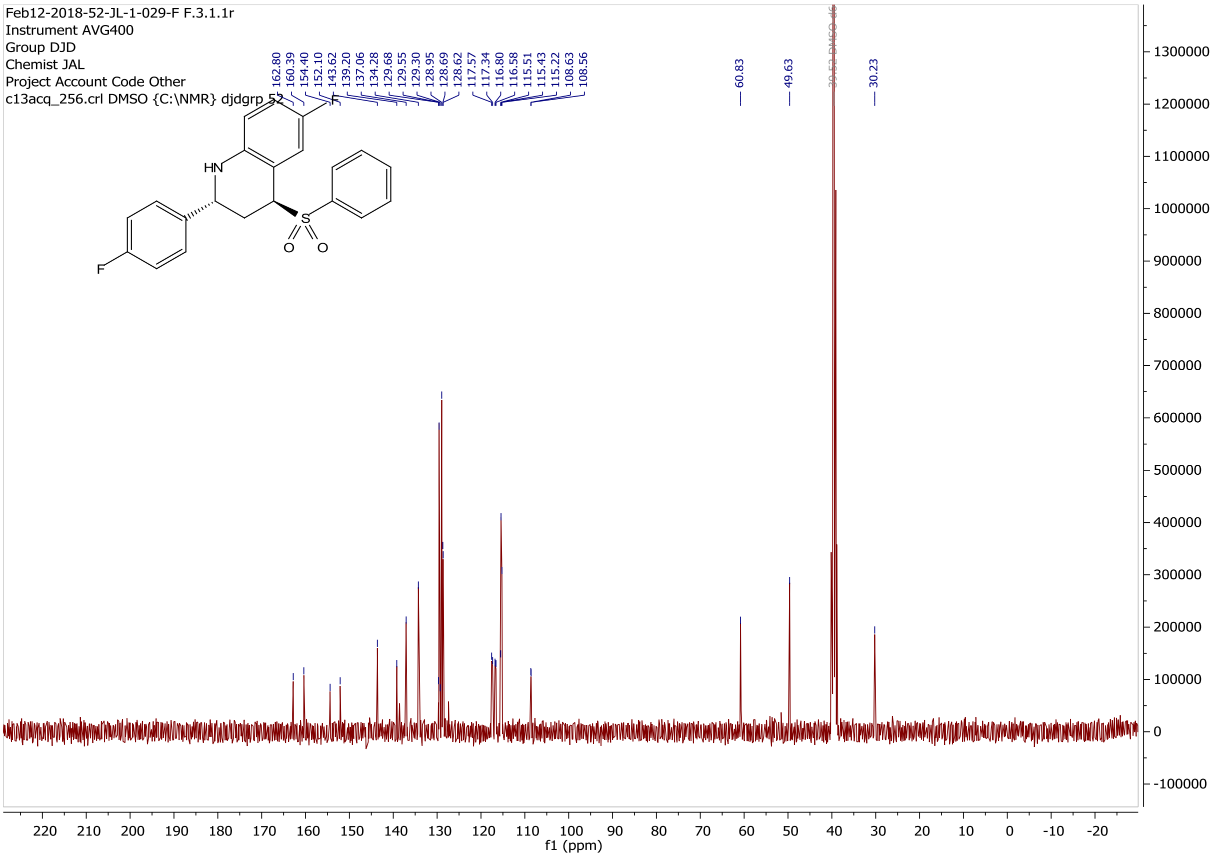

3d – ^{1}H NMR (400 MHz, DMSO-d_6:CDCl$_3$ 1:1)

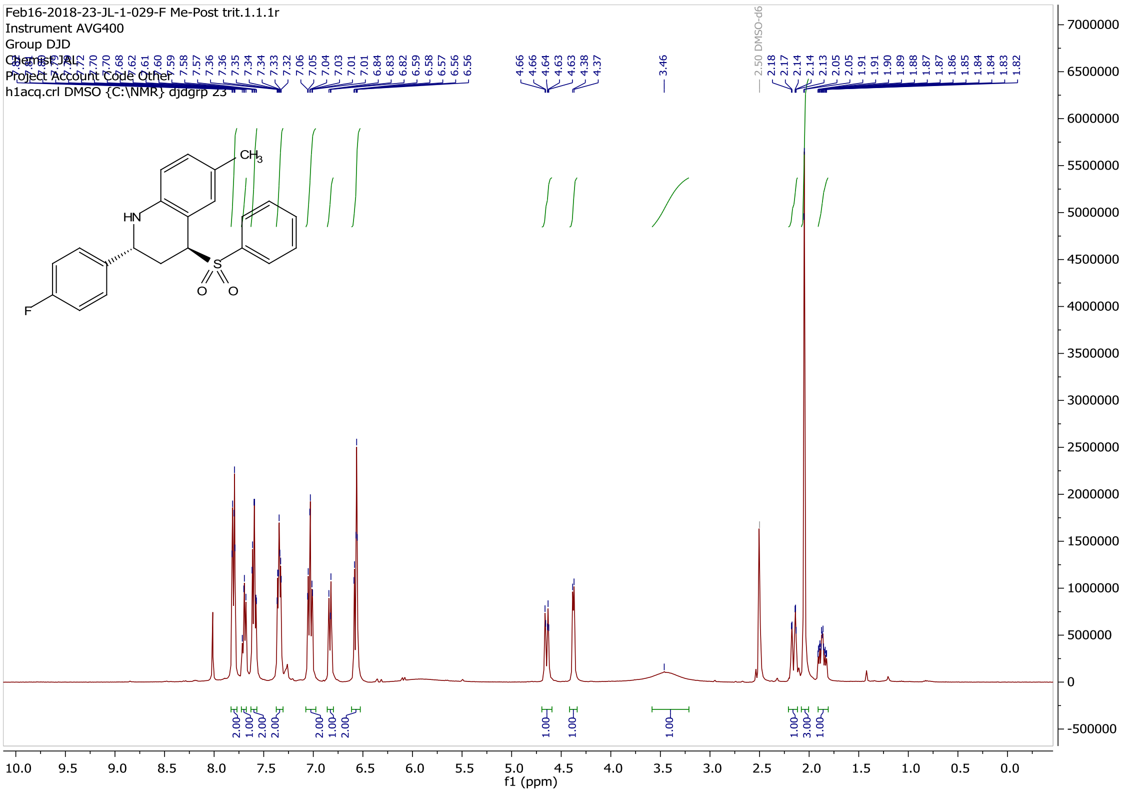

3d – ^{19}F NMR (377 MHz, DMSO-d_6:CDCl$_3$ 1:1)

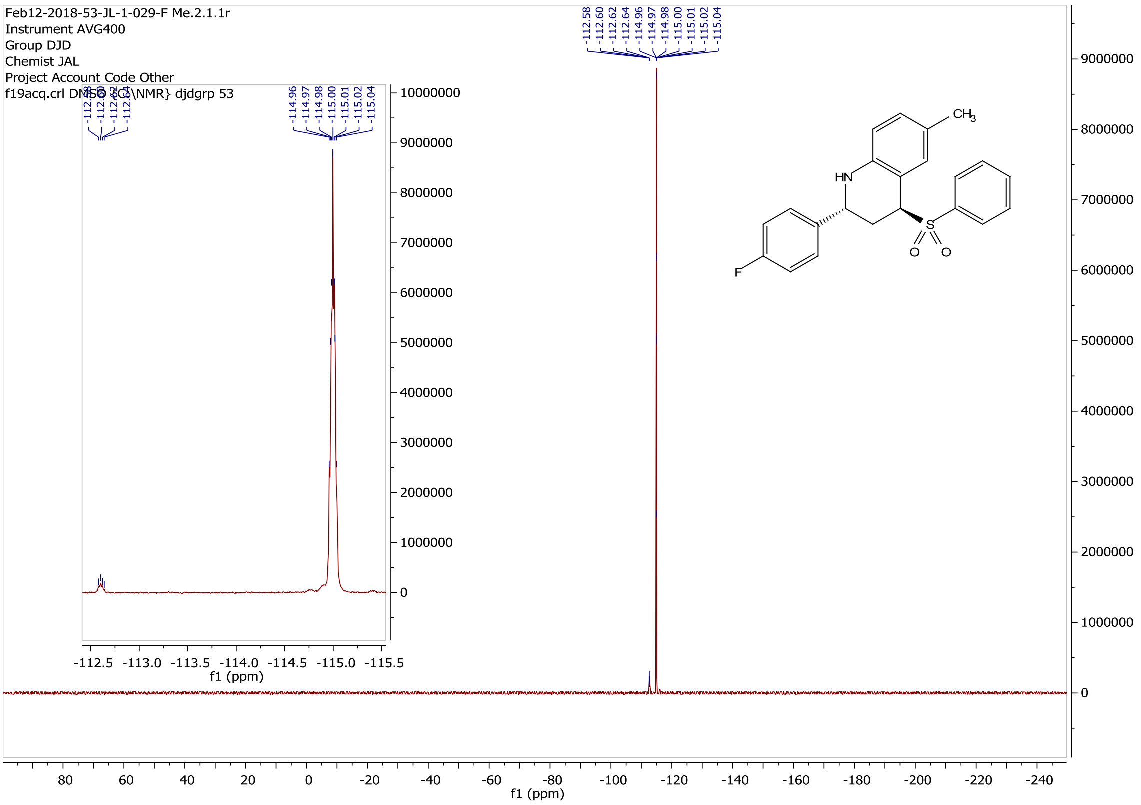

3d – ^{13}C NMR (100 MHz, DMSO-d_6:CDCl$_3$ 1:1)

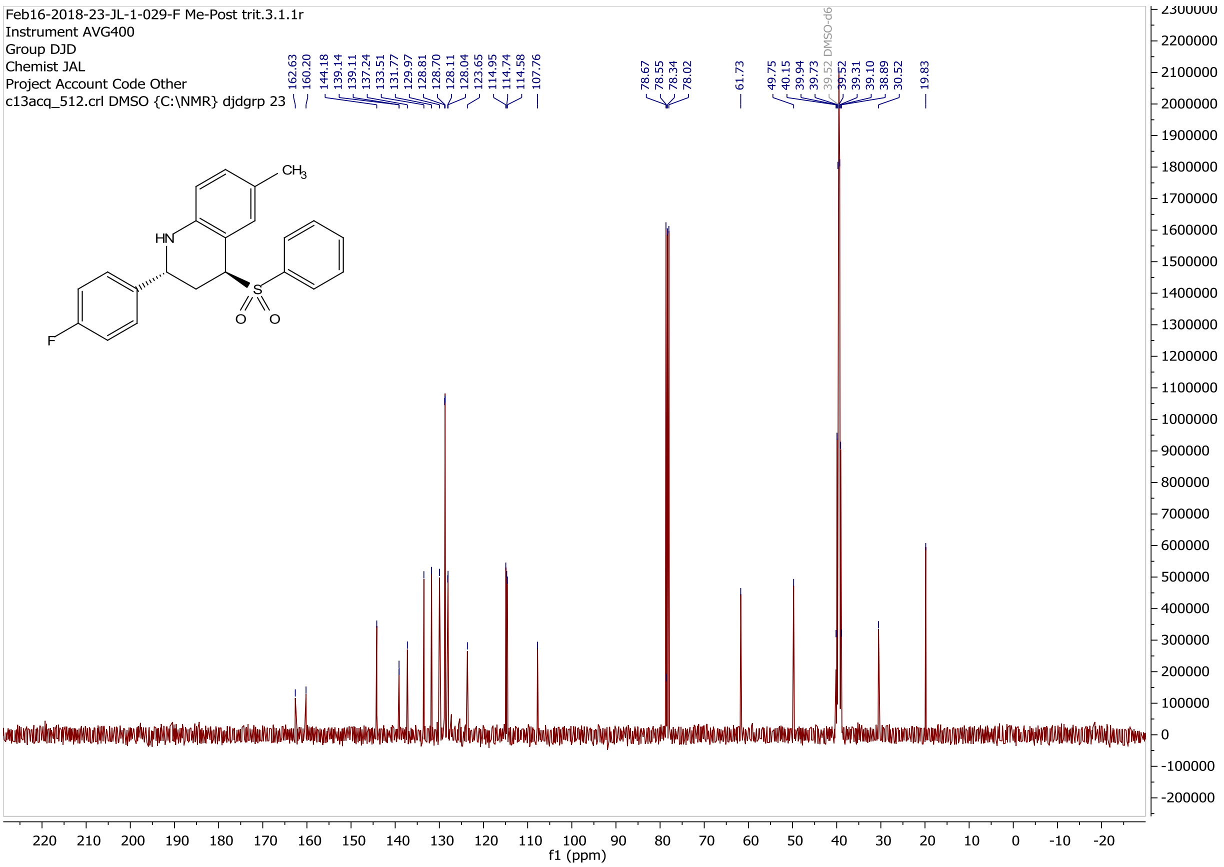

3e – ^{1}H NMR (400 MHz, DMSO-d_6)

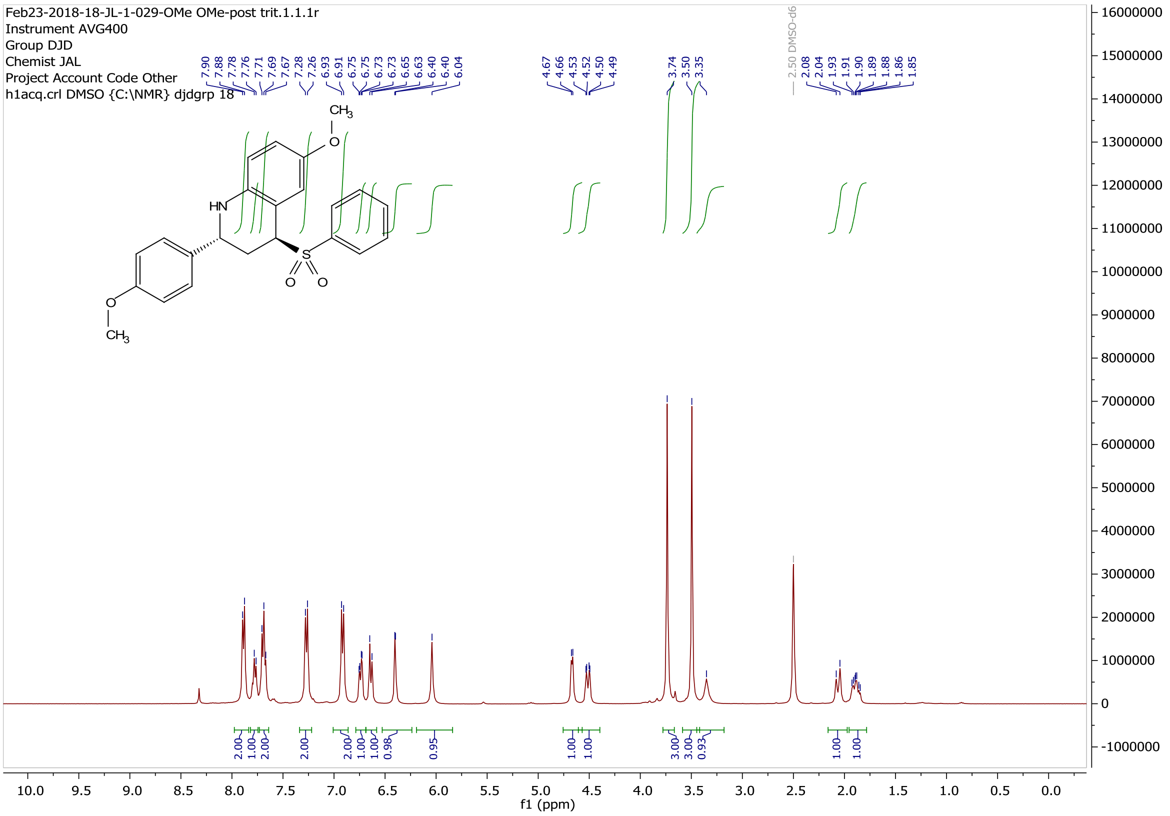

3e – ^{13}C NMR (100 MHz, DMSO-d_6)

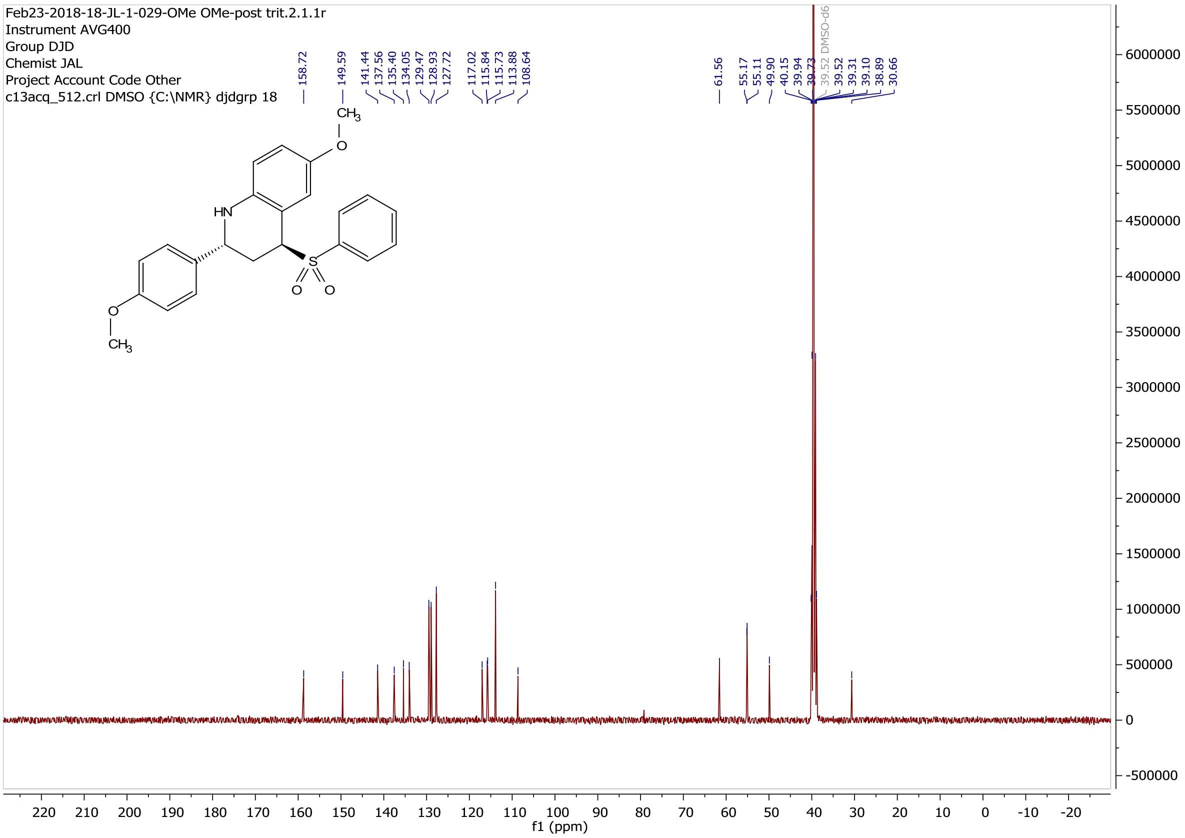

3f – ^{1}H NMR (400 MHz, $CDCl_3$)

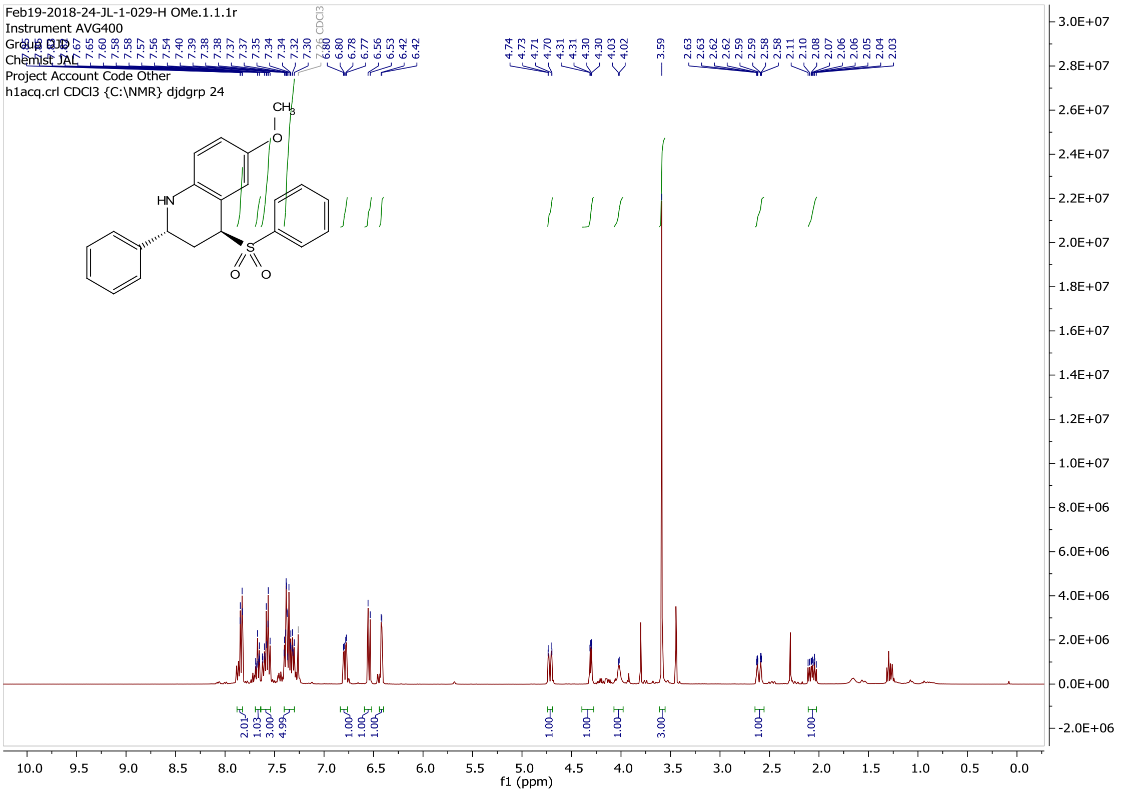

3f – ^{13}C NMR (100 MHz, DMSO-d_6)

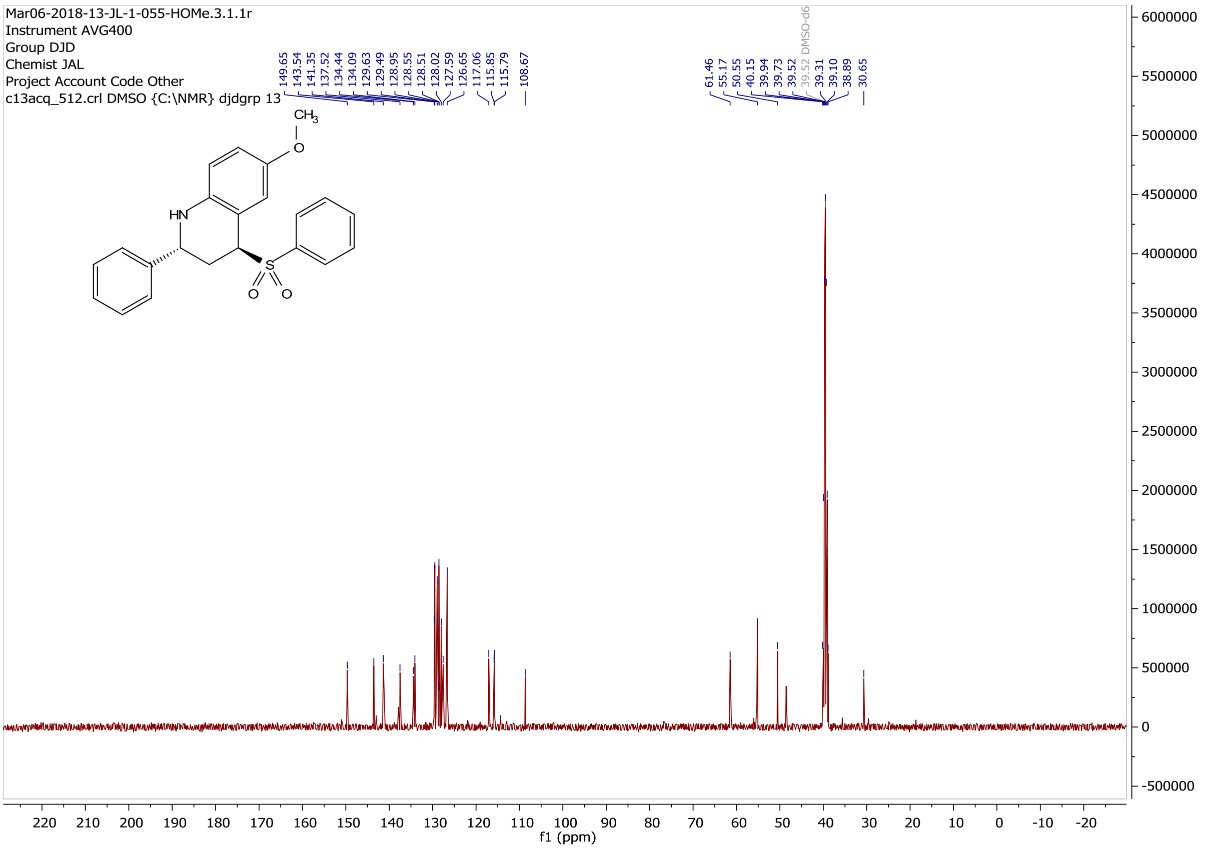

3g – ^{1}H NMR (400 MHz, DMSO-d_6)

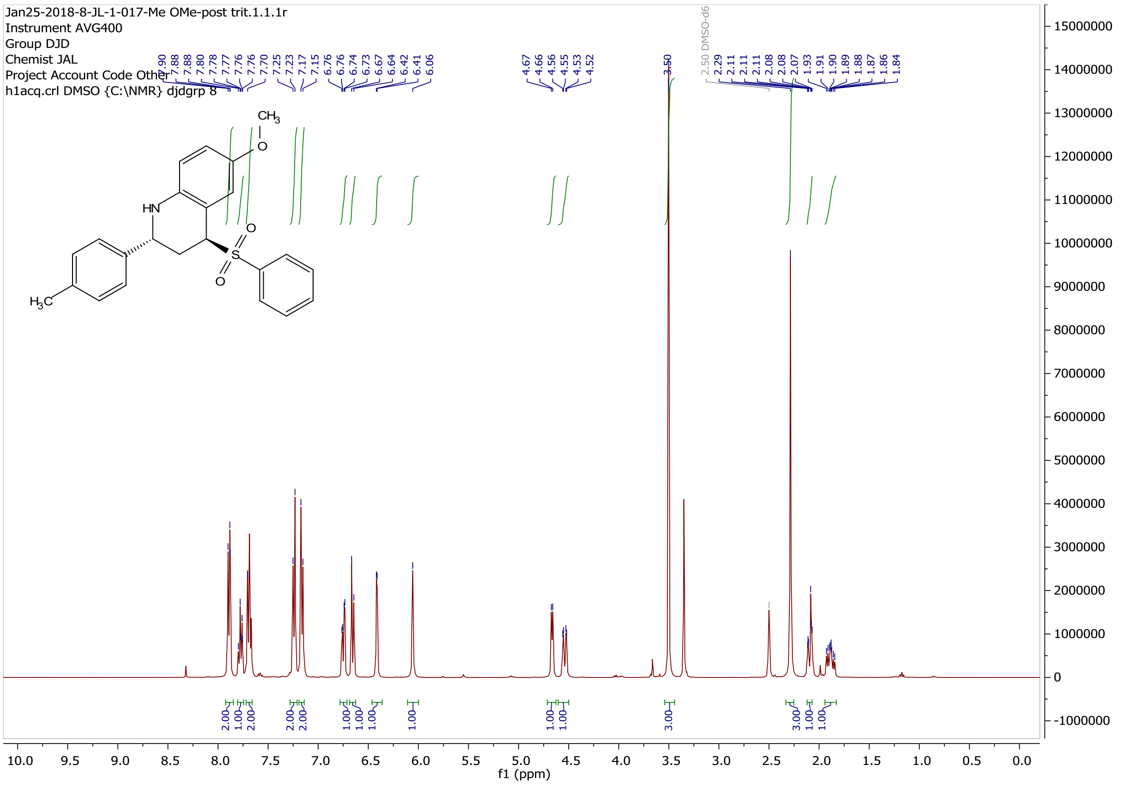

3g – ^{13}C NMR (100 MHz, DMSO-d_6)

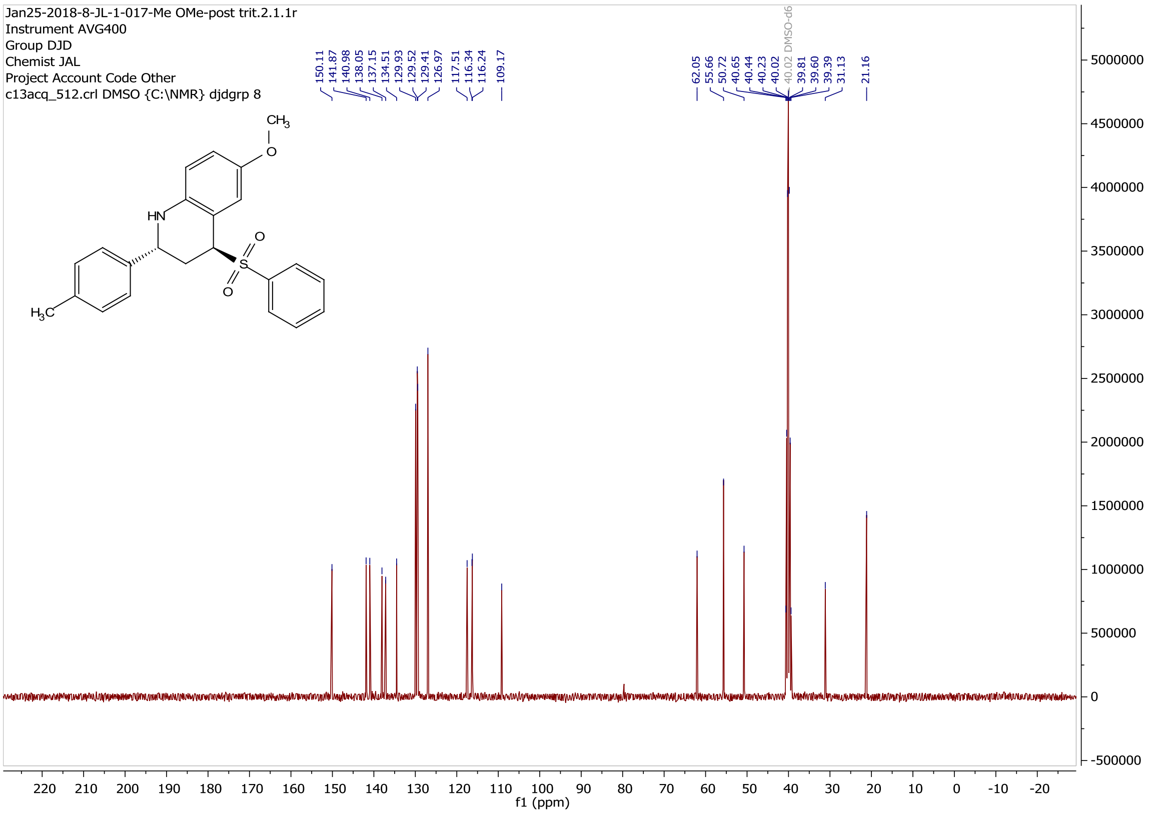

3h – ^{1}H NMR (400 MHz, DMSO-d_6)

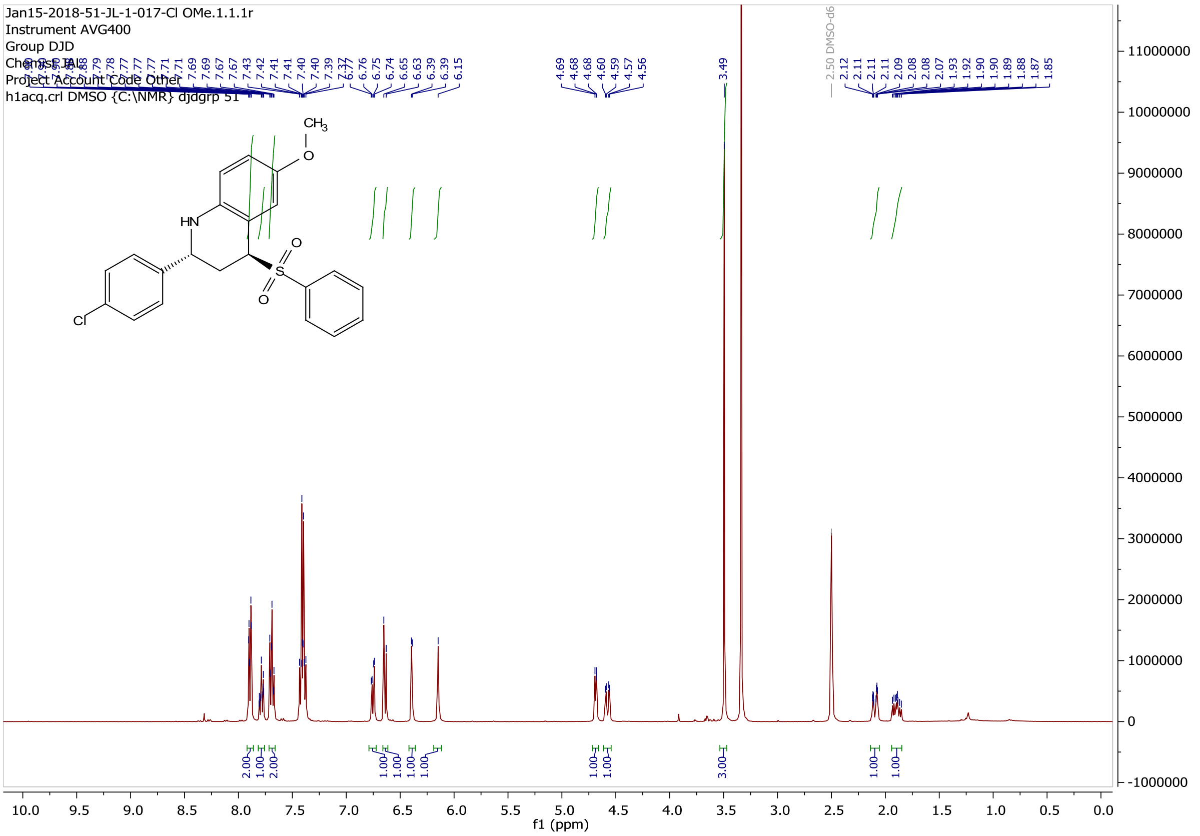

3h – ^{13}C NMR (100 MHz, DMSO-d_6)

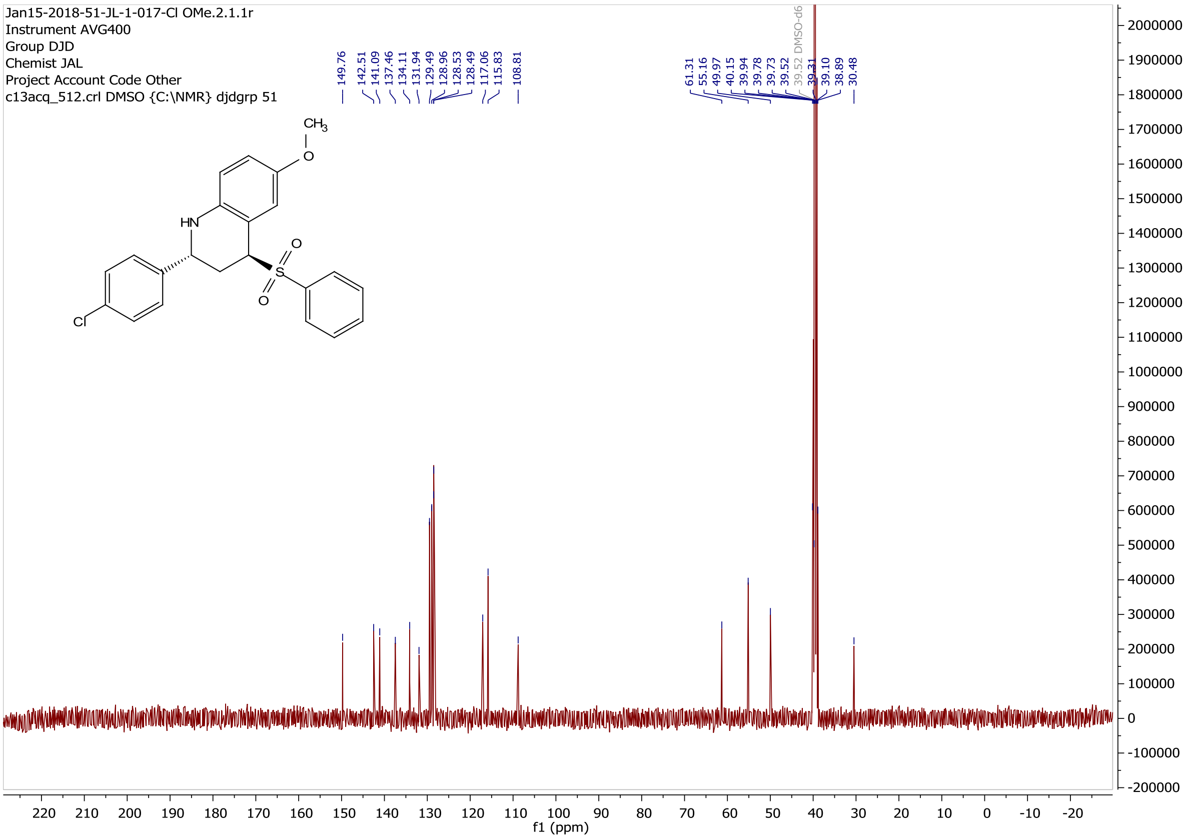

3j – ^{1}H NMR (400 MHz, DMSO-d_6)

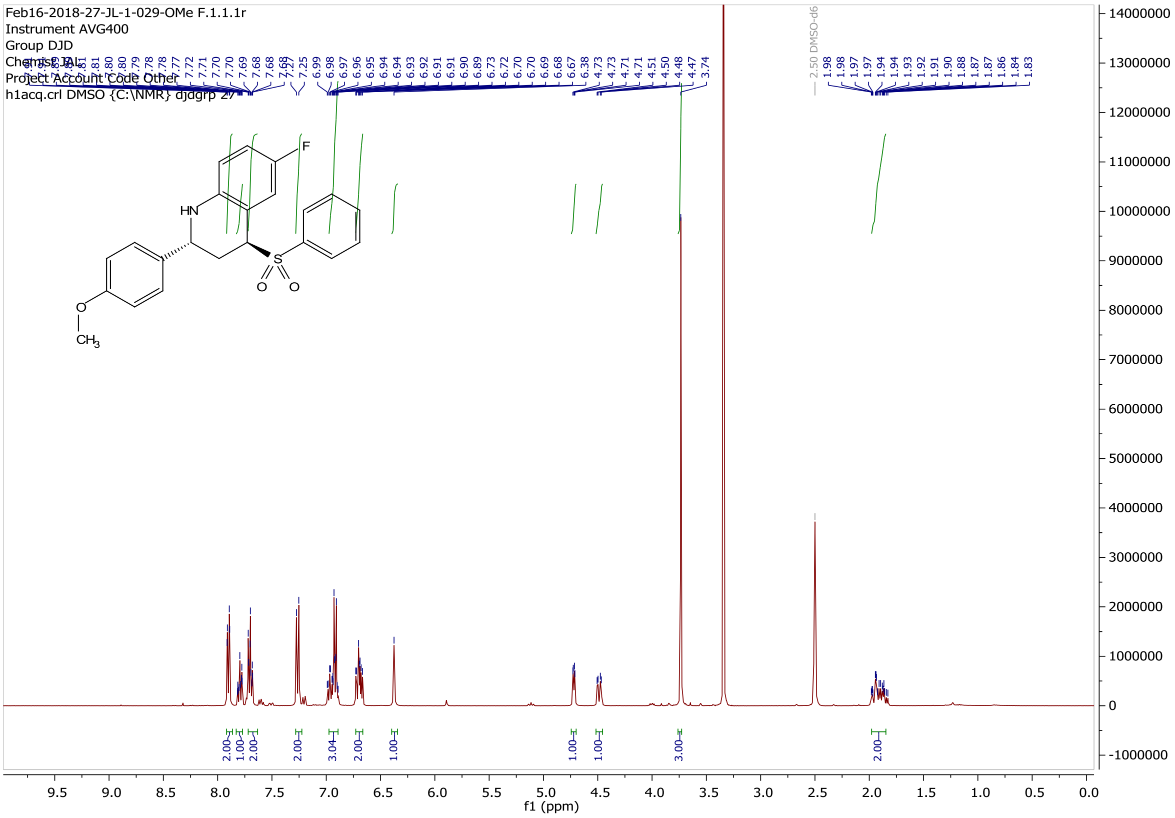

3j – ^{19}F NMR (377 MHz, DMSO-d_6)

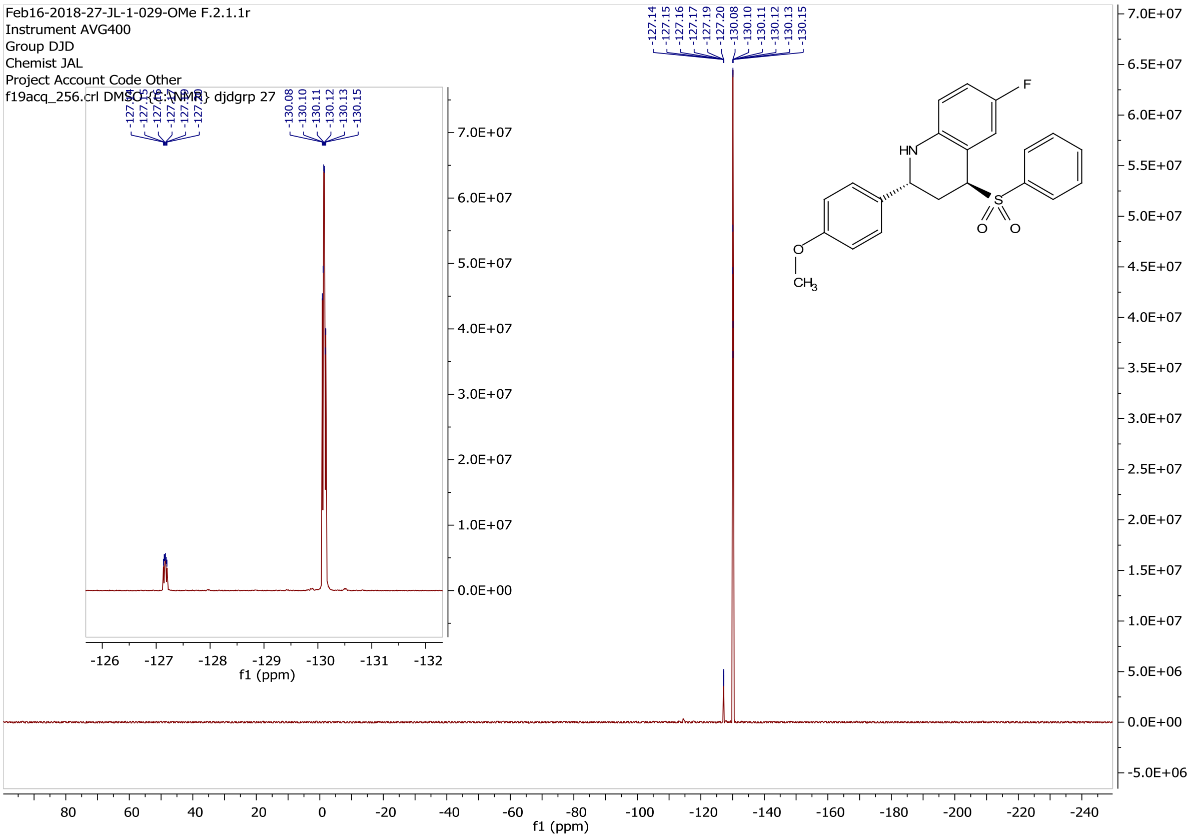

3j – ^{13}C NMR (100 MHz, DMSO-d_6)

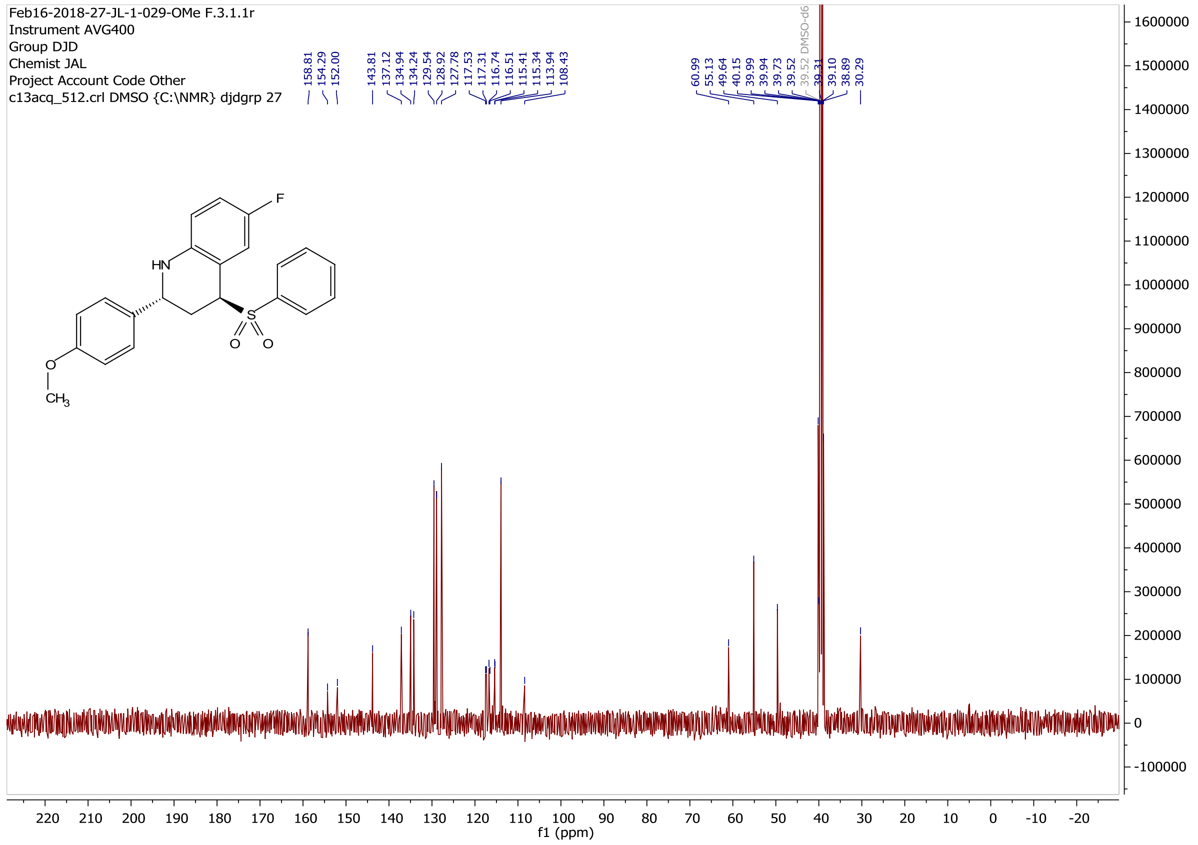

3k – ^{1}H NMR (400 MHz, DMSO-d_6)

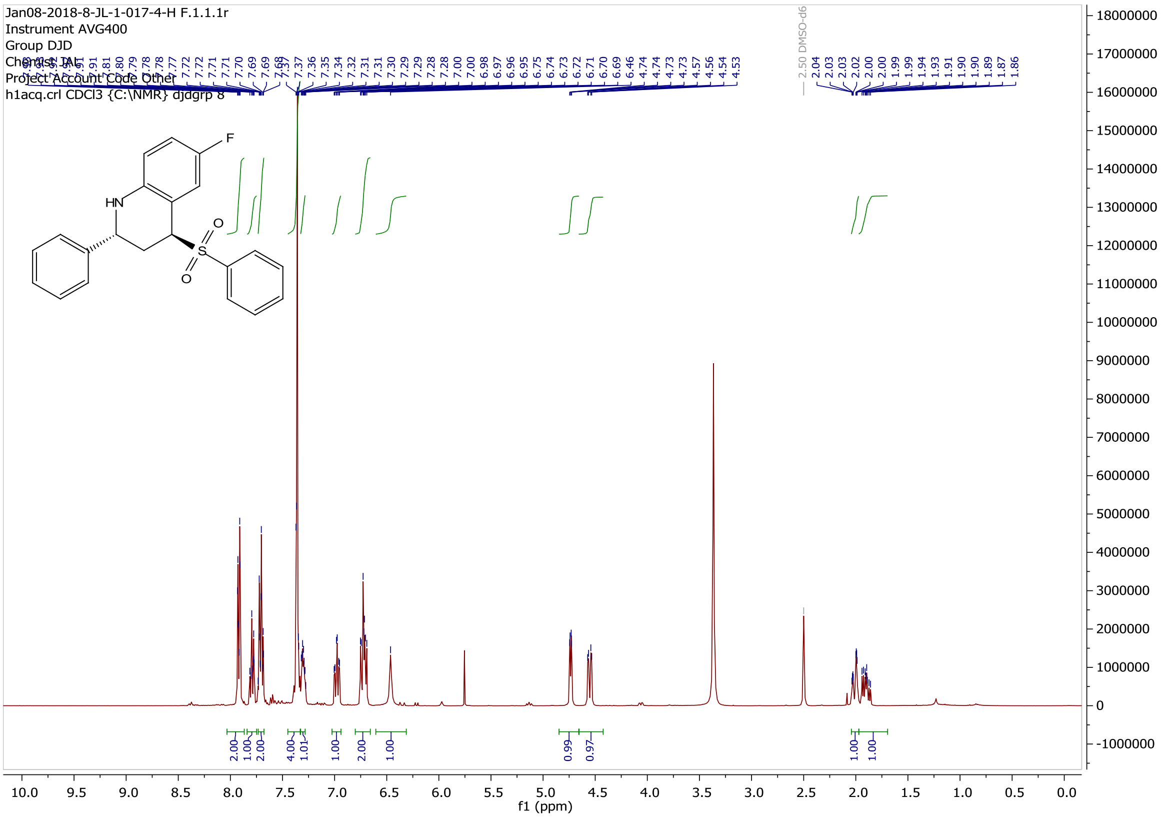

3k – ^{19}F NMR (377 MHz, DMSO-d_6)

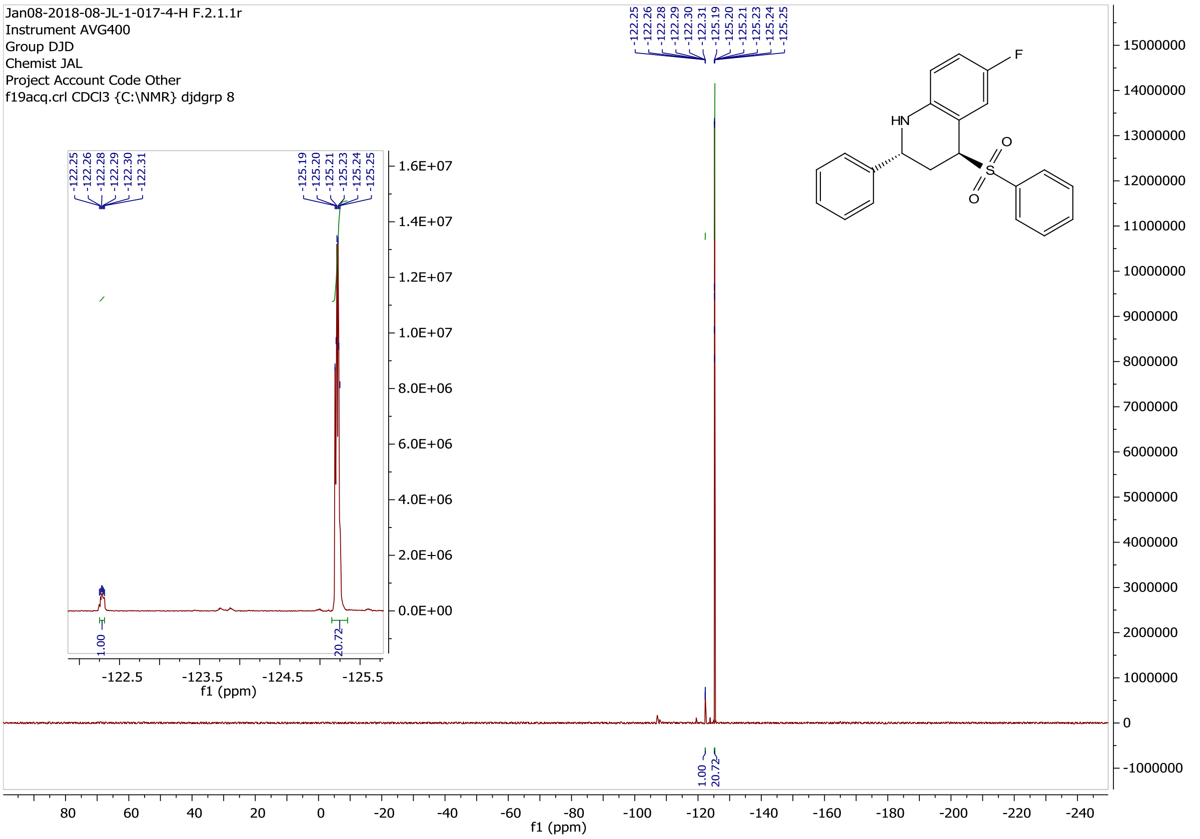

3k – ^{13}C NMR (100 MHz, DMSO-d_6)

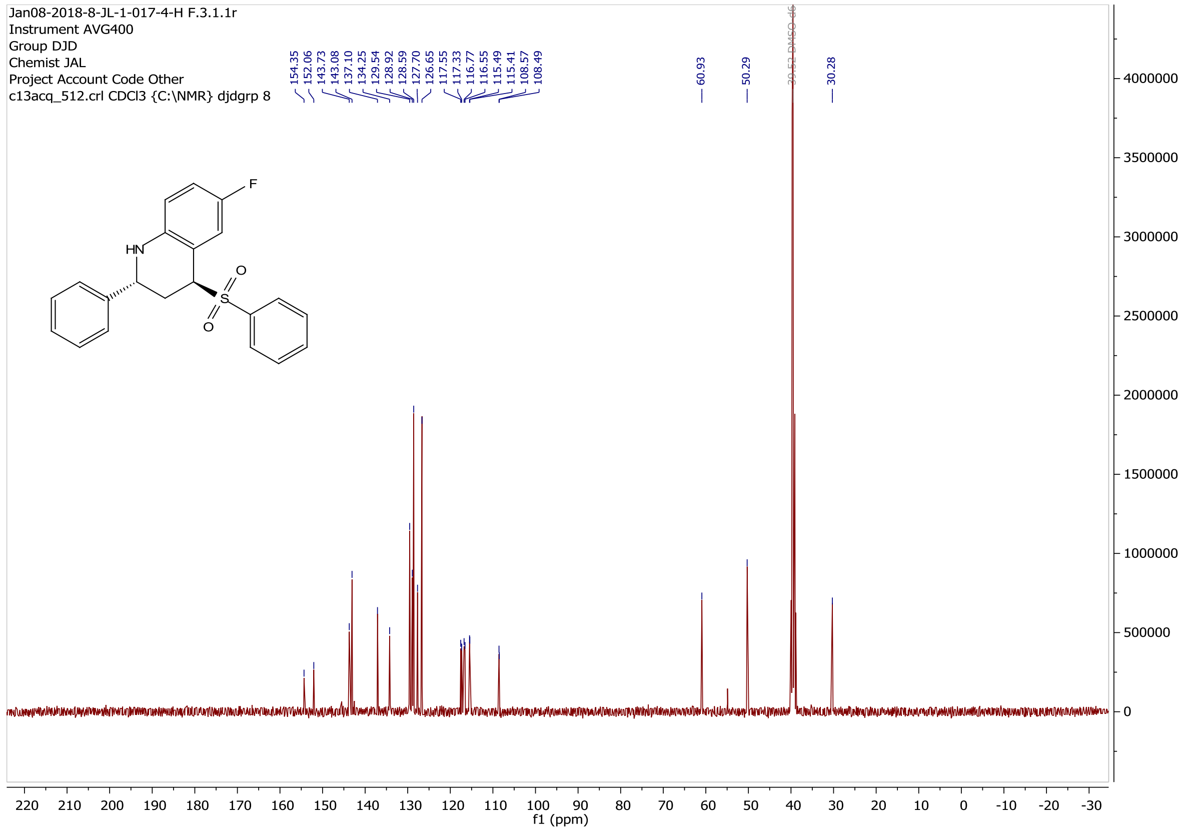

3l – ^{1}H NMR (400 MHz, $CDCl_3$)

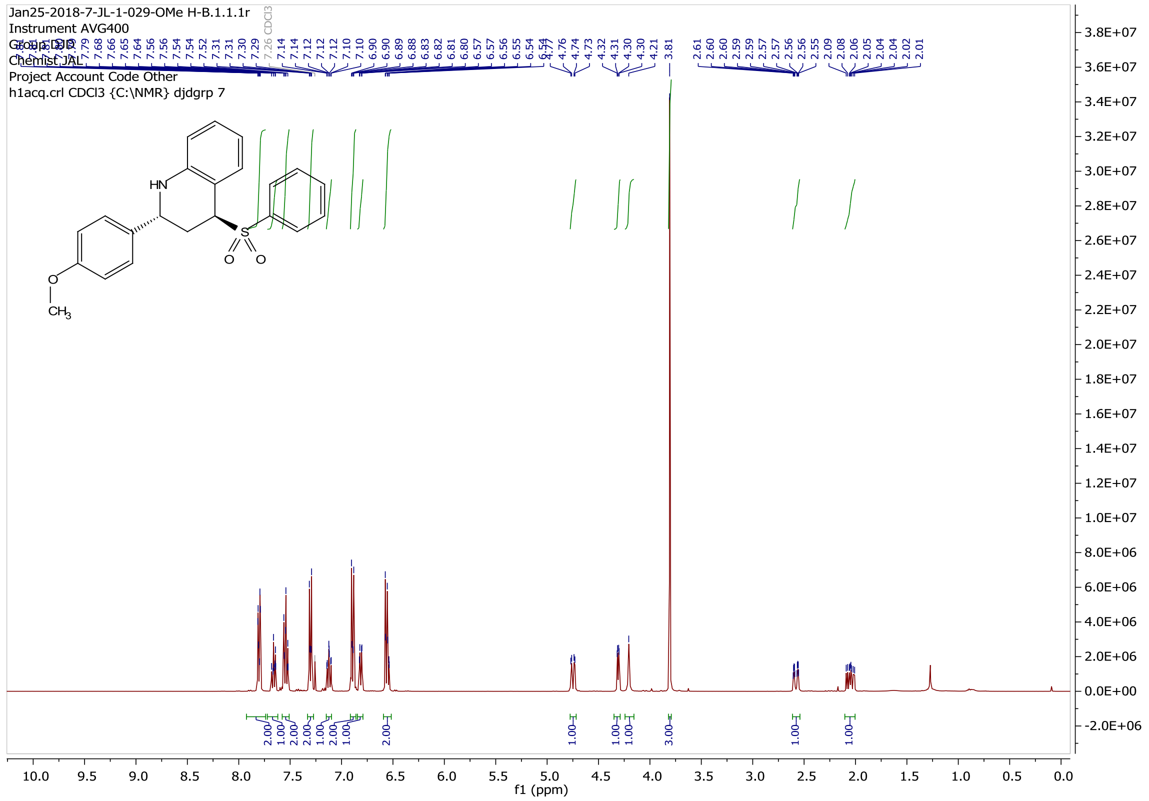

3l – ^{13}C NMR (100 MHz, $CDCl_3$)

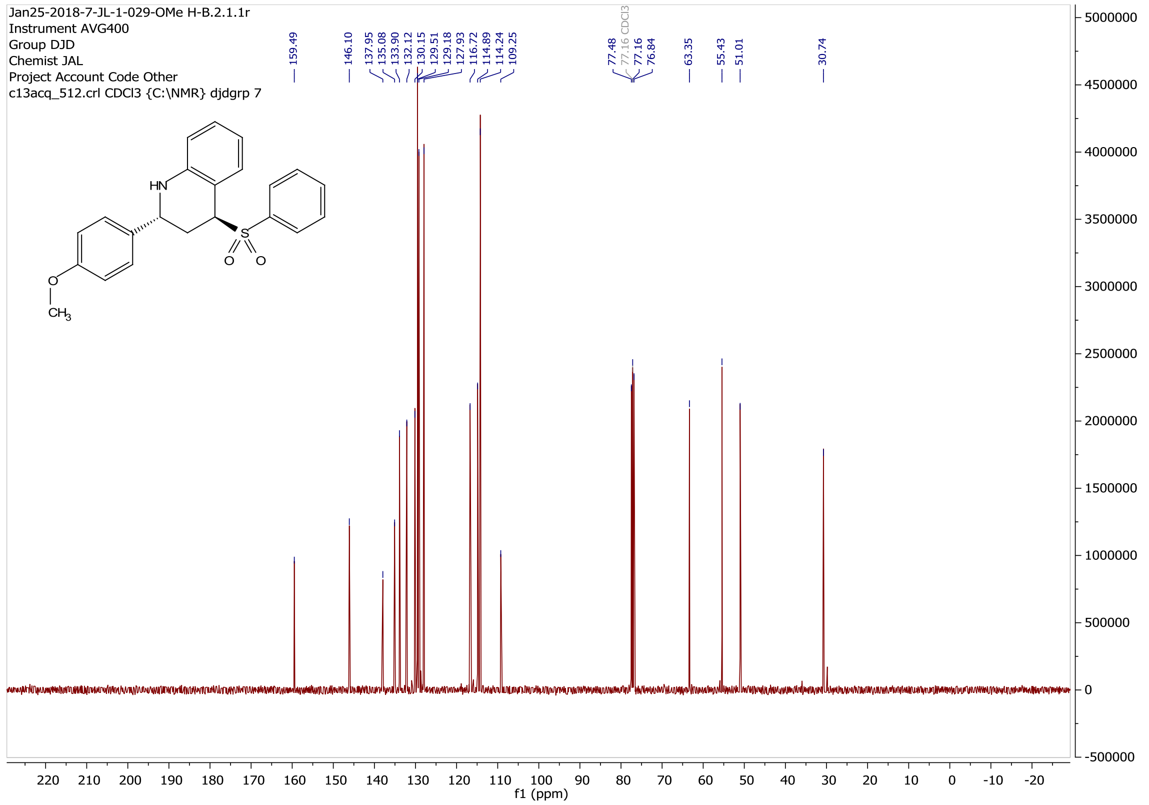

3m – ^{1}H NMR (400 MHz, DMSO-d_6)

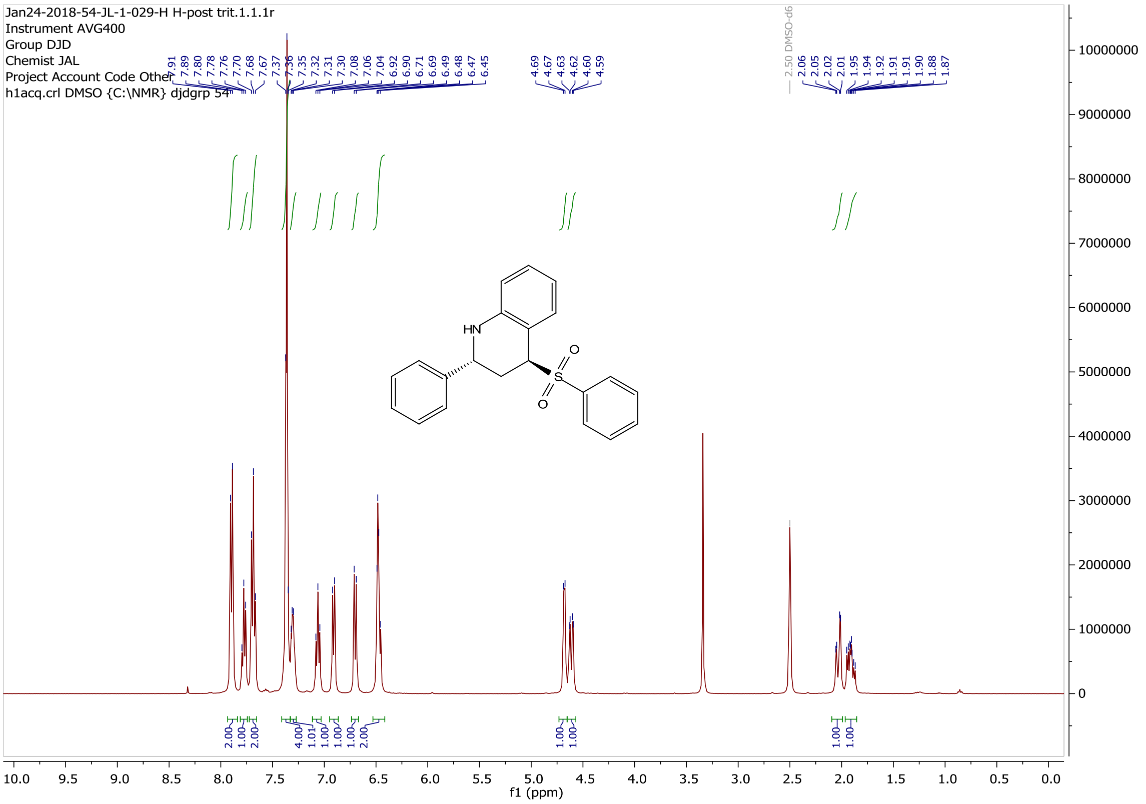

3m – ^{13}C NMR (100 MHz, DMSO-d_6)

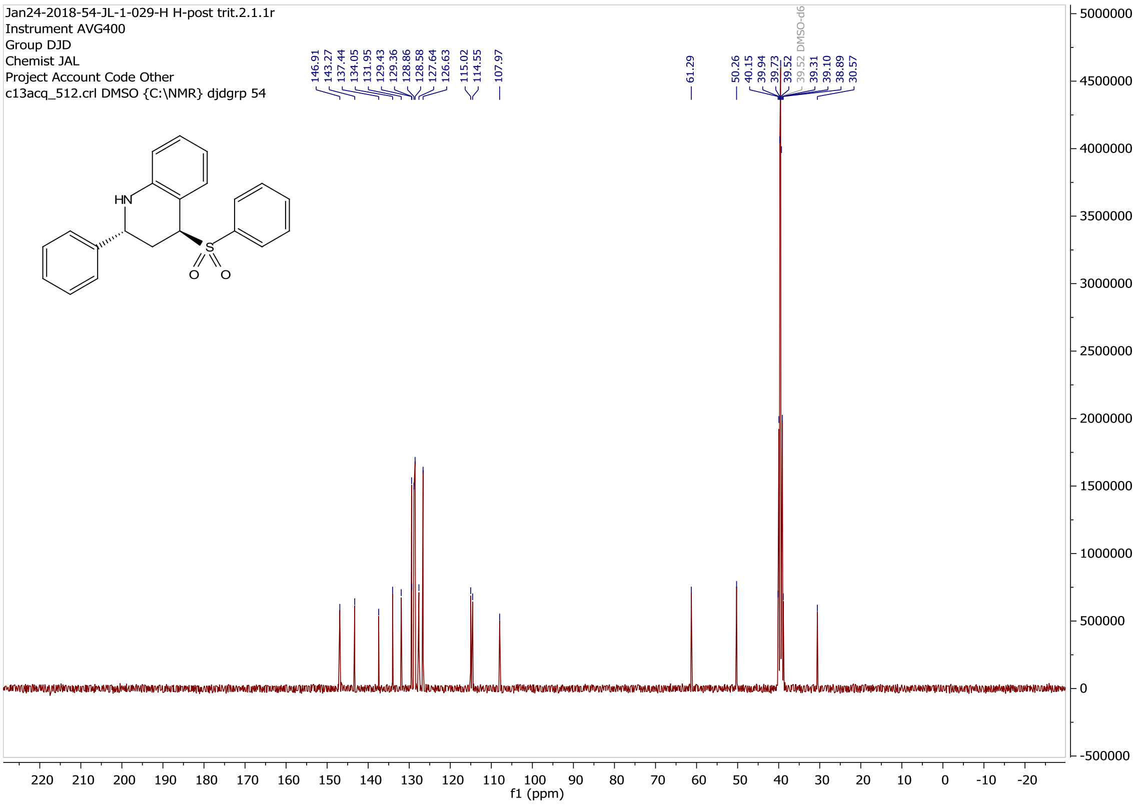

3n – ^{1}H NMR (400 MHz, DMSO-d_6:$CDCl_3$ 1:5)

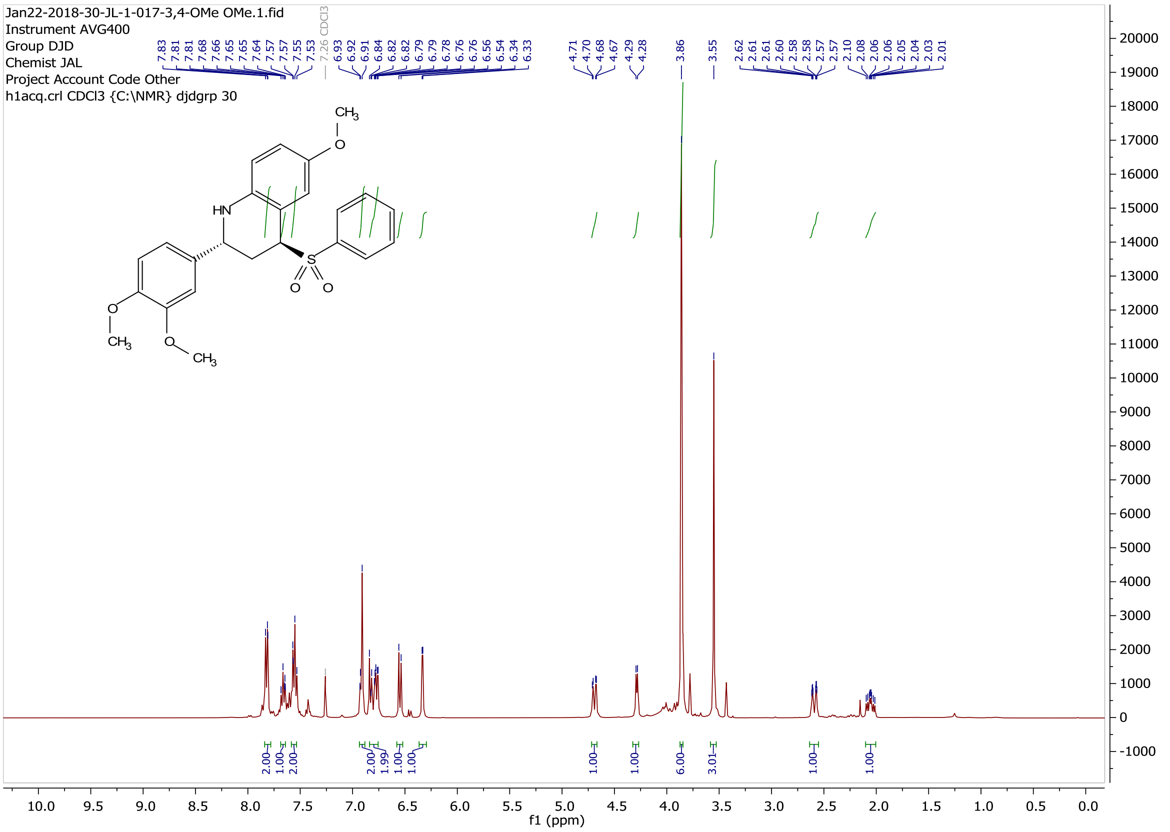

3n – ^{13}C NMR (126 MHz, DMSO-d_6:$CDCl_3$ 1:5)

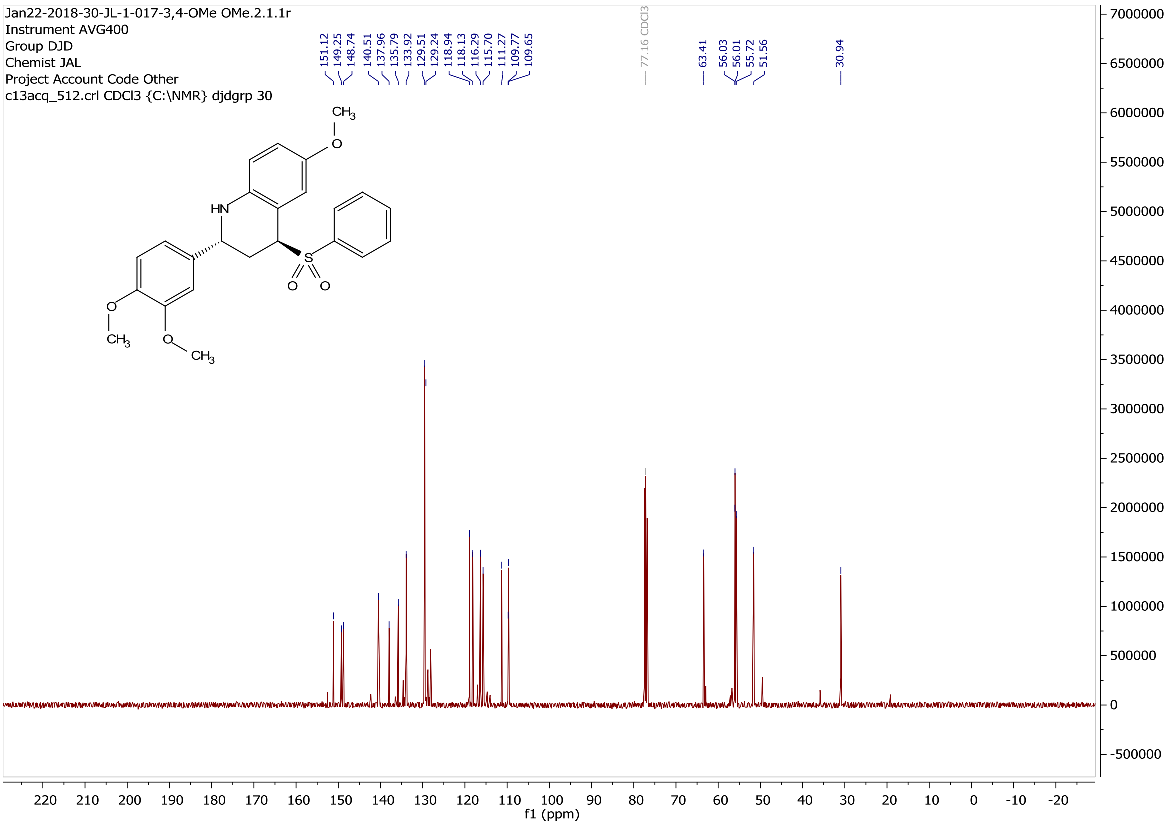

3o – ^{1}H NMR (400 MHz, DMSO-d_6)

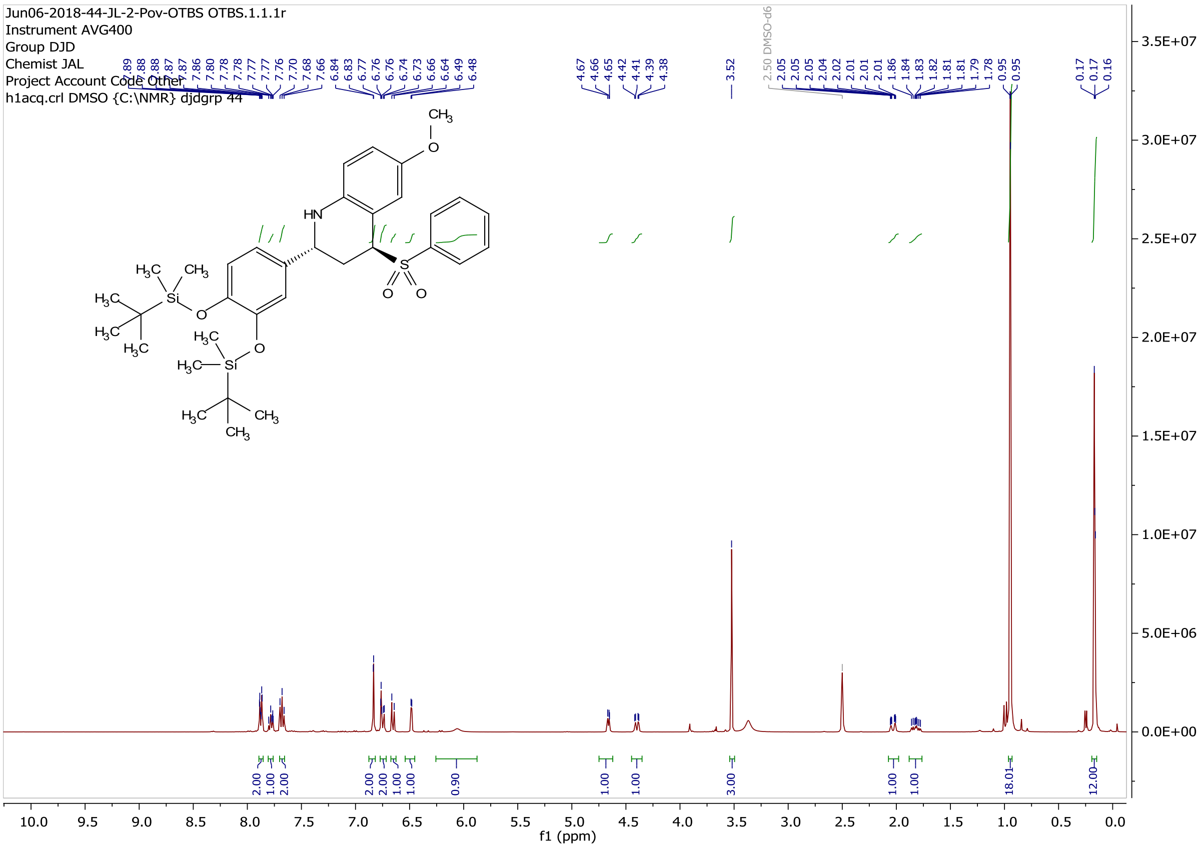

3o – ^{13}C NMR (126 MHz, DMSO-d_6)

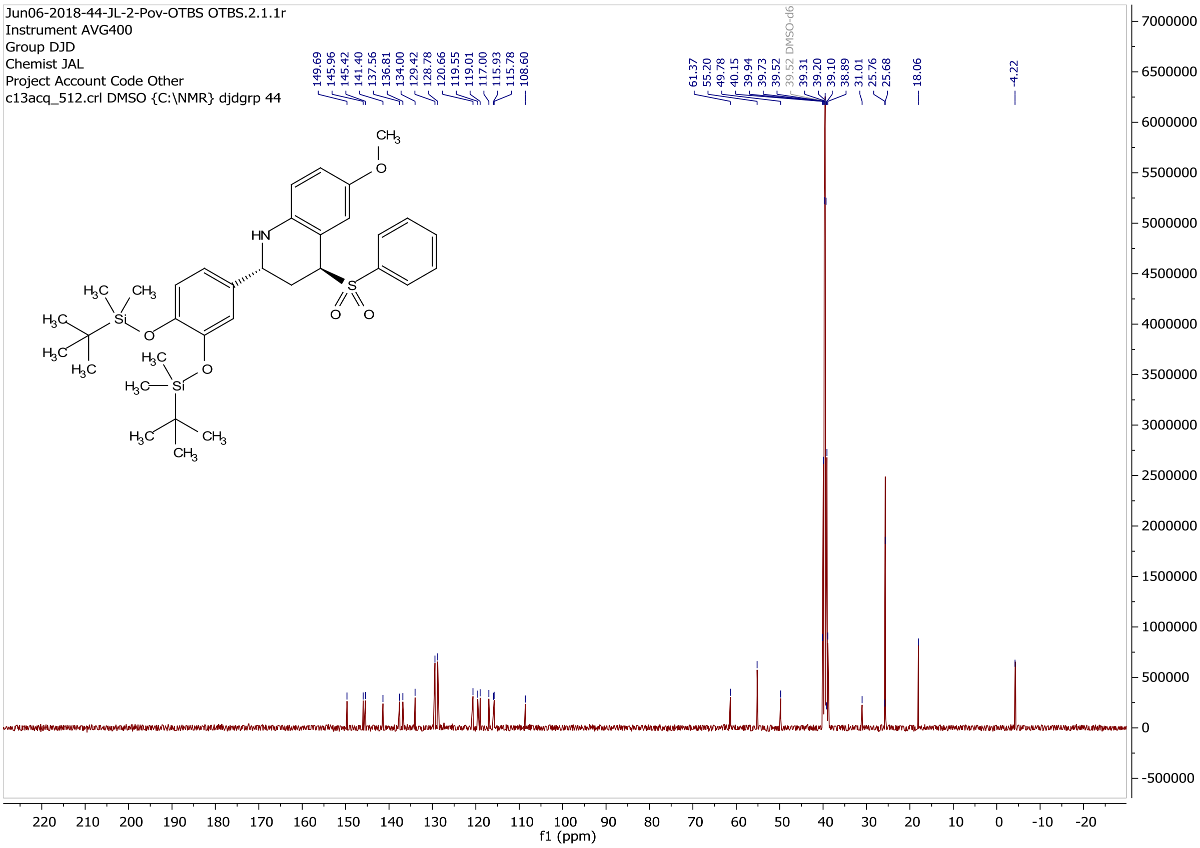

3p – ^{1}H NMR (400 MHz, DMSO-d_6)

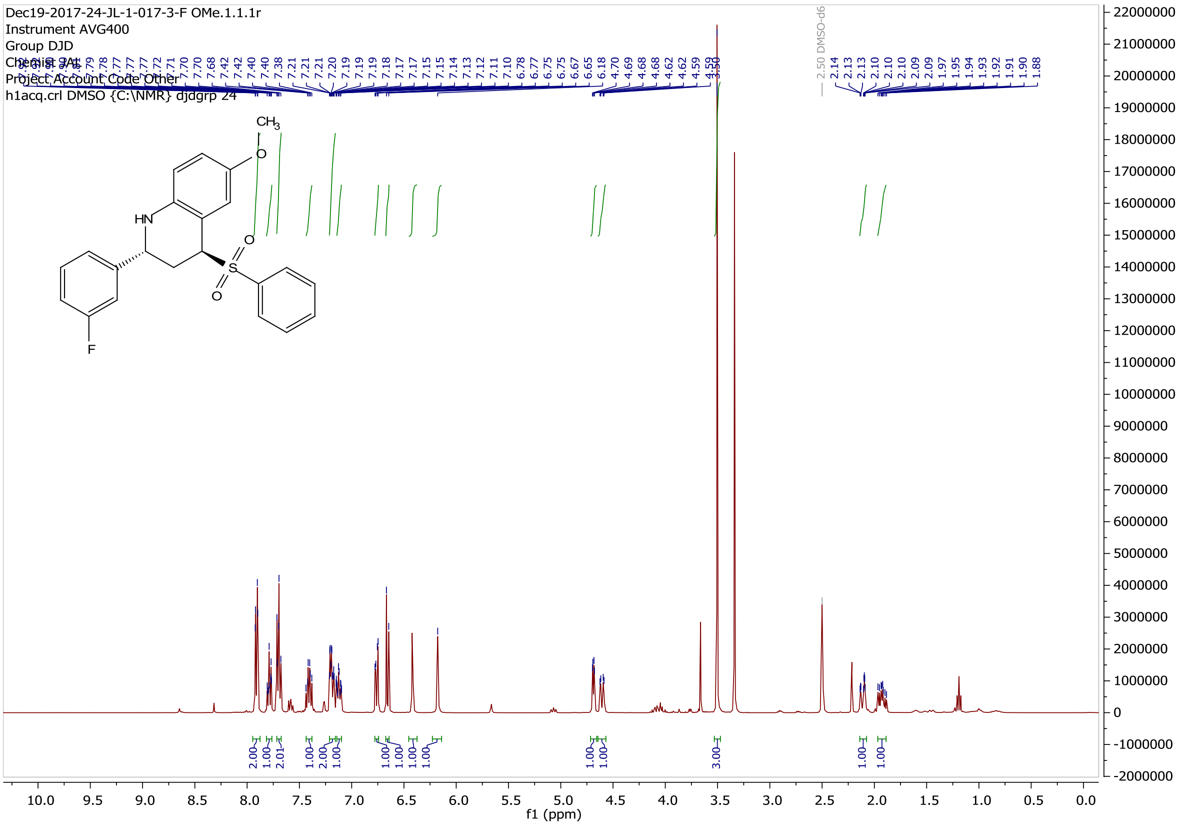

3p -^{19}F NMR (377 MHz, DMSO-d_6)

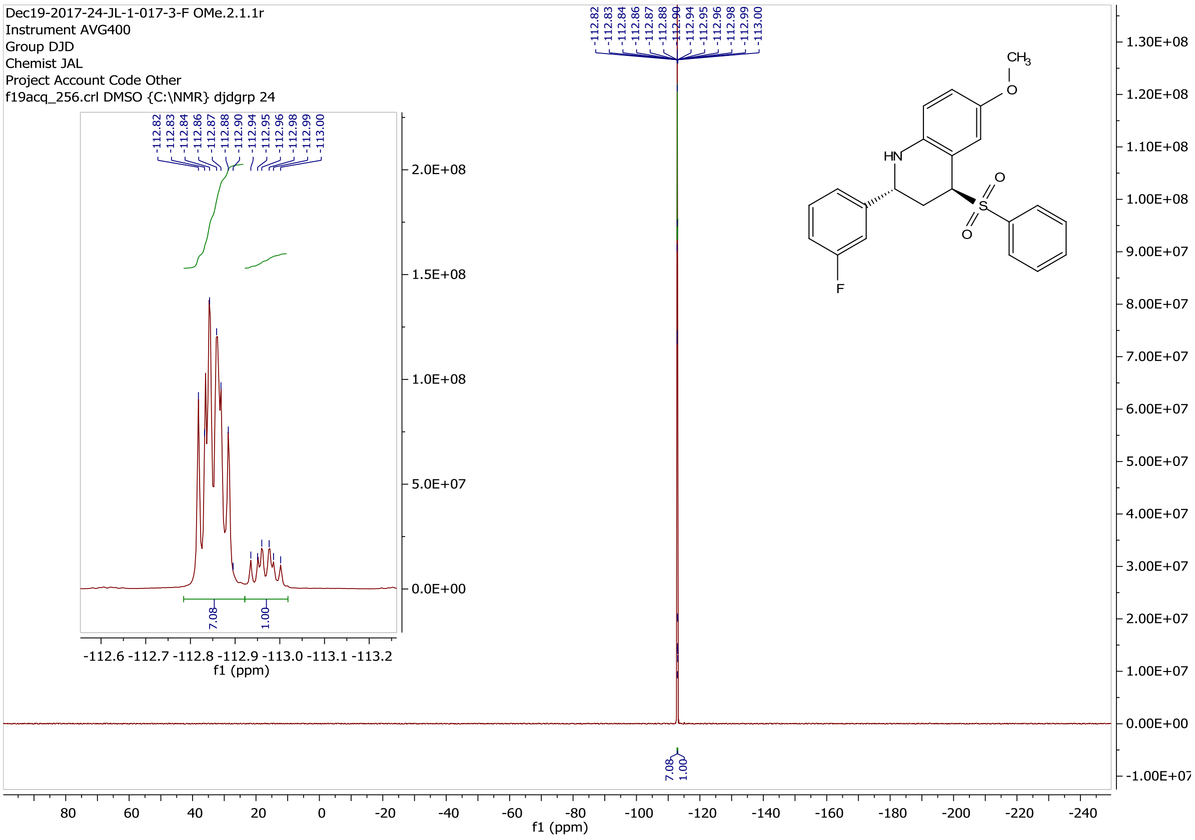

3p – ^{13}C NMR (100 MHz, DMSO-d_6)

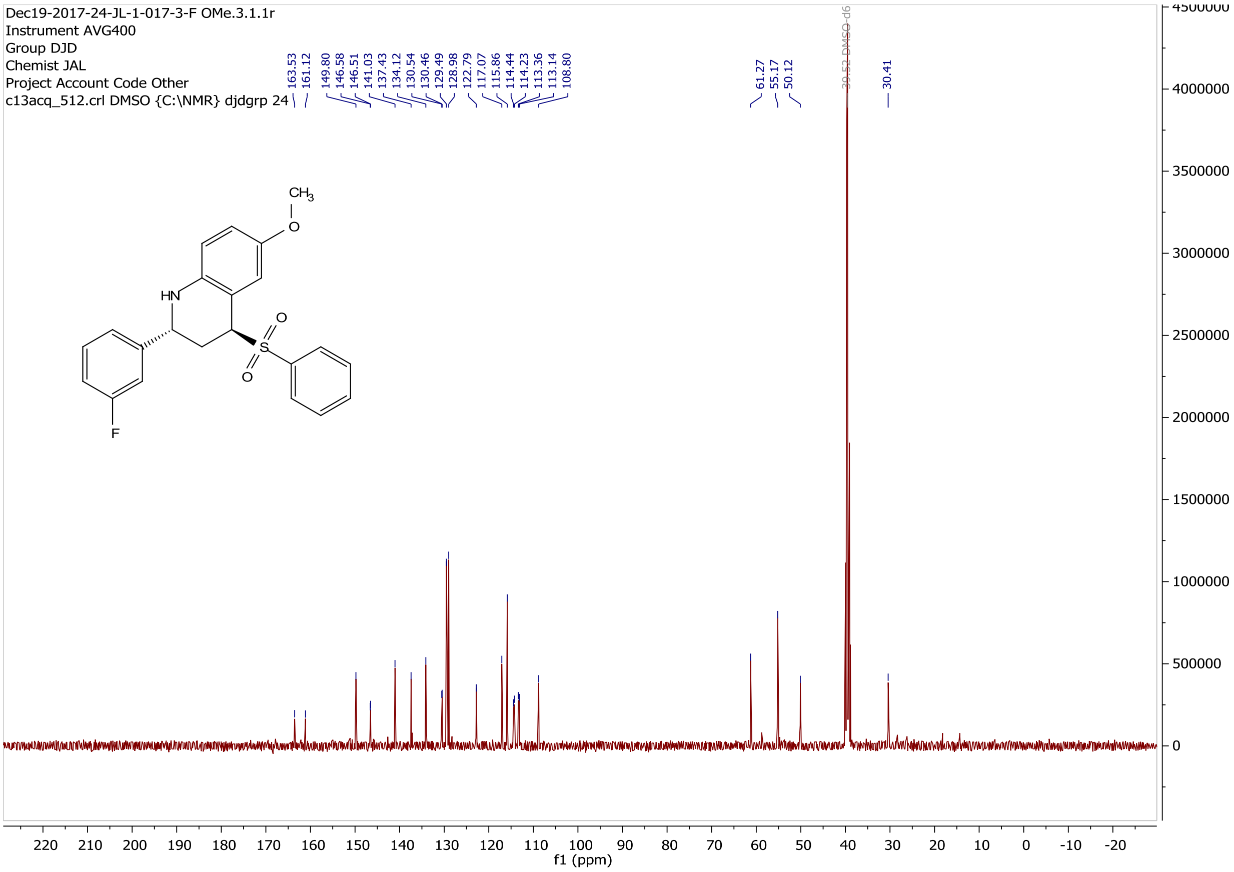

3q – ^{1}H NMR (400 MHz, DMSO-d_6:$CDCl_3$ 1:1)

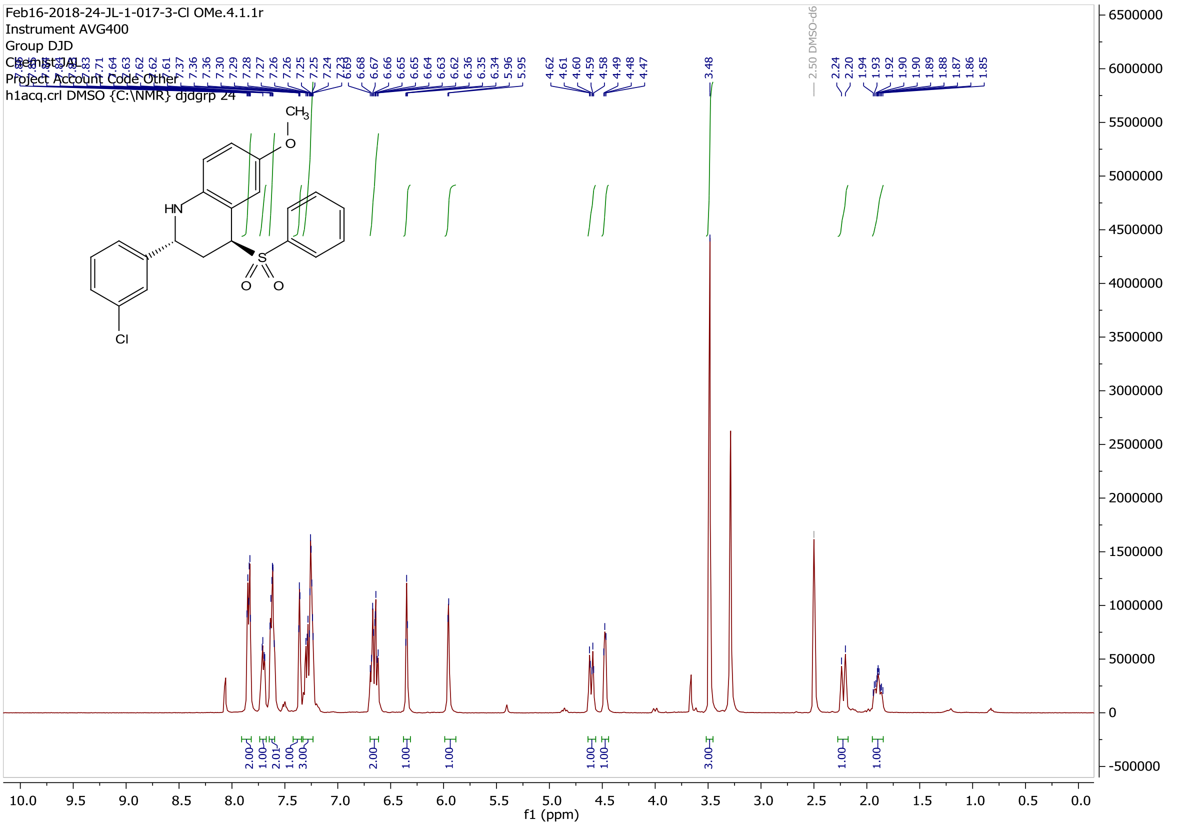

3q –^{13}C NMR (100 MHz, DMSO-d_6:$CDCl_3$ 1:1)

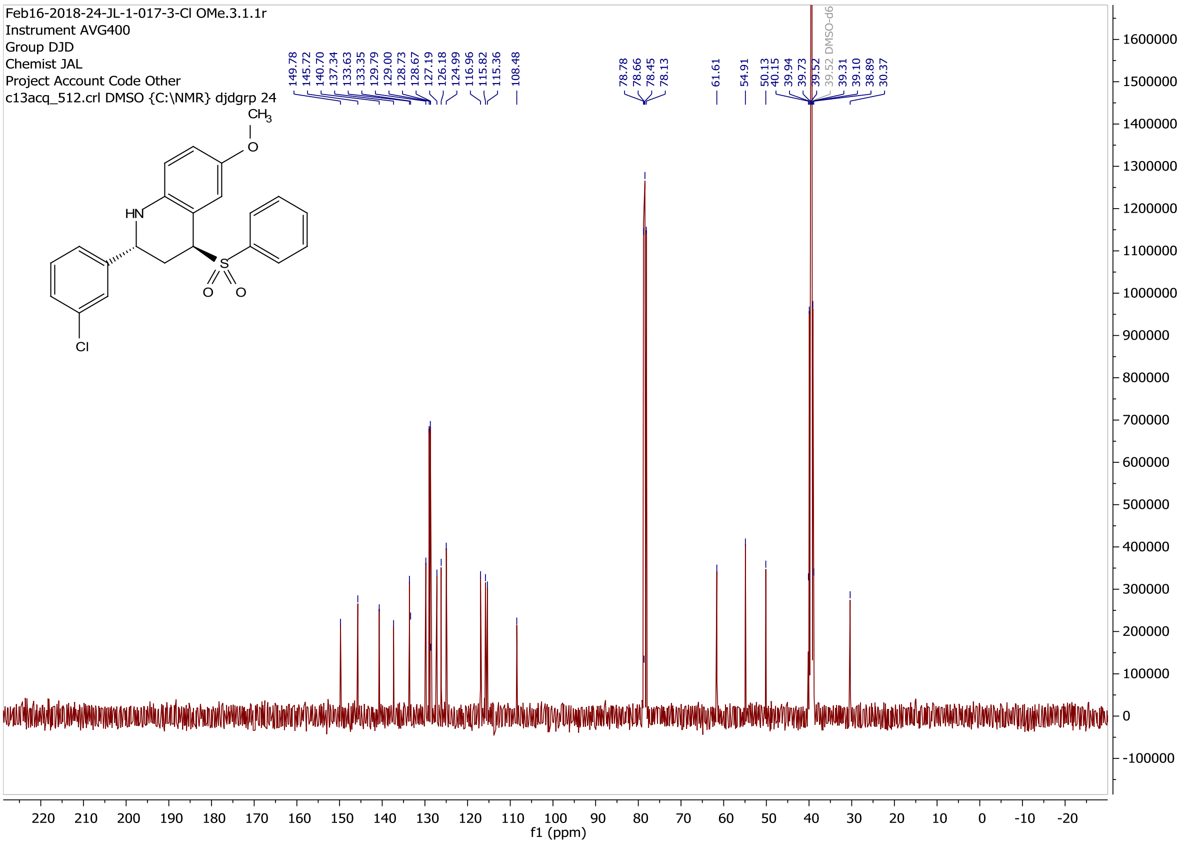

3r – ^{1}H NMR (400 MHz, $CDCl_3$)

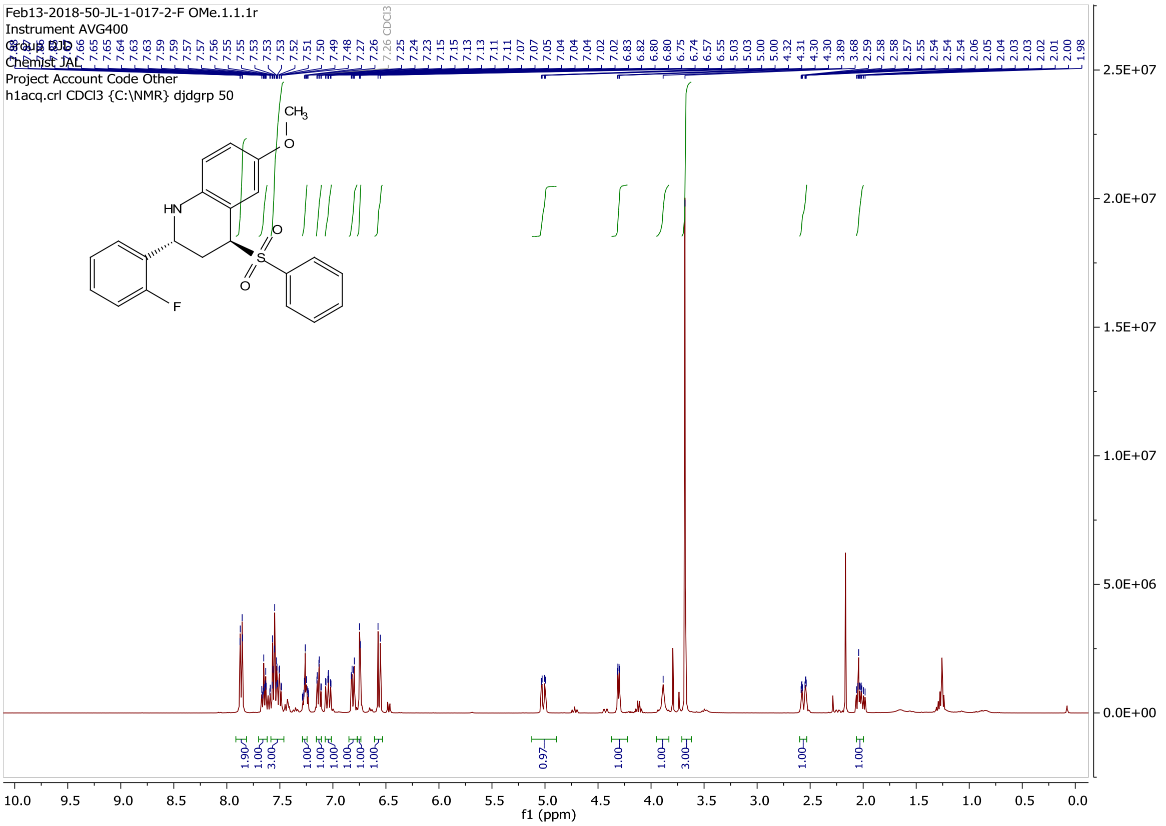

3r – ^{19}F NMR (377 MHz, $CDCl_3$)

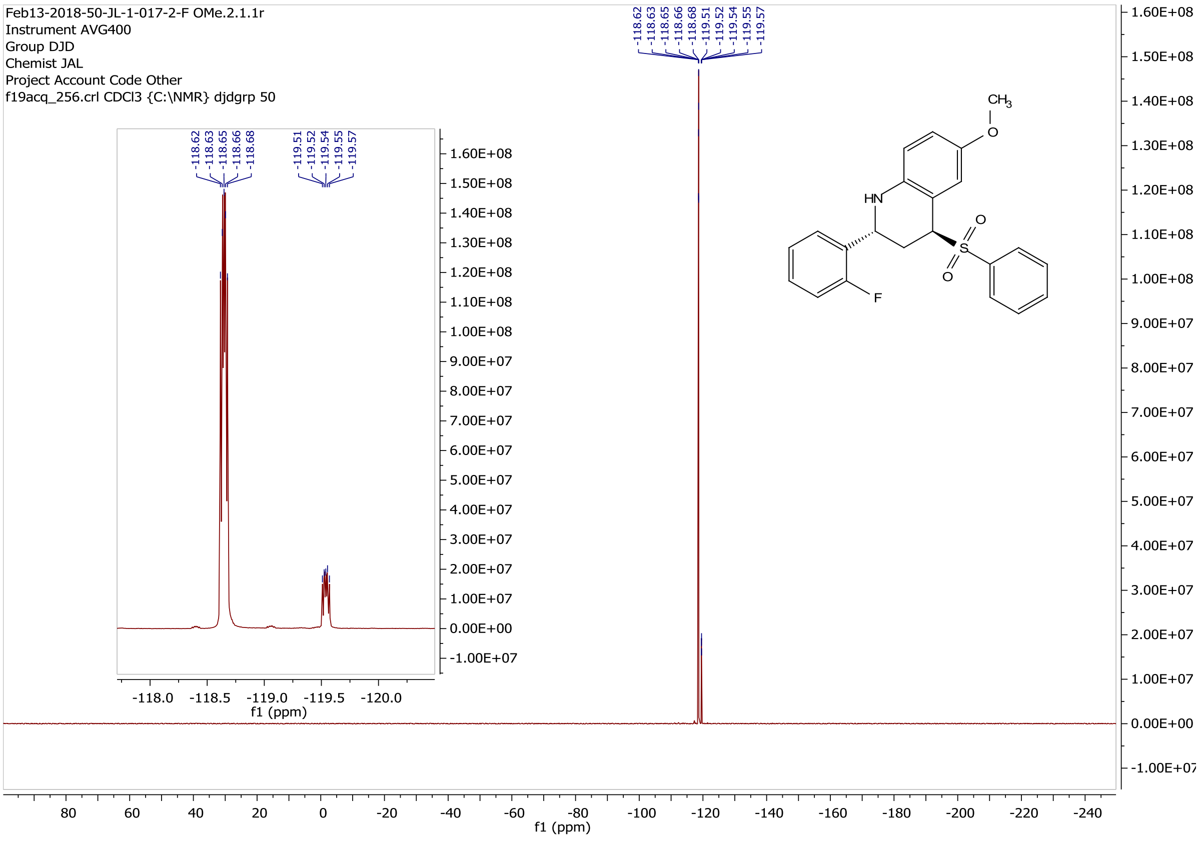

3r – ^{13}C NMR (100 MHz, $CDCl_3$)

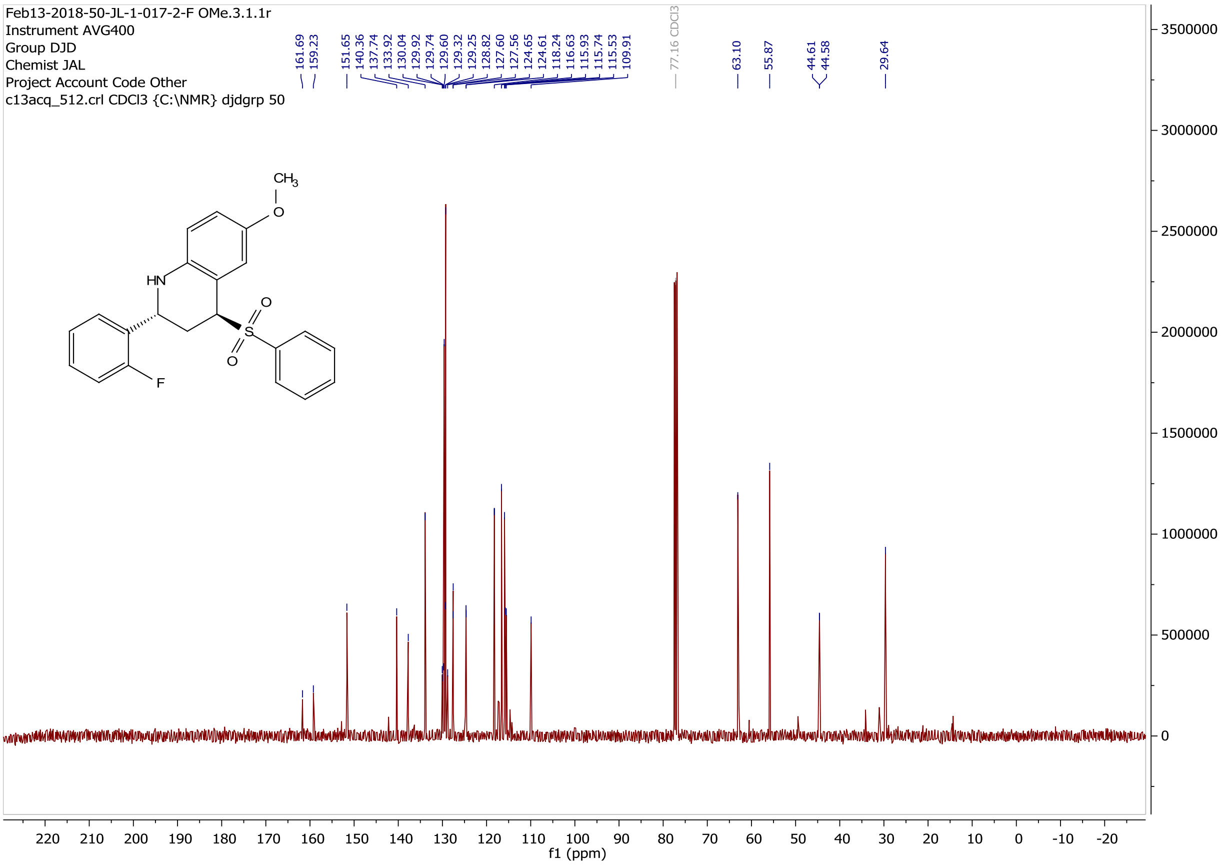

3s – ^{1}H NMR (400 MHz, DMSO-d_6)

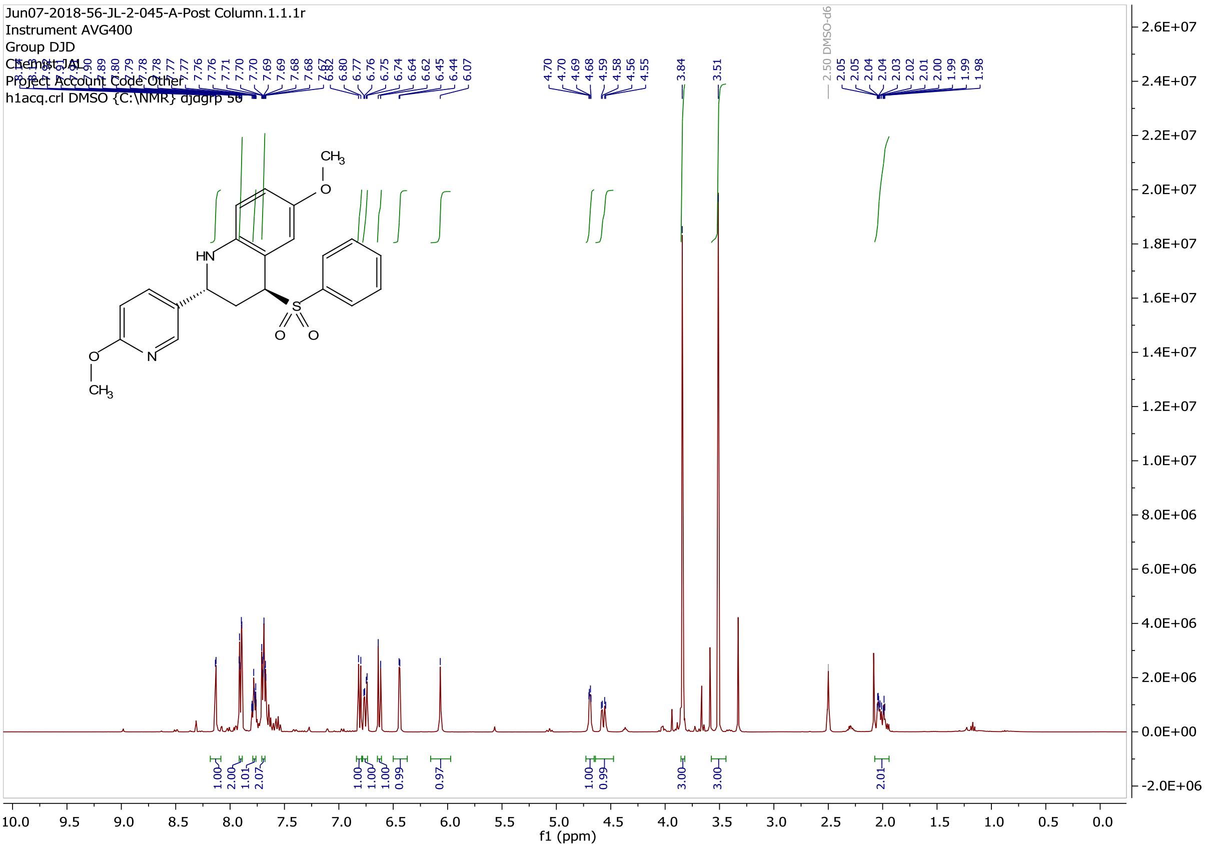

3s – ^{13}C NMR (100 MHz, DMSO-d_6)

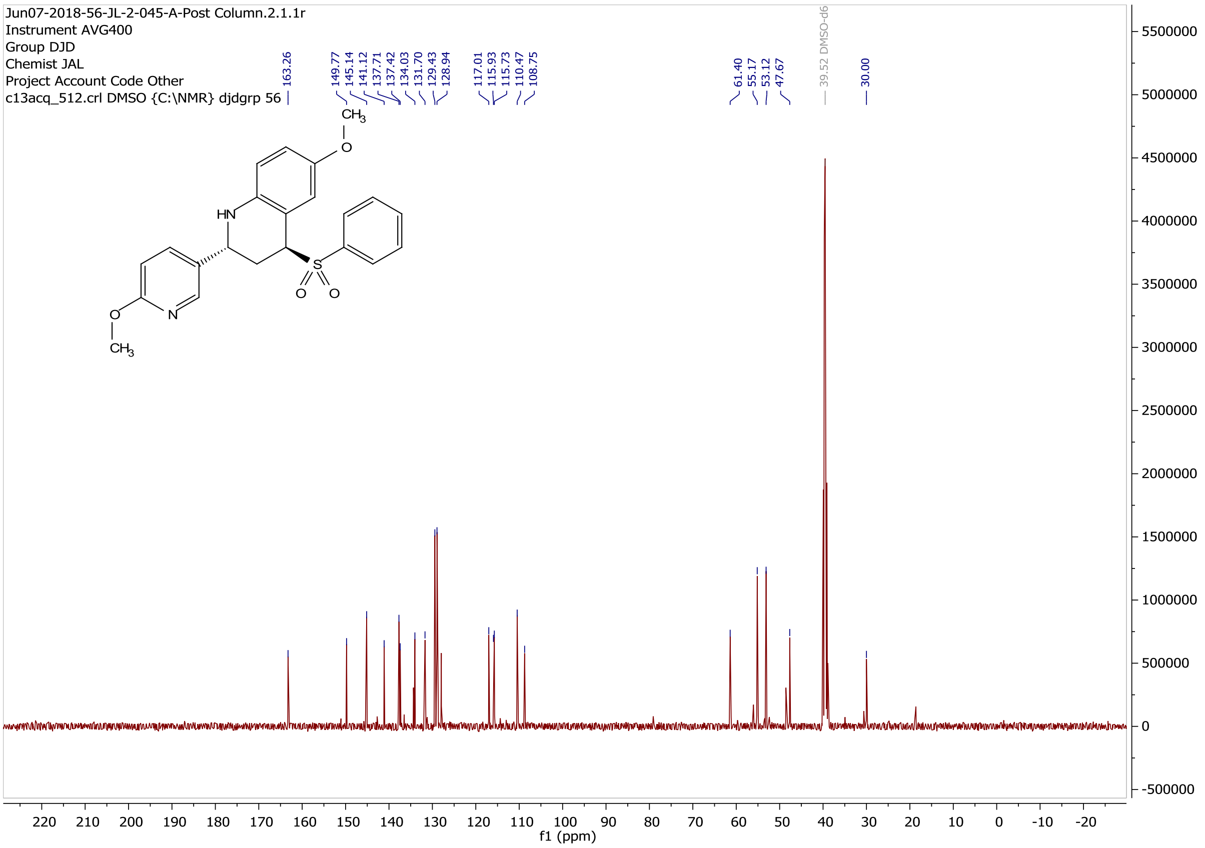

3ab – ^{1}H NMR (400 MHz, DMSO-d_6)

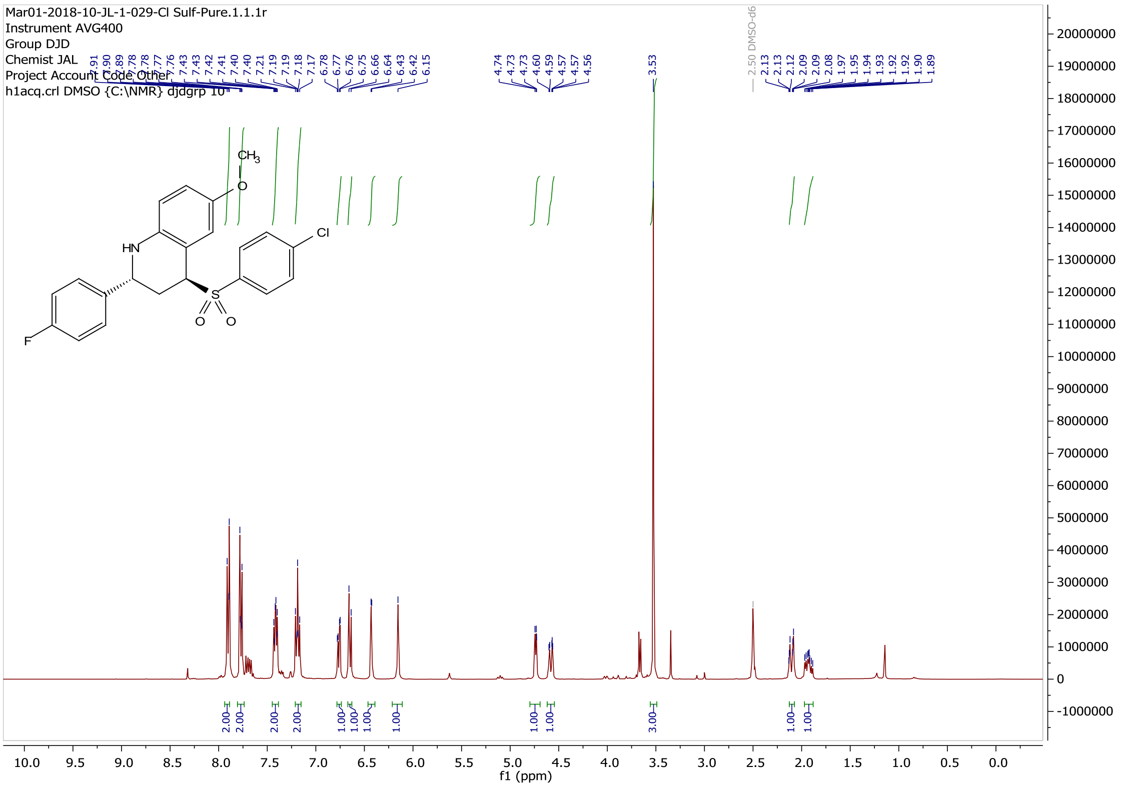

3ab – ^{19}F NMR (377 MHz, DMSO-d_6)

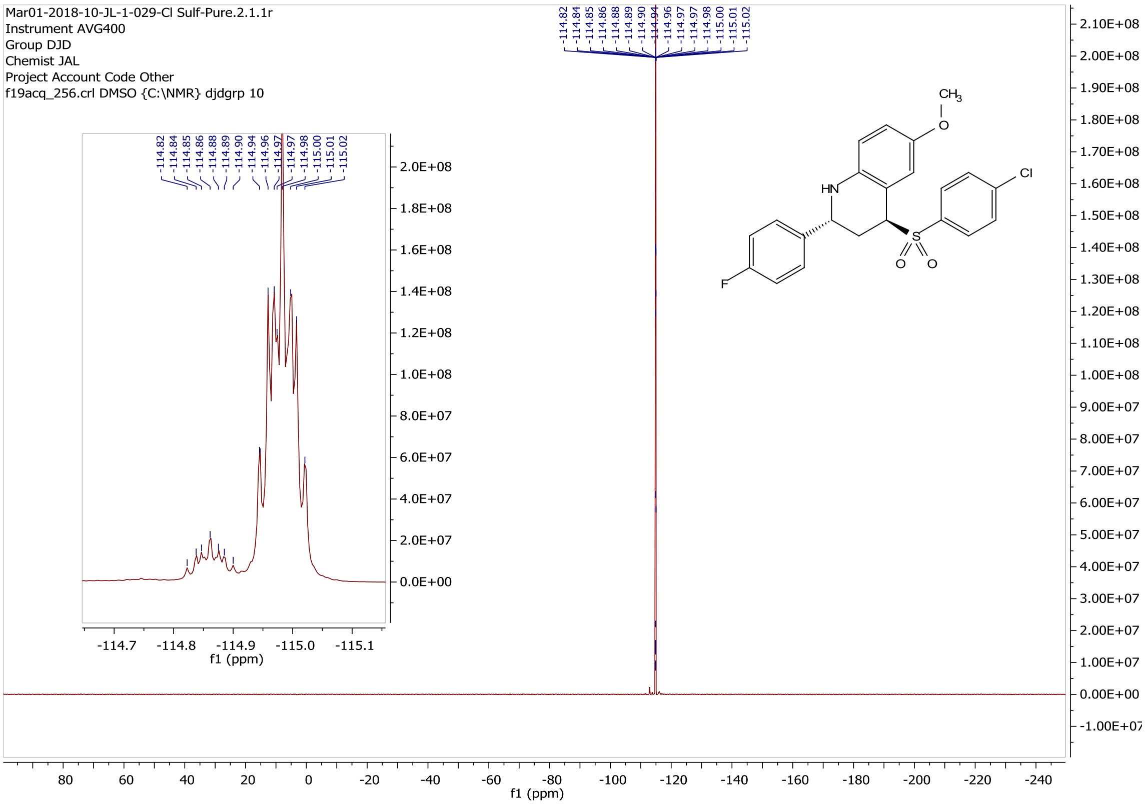

3ab – ^{13}C NMR (100 MHz, DMSO-d_6)

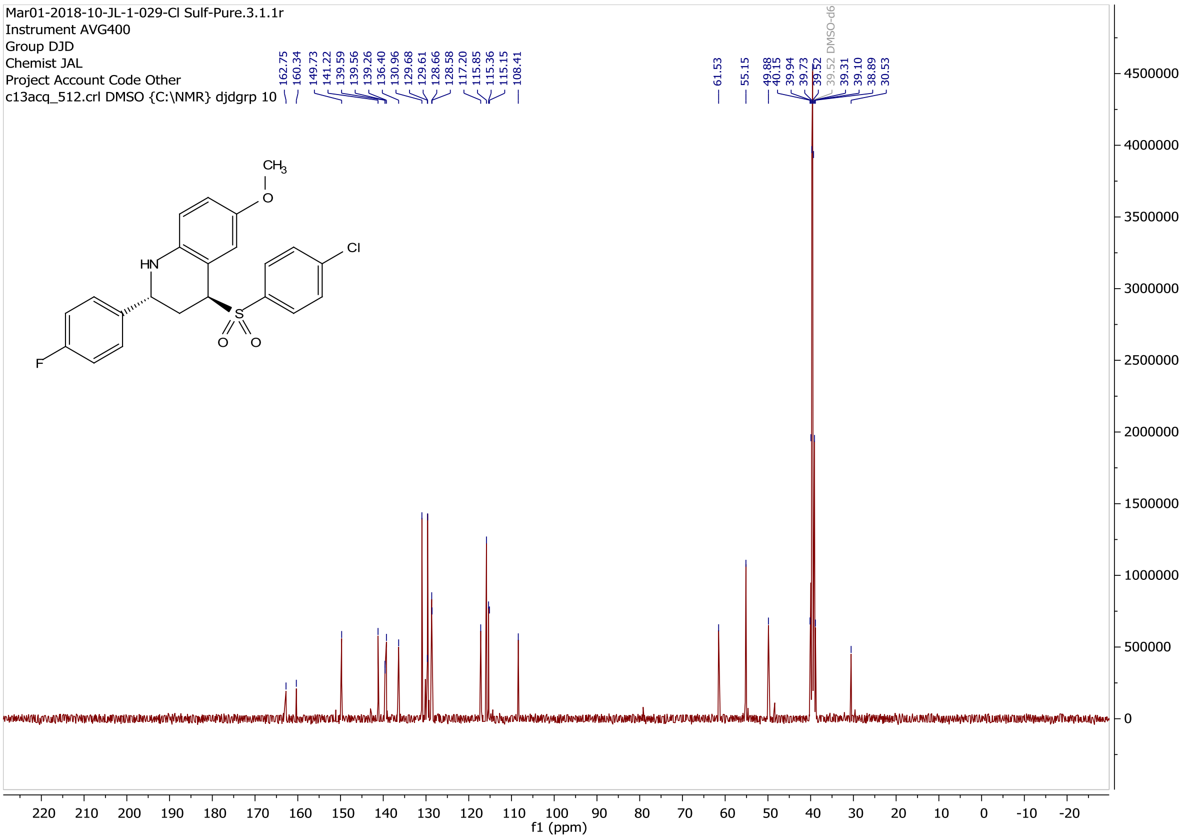

3ac – ^{1}H NMR (400 MHz, DMSO-d_6)

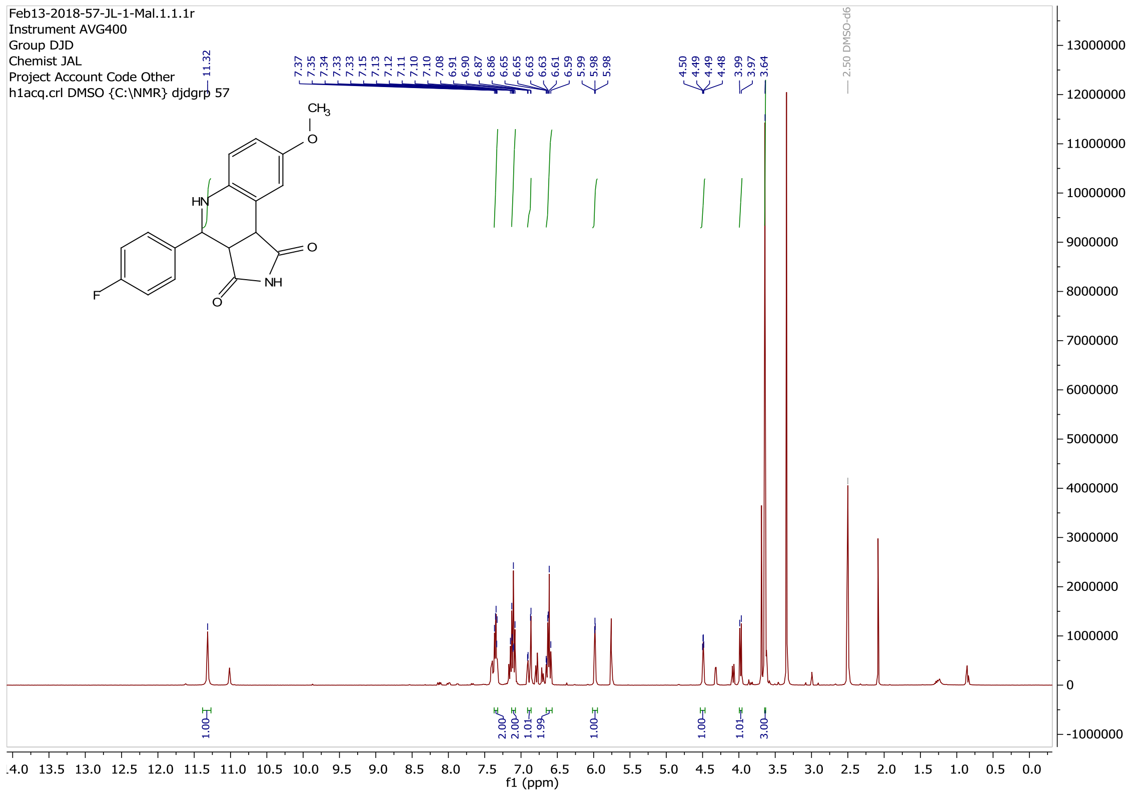

3ac – ^{19}F NMR (377 MHz, DMSO-d_6)

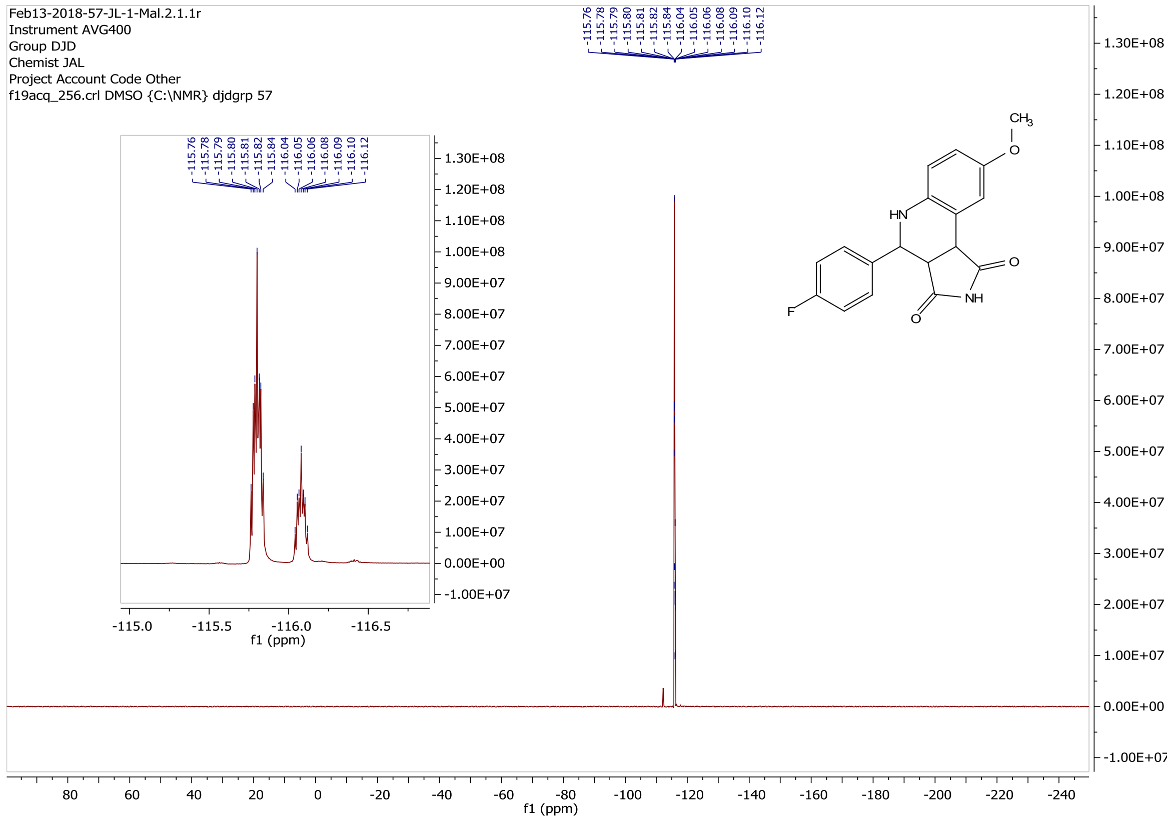

3ac – ^{13}C NMR (100 MHz, DMSO-d_6)

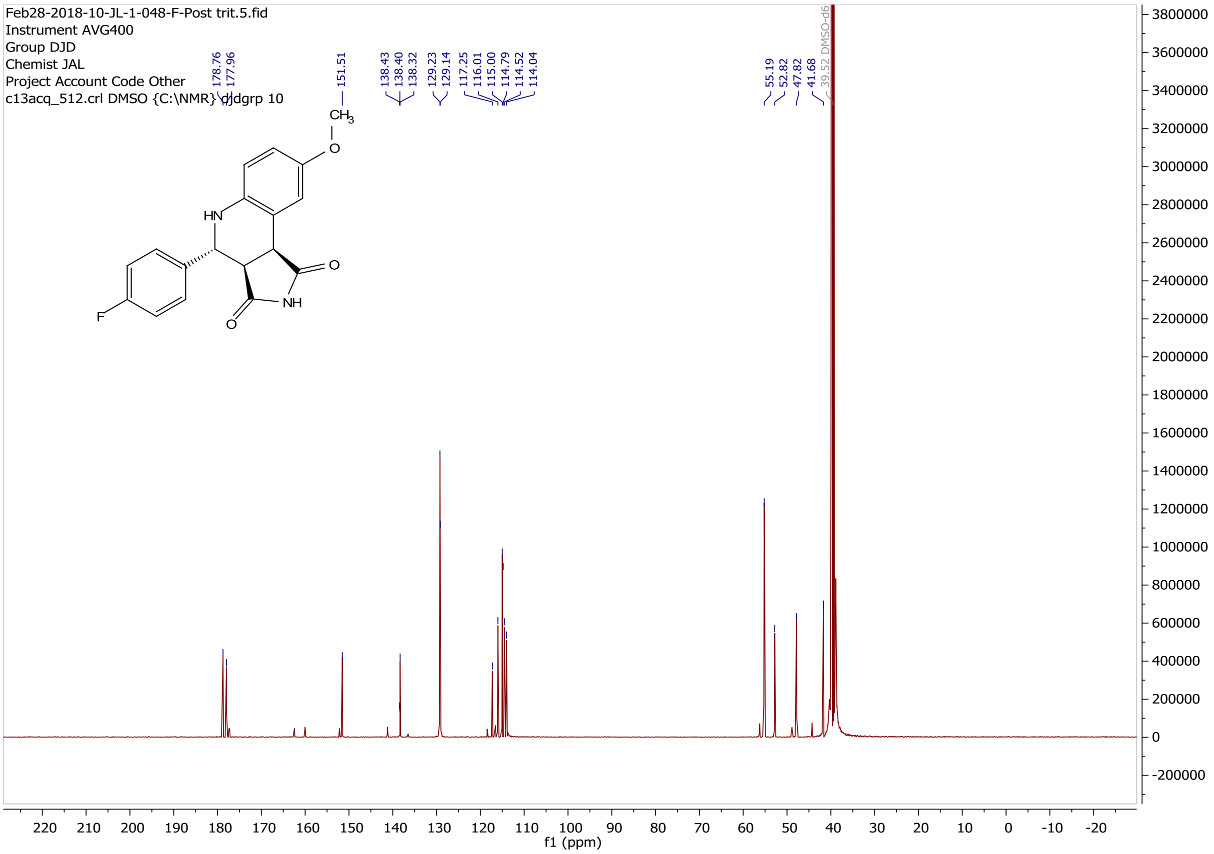

3ad – ^{1}H NMR (400 MHz, DMSO-d_6)

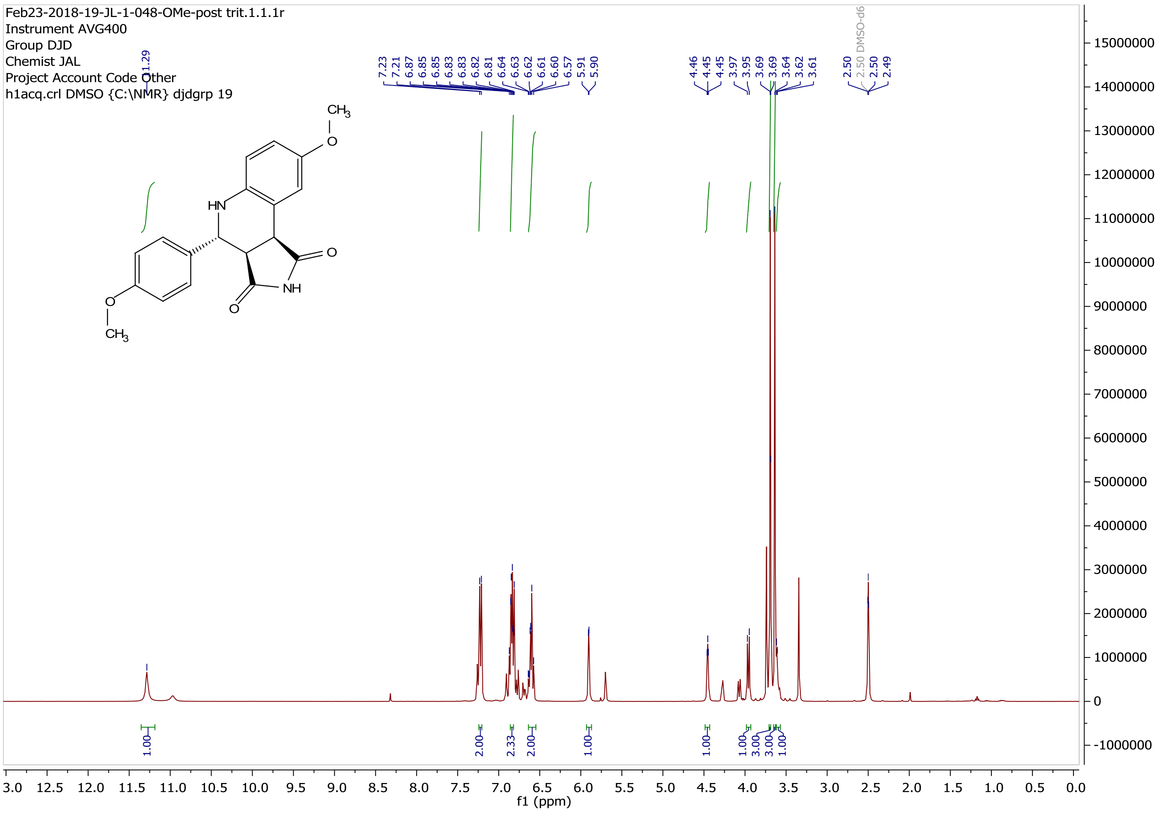

3ad – ^{13}C NMR (100 MHz, DMSO-d_6)

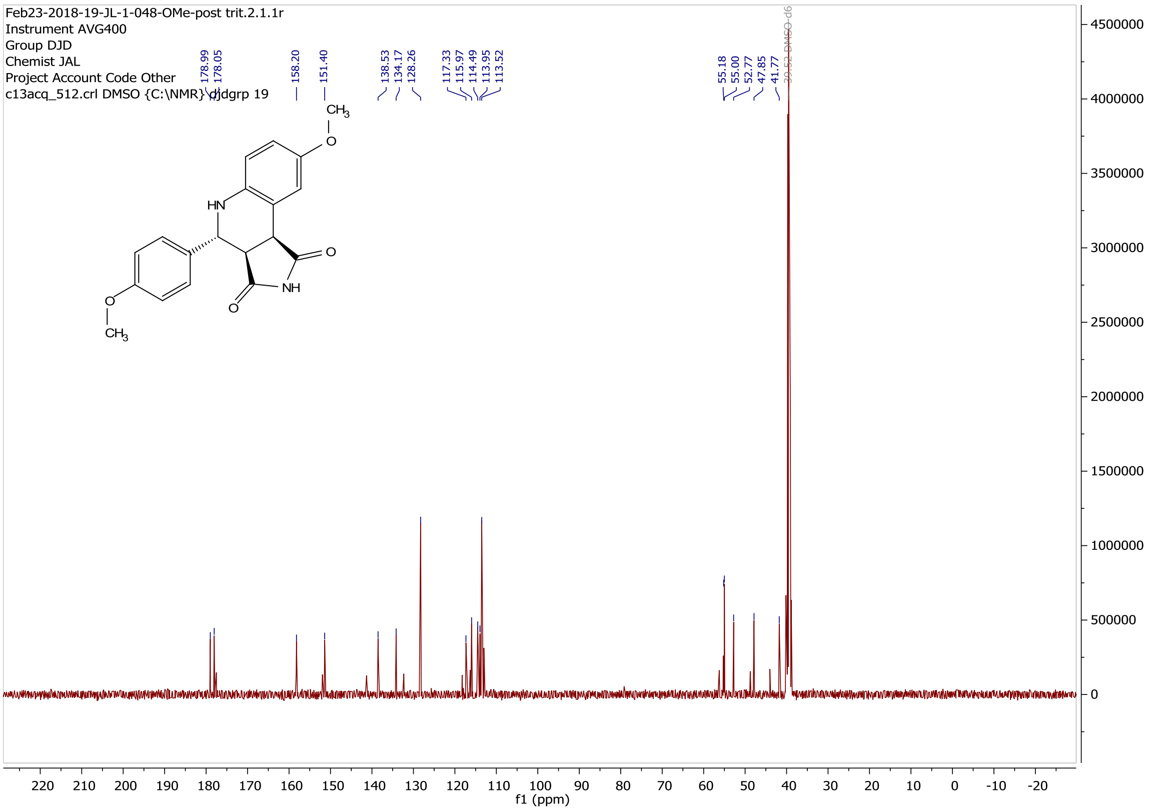

3ae – ^{1}H NMR (400 MHz, DMSO-d_6)

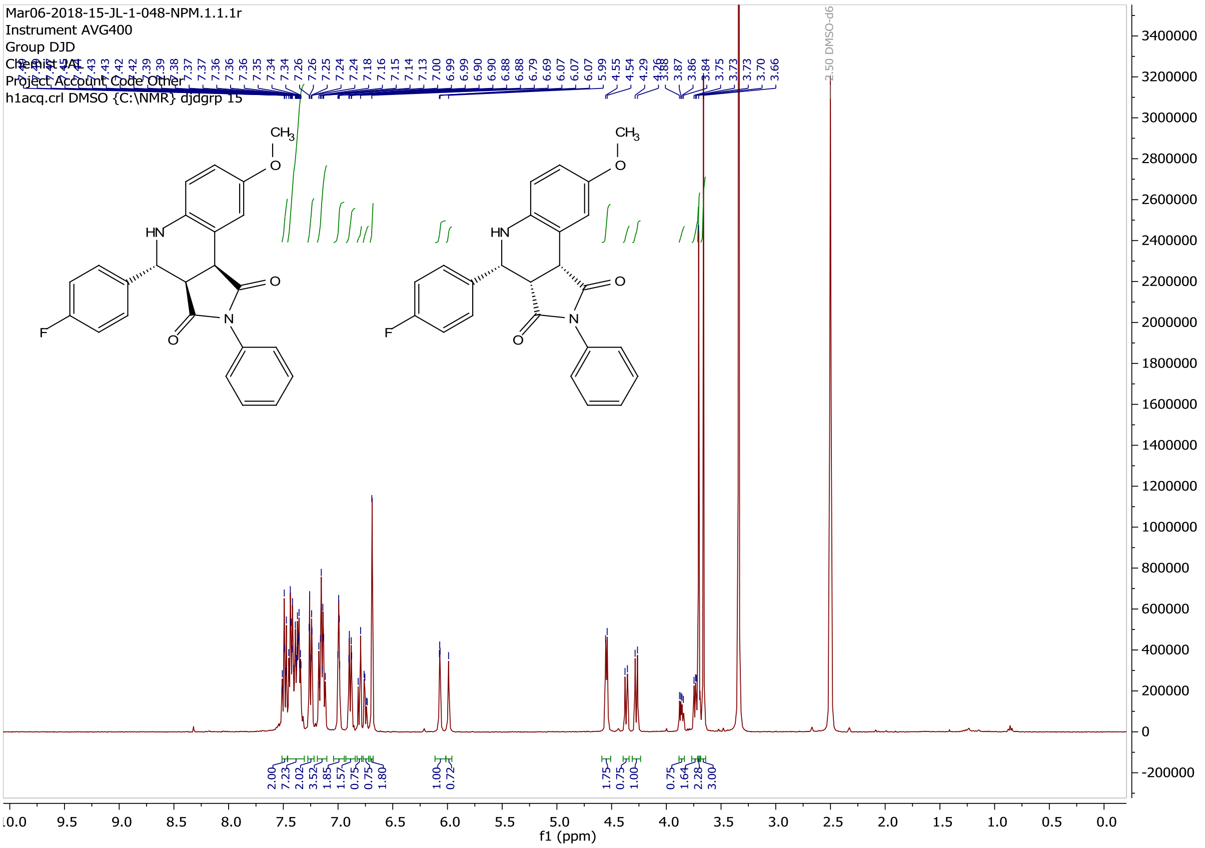

3ae – ^{19}F NMR (377 MHz, DMSO-d_6)

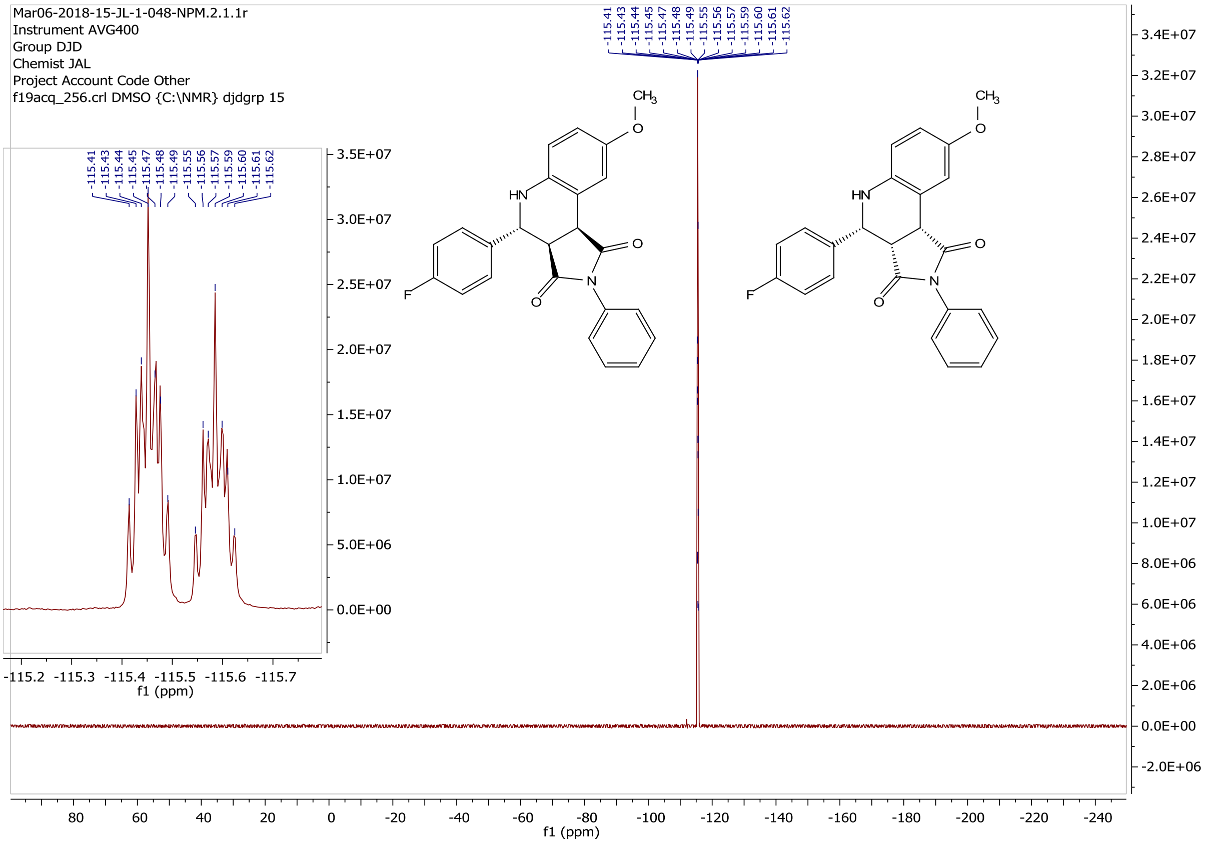

3ae – ^{13}C NMR (100 MHz, DMSO-d_6)

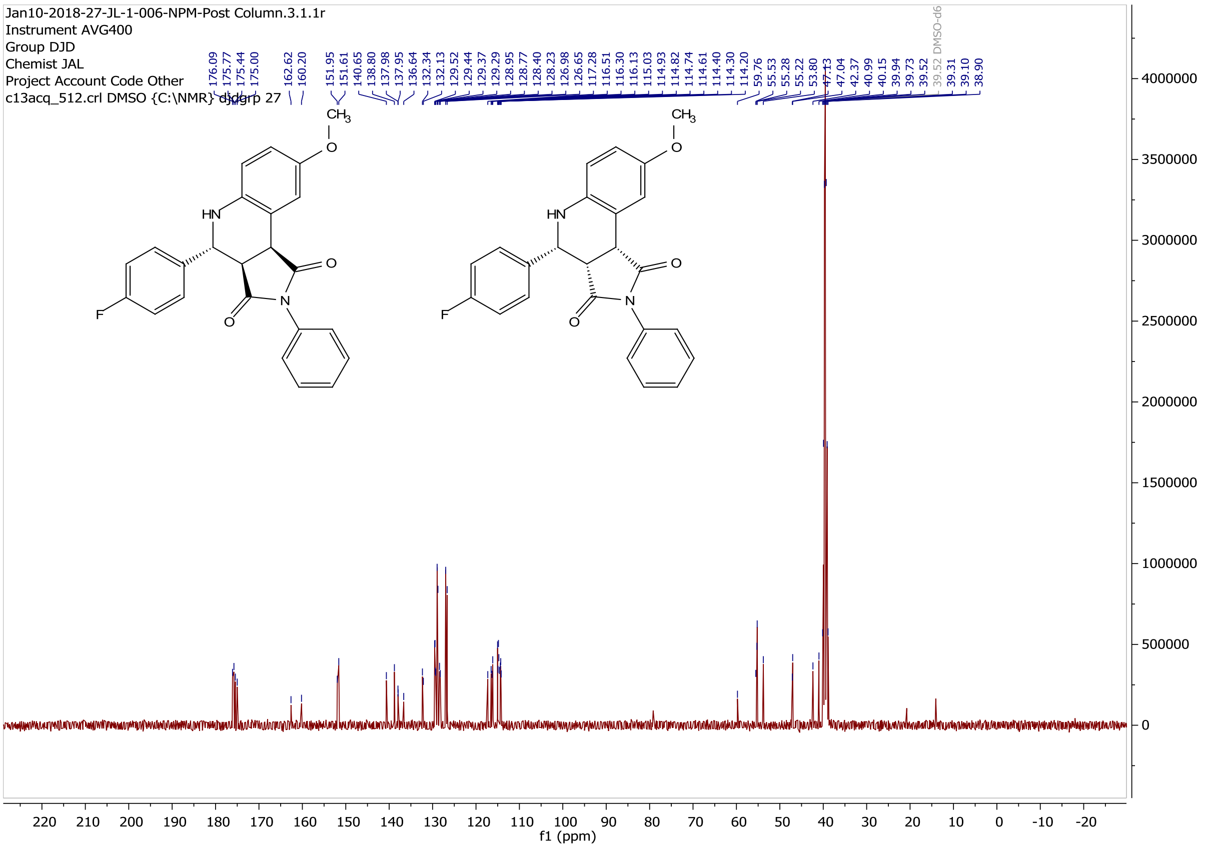

9. References

1. A. L. Fuentes de Arriba, F. Urbitsch, and D. J. Dixon, *Chem. Commun.*, 2016, ***52***, 14434
2. Q. Jiang, J.-Y. Wang, and C. Guo, *J. Org. Chem.*, 2014, ***79***, 8768.
3. T. Schwob, and R. Kempe, *Angew. Chem. Int. Ed.*, 2016, ***55***, 15175.
4. R. L. Zuckerman, S. W. Krska, and R. G. Bergman, *J. Am. Chem. Soc.*, 2000, ***122***, 751.
5. M. Mastalir, M. Glatz, N. Gorgas, B. Stoger, and E. Pittenauer, G. Allmaier, L. F. Veros, K. Kirchner, *Chem. Eur. J.*, 2016, ***22***, 12316.
6. S. Saranya, R. Ramesh, J. and G. Malecki, *Eur. J. Org. Chem.*, 2017, ***2017***, 6726.
7. T. Higuchi, R. Tagawa, A. Iimuro, S. Akiyama, H. Nagae, and K. Mashima, *Chem. Eur. J.*, 2017, ***23***, 12795.
8. M. Largeron, and M.-B. Fleury, *Chem. Eur. J.*, 2017, ***23***, 6763.
9. A. M. Seayad, B. Ramalingam, K. Yoshinaga, and T. Nagata, C. L. L. Chai, *Org. Lett.*, 2010, ***12***, 264.
10. L. Liu, C. Wang, Q. Liu, Y. Kong, W. Chang, and J. Li, *Eur. J. Org. Chem.*, 2016, ***2016***, 3684.
11. L. Han, P. Xing, and B. Jiang, *Org. Lett.*, 2014, ***16***, 3428.
12. L. Jiang, L. Jin, H. Tian, X. Yuan, X. Yu, and Q. Xu, *Chem. Commun.*, 2011, ***47***, 10833.
13. D. Kumar, M. Sonawane, B. Pujala, V. K. Jain, and S. Bhagat, A. K. Chakraborti, *Green Chem.*, 2013, ***15***, 2872.
14. C. Cao, B. Lu, and G. Chen, *J. Phys. Org. Chem.*, 2011, ***24***, 335.
15. Y.-F. Zhang, B. Wu, and Z.-J. Shi, *Chem. Eur. J.*, 2016, ***22***, 17808.
16. M. A. Chilleck, L. Harstenstein, T. Braun, P. W. Roesky, and B. Braun, *Chem. Eur. J.*, 2015, ***21***, 2594.
17. J. Gao, S. Bhunia, K. Wang, L. Gan, S. Xia, and D. Ma, *Org. Lett.*, 2017, ***19***, 2809.
18. H. Niaz, H. Kashtoh, J. A. J. Khan, A. Khan, A.-T. Wahab, M. T. Alam, K. M. Khan, S. Perveen, and M. I. Choudhary, *Eur. J. Med. Chem.*, 2015, ***95***, 199.
19. M. C. Bagley, V. Fusillo, R. L. Jenkins, M. C. Lubinu, and C. Mason, *Beilstein J. Org. Chem.*, 2013, ***9***, 1957.
20. D. S. Rekunge, C. K. Khatri, and G. U. Chaturbhuj, *Tetrahedron Lett.*, 2017, ***58***, 1240.
21. T. Hajiashrafi, M. Karimi, A. Heydari, and A. A. Tehrani, *Catal. Lett.*, 2017, ***147***, 453.
22. G. Li, R. Chen, L. Wu, Q. Fu, X. Zhang, and Z. Tang, *Angew. Chem. Int. Ed.*, 2013, ***52***, 8432.
23. G. B. Choi, Q. Zhu, D. C. Miller, C. J. Gu, and R. R. Knowles, *Nature*, 2016, ***539***, 268.
24. D. N. Primer, I. Karakaya, J. C. Tellis, and G. A. Molander, *J. Am. Chem. Soc.*, 2015, ***137***, 2195.
25. http://brsmblog.com/wp-content/uploads/2014/09/DiRocco-Electrochemical-Series.png
26. M. A. Cismesia and T. P. Yoon, *Chem. Sci.*, 2015, **6**, 5426.
